# Mitogenomic sequences effectively recover relationships within brush-footed butterflies (Lepidoptera: Nymphalidae)

**DOI:** 10.1186/1471-2164-15-468

**Published:** 2014-06-12

**Authors:** Li-Wei Wu, Li-Hung Lin, David C Lees, Yu-Feng Hsu

**Affiliations:** Department of Geosciences, National Taiwan University, Taipei, Taiwan; Department of Zoology, University of Cambridge, Cambridge, UK; Department of Life Science, National Taiwan Normal University, Taipei, Taiwan

**Keywords:** *Athyma*, Bayes factor, DNA Barcoding, *cox1* gene, Limenitidinae, Limenitidini, Mitogenomic phylogeny, Papilionoidea, Pyrosequencing

## Abstract

**Background:**

Mitogenomic phylogenies have revealed well-supported relationships for many eukaryote groups. In the order Lepidoptera, 113 species mitogenomes had been sequenced (May 14, 2014). However, these data are restricted to ten of the forty-three recognised superfamilies, while it has been challenging to recover large numbers of mitogenomes due to the time and cost required for primer design and sequencing. Nuclear rather than mitochondrial genes have been preferred to reconstruct deep-level lepidopteran phylogenies, without seriously evaluating the potential of entire mitogenomes. Next-generation sequencing methods remove these limitations by providing efficiently massive amounts of sequence data. In the present study, we simultaneously obtained a large number of nymphalid butterfly mitogenomes to evaluate the utility of mitogenomic phylogenies by comparing reconstructions to the now quite well established phylogeny of Nymphalidae.

**Results:**

We newly obtained 30 nymphalid mitogenomes via pyrosequencing on the Roche 454 GS Junior system, and combined these sequences with publicly accessible data to provide a 70-taxa dataset covering 37 genes for a 15,495 bp alignment. Polymorphic sites were not homogeneously distributed across the gene. Two gene regions, *nad6* and 3’ end of *nad5*, were most variable, whereas the *cox1* and 5’ ends of *rrnL* were most conserved. Phylogenetic relationships inferred by two likelihood methods were congruent and strongly supported (>0.95 posterior probability; ML bootstrap >85%), across the majority of nodes for multiple partitioning strategies and substitution models. Bayes factor results showed that the most highly partitioned dataset is the preferred strategy among different partitioning schemes. The most striking phylogenetic findings were that the subfamily Danainae not Libytheinae was sister of the remaining brush-footed butterflies and that, within Limenitidini, the genus *Athyma* was clearly polyphyletic. None of the single-gene phylogenies recovered the highly supported topologies generated on the basis of the whole mitogenomic data.

**Conclusions:**

Thirty mitogenomes were assembled with 89% completeness from the contigs of pyrosequencing-derived reads. Entire mitogenomes or higher-quality sequences could be obtained by increasing pyrosequencing read coverage or by additional Sanger sequencing. Our mitogenomic phylogenies provide robust nodal support at a range of levels, demonstrating that mitogenomes are both accurate and efficient molecular markers for inferring butterfly phylogeny.

**Electronic supplementary material:**

The online version of this article (doi:10.1186/1471-2164-15-468) contains supplementary material, which is available to authorized users.

## Background

Eukaryotic mitochondria are monophyletic and originate from the bacterial phylum Alphaproteobacteria [1, 2]. One of the descendant lineages, the animal mitochondrion, contains a circular DNA molecule around 16 kb in length. It comprises 22 transfer RNAs (tRNAs), 13 protein-coding genes (PCGs), two ribosomal RNAs (rRNAs), and one or more non-coding regions including the control region [3]. Mitochondrial sequences have been the most popular genetic markers for many types of studies, including disease detection [4], species identification [5], phylogeography [6, 7], and phylogenetics [8, 9].

Mitogenomic data are effective for revealing higher-level relationships of diverse animal groups [9–18]. Although Sanger sequencing has been used to obtain high-quality mitogenomic sequences, the time required for primer design, and the cost invested to recover large numbers of mitogenomic sequences remains challenging. Recently, next-generation sequencing (NGS) methods have been shown to overcome such shortcomings [19]. This makes practical the task of re-examining and re-evaluating phylogenies with much larger datasets.

In the second largest insect order Lepidoptera, there were 172 mitogenomes representing 113 species on NCBI (as accessed on May 14, 2014). However, 44 of the 172 submitted mitogenomes were from the genus *Bombyx*[20–22], 53 of the 113 species were from butterflies (Papilionoidea), and only ten of 43 currently recognised superfamilies have been covered [23–27]. For inferring molecular phylogeny, mitochondrial sequences have been questioned on the basis that they are too saturated to distinguish deep-level relationships [28, 29]. Though some studies point out that other mitochondrial genes have more relevant information than the most commonly used *cox1* or *rrnL* genes [9, 27, 30], researchers have instead focused on using nuclear genes to infer phylogenies. To evaluate sufficiently the utility of mitogenomes in inferring lepidopteran relationships at the level of family and below, here we focus on the well-known group of brush-footed butterflies, Nymphalidae.

The family Nymphalidae was chosen as our primary focus, firstly because this group represents the most diverse butterfly family with 12 subfamilies, 559 genera and 6,152 species, that is one third of all butterfly species [31–34]. Secondly, Nymphalidae is also arguably the most utilised lepidopteran family in biological studies as they are distributed in various habitats worldwide and include many model species for ecological, conservation, evolutionary and developmental studies [35–37]. Thirdly, there is a good existing framework against which to judge our results, because the backbone relationships of Nymphalidae have been inferred based on dense sampling of both morphological characters and DNA sequences [31, 38]. Nymphalidae phylogeny is far from fully resolved though, because although groups at subfamily and tribal levels appear to be monophyletic, some nodal support at these levels remains weak with no topological consensus. In particular, the branching pattern at deep nodes remains unresolved: both subfamilies Libytheinae and Danainae have been placed as sister to the rest of the family, but without strong nodal support (ML bootstrap ≤ 70, MP bootstrap < 48) [31]. Also in the subfamily case study we focus on here, the grouping of the tribes (Adoliadini and Limenitidini) in the Limenitidinae has been inconclusive due to low support values (MP bootstrap =40, and ML bootstrap not significant) [31].

There is also a particular prior interest in the tribe Limenitidini, as these butterflies include models for the evolution of mimicry [39]. The mimetic wing patterns of *Limenitis* butterflies are considered to evolve multiple times and hybridised frequently [40, 41] and the wing patterns of these gliders have converged to an extraordinary degree for butterflies not widely thought to be Müllerian mimics with strong predator unpalatability [42]. The phylogenetic relationships and evolutionary patterns of the American Limenitidini butterflies have been analysed in particular detail [39, 40, 43]. By contrast, the phylogenetic relationships of Palearctic Limenitidini butterflies have been almost neglected. Even though some studies have used nuclear (*EF1*-*α*) and mitochondrial genes (*cox1* or *cob* gene) to investigate the relationships of East Asian Limenitidini [44, 45], results based on these genes are still unresolved and poorly supported. A further study even showed a polyphyletic relationship of the genus *Athyma*[46], part of which had a sister relationship with *Limenitis* butterflies [31]. Further effort is needed to clarify the relationship of this group and unravel the evolutionary basis of mimicry.

The molecular data deposited in public databases have taxonomic biases and annotation errors [47, 48]. However, they still enable the testing of various strategies of data partitioning and substitution models to improve the accuracy of phylogenetic inference [49, 50]. Branch lengths differ greatly when different substitution models are applied, potentially producing different topological and molecular dating estimates [51]. The mitogenome has long been considered as a single non-recombining locus because of maternal inheritance, storing substantial numbers of mutations heterogeneously distributed among genes [52, 53]. The lack of extensive taxon sampling across Nymphalidae, however, has impeded the evaluation of the effects of different partitioning methods on mitogenomic phylogenies. A denser coverage of nymphalid mitogenomes subjected to various partitioning methods is needed for accurate reconstruction of phylogenies in this group.

The aim of this study was to establish a highly supported phylogeny for the Nymphalidae using 70 mitogenomes obtained from both our pyrosequencing-derived mitogenomes (n = 30) and those previously deposited in GenBank (Additional file 1). The combined datasets were used to evaluate nodal supports using 12 different partitioning strategies (PSs) and two categories of substitution models. Finally, the phylogenies inferred from entire mitogenomes and single genes were compared to assess topological congruence.

## Results

### Sequence information

**Figure 1:**
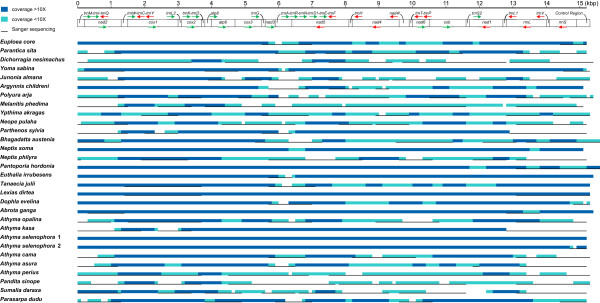
**An overview of 30 newly sequenced mitogenomes.** The sequencing regions derived from pyrosequence-assembled contigs, Sanger sequencing, and gaps are shown.

A total of 30 mitogenomic sequences (total length of 458,050 bp) were obtained from two Roche Junior runs (Additional file 1). After quality control treatments and sequence assembly, a total of 121,305 pyrosequencing reads were obtained (Additional file 2). The mean length of read was 403.6 bp with mean quality Q-score 28.2, indicating that the error rate of our sequences is near 0.001. Sequence coverage was not uniform (Figure 1, Additional file 2). Generally, highest coverage regions were located at both ends of the amplicons, whereas low coverage regions (<10X) were often found near the control region or in the tRNAs region between the *nad3* and *nad5* genes in cases where these regions contained polymeric stretches or tandem repeats (Figure 1, Additional file 3). The low sequence recovery for some species might have been caused by unequal DNA estimation. Details about the lengths sequenced via pyrosequencing and Sanger methods, overall sequence coverage, and gaps, are shown in Figure 1.

To verify sequence information, most gaps and low-coverage regions were checked via Sanger sequencing. This also allowed us to fill in many gaps. However, some ambiguous sites remained unresolved (all the ambiguous sites are listed in Additional file 3). Six major undetermined regions are listed here: (1) for *Athyma perius*, 79 ambiguous sites in the control region; (2) for *Dichorragia nesimachus*, an undetermined 33-bp length within the *rrnL* gene; (3) for *Euthalia irrubesens*, a 52-bp region within a TA-repeat noncoding region between the *trnE* and *trnF* gene; (4) for *Neope pulaha*, a 16-bp region in the *nad6* gene; (5) for *Tanaecia julii*, a 25-bp region within a TA-repeat noncoding region between the *trnE* and *trnF* genes; and (6) for *Sumalia daraxa*, a 601-bp fragment containing part of 3’ end of the *nad1* gene and *trnS2* gene which failed to sequence. The ratio of these sites was below 0.02% of our total sequenced mitogenomes (Additional file 3). This proportion of missing data seems not to seriously affect the evaluation of phylogenetic utility.

The lengths of these new and published mitogenomes are quite conserved between 15-16 kb. *Neptis soma* has the shortest sequence length (15,130 bp), whereas *Papilio maraho* has the longest (16,094 bp) (Additional file 1). Although most length variation was found in the control region, some additional lengths were also observed among these 30 mitogenomes. There was a 150-bp additional stretch found between the *nad2* and *trnW* genes (for *Bhagadatta austenia*), a region including (TA)^11-64^ repeats that are found between the *trnE* and *trnF* genes (for *Bhagadatta austenia*, *Dichorragia nesimachus*, *Dophla evelina*, *Euthalia irrubesens*, *Junonia almana*, *Parthenos sylvia*, *Polyura arja* and *Tanaecia julii*), a 41-bp length found between the *nad6* and *cob* genes (for *Yoma sabina*), a 34-bp fragment found between the *trnL1* and *rrnL* genes (for *Polyura arja*), and a 59-bp fragment observed in the centre of the *rrnL* gene (for *Bhagadatta austenia*), and finally a 37-bp fragment found in the *rrnS* gene of *Ypthima akragas*.

The gene order and orientation were the same in all 70 assembled lepidopteran mitogenomes. This alignment suggests that the arrangement of three tRNAs (CR-M-I-Q-*nad2*) between the control region (CR) and the *nad2* gene can be inferred as a derived character relative to the insect ground plan, CR-I-Q-M-*nad2*[54]. The A + T compositional ratio of the obtained mitogenomes ranged from 77.8-82.7% (Additional file 4), which is within the range of most insect mitogenomes [55].

The 37-gene aligned matrix contained 15,495 bp, of which 11,340 bp was derived from the 13 PCGs, 2,528 bp from the two rRNA genes, and 1,627 bp from the 22 tRNA genes. A total of 6,920 polymorphic sites, 5,714 of them parsimony-informative, were identified (Additional file 5). Removing the four outgroups resulted in 6,710 variable sites, 5,472 of them parsimony-informative. Both the numbers of variable and parsimony-informative sites were lower in the 15-gene dataset than those in the 37-gene dataset (Additional file 5). However, the ratio of informative sites to polymorphic sites was slightly higher than that in the 37-gene dataset. A similar pattern was also found in the 13-gene dataset.

**Figure 2:**
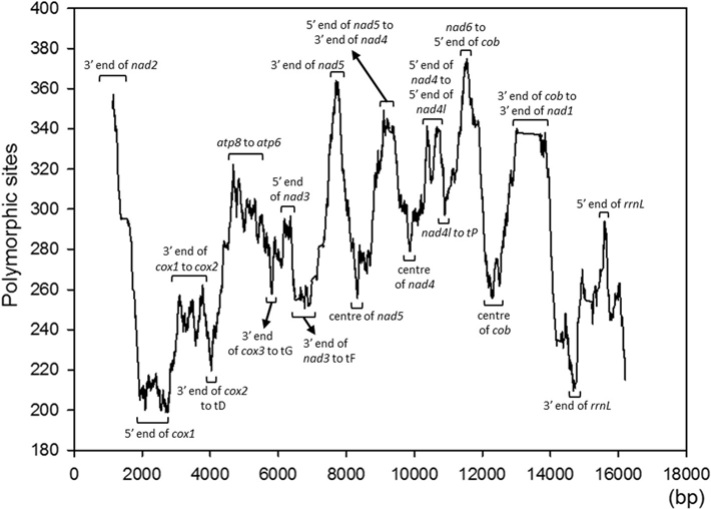
**A sliding window of 70 sampled mitogenomes (alignment length: 18,646 bp).** The sliding window was derived using DnaSP v5 software. The window width was set to 500 bp and the step size was set to 2 bp (excluding gaps and missing data).

**Figure 3:**
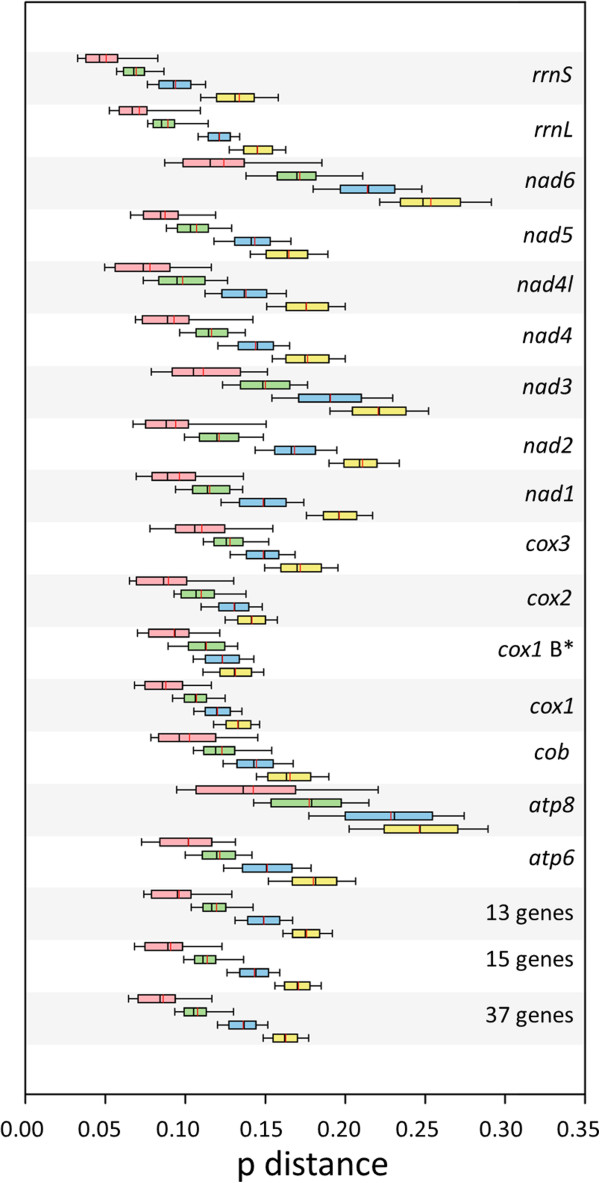
**Genetic distance variation among different datasets.** The red line is the mean and the *cox1* B* is the barcode region. Genus level in pink; tribal level in green; subfamily level in blue; family level in yellow.

Sequence variable sites were not homogeneously distributed across the gene (Figure 2). Most polymorphic sites were found towards the 5’ or 3’ ends of genes, while relatively conserved regions were found towards the centre of PCGs. Two genes (*nad6* and the 3’ end of *nad5*) had the most variable regions, whereas the 5’ end of *cox1* (barcode region) and *rrnL* were the most conserved (Figure 3).

### Phylogenies

**Figure 4:**
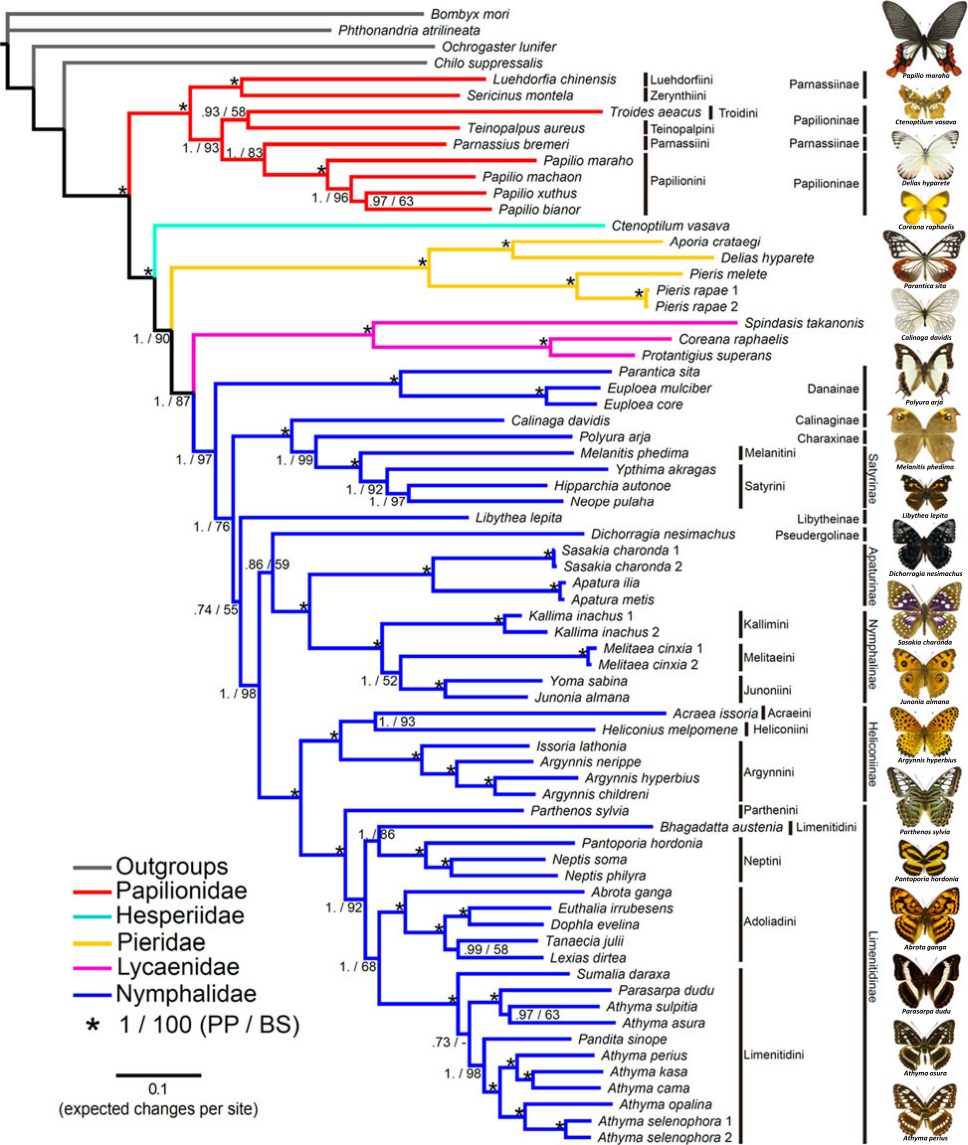
**Mitogenomic phylogeny of sampled butterflies.** The BI topology was based on the PS2 partitioning strategy and the best-fit model setting. The values at each node are posterior probability (PP) and ML bootstrap (BS). The label “*” means PP =1 and BS = 100.

**Figure 5:**
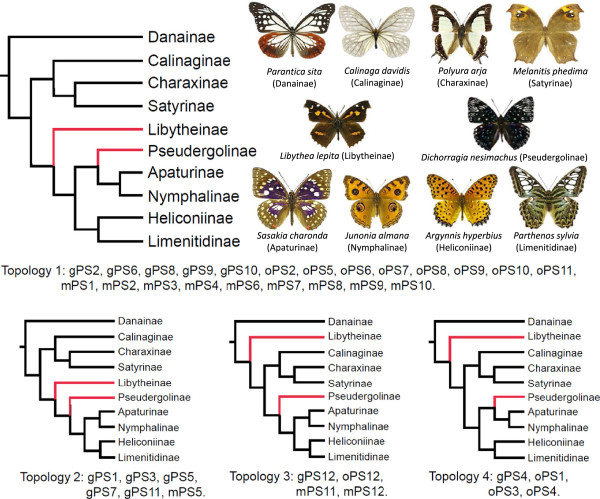
**Four mitogenomic topologies of Nymphalidae subfamilies.** The four topologies were summarised from 36 datasets based on the BI and ML methods. The symbols "gPS1 to gPS12" mean BI topologies inferred with the GTR + G model, whereas “oPS1 to oPS12” means BI topologies inferred with the best-fit model. The symbols "mPS1 to mPS12" mean topologies inferred by the ML method with the GTR + G model.

Phylogenetic relationships inferred by Bayesian inference (BI) and maximum likelihood (ML) methods were congruent and strongly supported (>0.95 posterior probability and ML bootstrap >85% for most nodes), across each PS and substitution models for each of the three datasets (Figure 4; for more supporting information, see Additional files 6 and 7 for the BI trees, Additional file 8 for the ML trees, and Additional file 9 for 12 different PSs). Concerning family-level relationships, the topology is concordant with a recent study [32], in which the family Hesperiidae (along with Hedylidae) is subsumed within Papilionoidea, and the family Papilionidae is the sister clade of the remaining butterflies. At the subfamily level within Nymphalidae, ten sampled subfamilies were also well supported. Calinaginae and Charaxinae grouped with Satyrinae (hereafter referred as “satyroid” group). Apaturinae and Nymphalinae was a sister group and most closely related to the clade comprising Heliconiinae and Limenitidinae. Danainae was the sister of the remaining nymphalid subfamilies. However, the phylogenetic positions of Pseudergolinae and Libytheinae were not stable among the 12 different PSs (Figure 5). Libytheinae was placed as the sister of all nymphalids except Danainae, or as sister to a clade composed of Pseudergolinae, Apaturinae, Nymphalinae, Heliconiinae, and Limenitidinae (Figure 5). Pseudergolinae showed a sister relationship to the clade that included Apaturinae, Nymphalidae, Heliconiinae, and Limenitidinae, but also showed a relationship to the clade composed of Apaturinae and Nymphalidae only.

Tribal-level relationships within Nymphalidae were well supported (Figure 4). According to our sampling focus on the subfamily Limenitidinae, four tribes were highly supported by different PSs (Figure 4; Additional files 6, 7 and 8). The tribe Parthenini was sister to a clade in which Neptini was sister to the group composed of Adoliadini + Limenitidini. Although the species *Bhagadatta austenia* has been classified within the tribe Limenitidini [56], our data showed this species always grouped with Neptini with high support. Within Limenitidini, a previous study indicated that the genus *Athyma* was paraphyletic [37]. Our analyses provided strong supports at nodes, indicating that *Athyma* is an artificial group.

Although our taxon sampling is limited, some well-supported relationships were also found in other subfamilies. Within the subfamily Heliconiinae, the tribe Argynnini was sister to the clade composed of Acraeini + Heliconiini [57]. Within the subfamily Nymphalinae, the three tribes Kallimini, Junoniini and Melitaeini formed a clade with strong support. Melitaeini, embedded within the subfamily Nymphalinae, in concordance with the result of Wahlberg et al. [31]; however, the ML bootstrap value is too low to support Kallimini as sister to the clade comprising Junoniini and Melitaeini.

**Figure 6:**
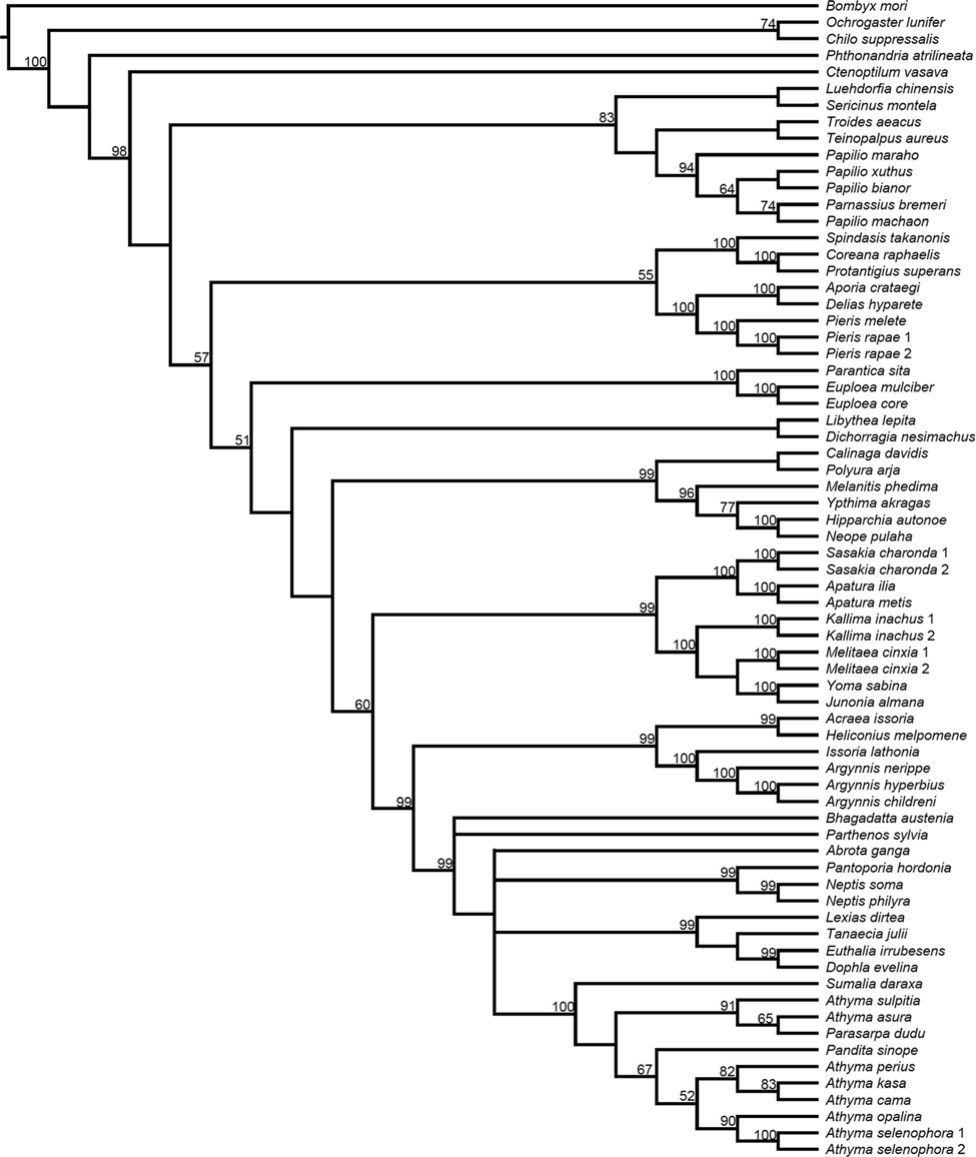
**Most parsimonious topology.** This topology was based on the 13-gene dataset using 50% majority rule. Two equally most parsimonious trees were found.

The topologies inferred by parsimony (MP) were different from the BI and ML trees at family and subfamily levels (Figure 6 and Additional file 10). All three datasets showed the same higher-level (family, subfamily, and tribal level) relationships, however, there was some incongruence at species level. A total of 19 most parsimonious trees were found for the 37-gene dataset, seven for the 15-gene dataset, and two for the 13-gene dataset. Two major inconsistences were found when compared with the likelihood topologies at family level (1) Hesperiidae was placed as sister to four sampled butterfly families, and (2) a sister group relationship was found between Lycaenidae and Pieridae. The latter relationship was also found in the most recent analysis using MP [32], but these morphologically implausible family-level relationships had lower bootstrap values in MP analyses than in the ML and BI analyses. At subfamily level within Nymphalidae, Danainae was also found to be sister to the other nymphalids. Other subfamilies had similar relationships to those inferred by ML and BI, except for an unexpected result that Libytheinae and Pseudergolinae grouped together and were placed sister to other nymphalids.

A total 15 individual single-gene phylogenies were inferred and none recovered the topologies generated using the whole mitogenomic alignment (Additional file 11).

#### Bayes factors

Bayes factors for the 12 PSs are shown in Table 1. Our results show that partitioned datasets are strongly preferred over non-partitioned ones (PS1, PS5, and PS9), and more partitioning is preferred over less. One exception is that codon partitioning (PS2, PS6, and PS10) performed better than gene partitioning (PS3, PS7, and PS11). Comparing the marginal likelihood values by two different model settings (optimal substitution models and GTR + G model), most of the Bayes factor results showed that the datasets based on the best-fit models had better performance than those based on the GTR + G model. Only the most highly partitioned strategy (PS4, PS8, and PS12, separately) was preferred over the same strategies using the best-fit substitution model.

**Table 1:** **Bayes factor comparisons between models**

**(A)**							
	**gPS1**	**oPS1**	**gPS2**	**oPS2**	**gPS3**	**oPS3**	**gPS4**
gPS1							
oPS1	13.86						
gPS2	17.59	17.25					
oPS2	17.80	17.49	13.17				
gPS3	17.27	16.87	-13.77	-14.87			
oPS3	17.59	17.25	-3.12	-13.18	13.76		
gPS4	18.88	18.71	17.39	17.13	17.69	17.39	
oPS4	18.68	18.50	16.96	16.63	17.33	16.96	-14.09
**(B)**							
	**gPS5**	**oPS5**	**gPS6**	**oPS6**	**gPS7**	**oPS7**	**gPS8**
gPS5							
oPS5	13.49						
gPS6	17.59	17.31					
oPS6	17.75	17.50	12.73				
gPS7	17.03	16.66	-14.74	-15.36			
oPS7	17.37	17.06	-13.05	-14.28	13.61		
gPS8	18.78	18.63	17.18	16.95	17.69	17.41	
oPS8	18.56	18.40	16.66	16.35	17.30	16.96	-14.22
**(C)**							
	**gPS9**	**oPS9**	**gPS10**	**oPS10**	**gPS11**	**oPS11**	**gPS12**
gPS9							
oPS9	13.15						
gPS10	17.28	17.01					
oPS10	17.44	17.20	12.38				
gPS11	16.61	16.22	-14.76	-15.29			
oPS11	16.95	16.63	-13.51	-14.41	13.23		
gPS12	18.60	18.47	17.15	16.96	17.68	17.45	
oPS12	18.47	18.32	16.86	16.64	17.46	17.20	-13.17

Entries are twice the log of the Bayes factor in the comparison between models M_0_ and M_1_ (2ln B_10_). “gPS1 to gPS12” are the datasets based on the GTR + G model; “oPS1 to oPS12” are based on the optimal (best-fit) model. (A): the 37-gene dataset; (B): the 15-gene dataset; (C): the 13-gene dataset.

### The effect of data size and partitioning strategies on tree uncertainty

The summary of credible sets of Bayesian trees, treated by different PSs and substitution models, is presented in Table 2. The results show that the number of trees increases with increasing data partitioning among the 12 PSs. The number of credible trees produced from 15 single-gene datasets is two orders of magnitude greater than that from 12 PS datasets, indicating that 12 PS datasets had sufficient information to decrease tree uncertainty.

**Table 2:** **The effect of used datasets and models on tree uncertainty**

Datasets	Partitions	Sum of free parameters of substitution model	Harmonic mean	Credible sets of trees			Ngens (million)	Tree length	
				Total	99%	95%		Mean	SD
gPS1 (GTR + G)	1	9	-283566.6	315	61	21	10	13.45	1.20
oPS1 (best-fit)	1	10	-282542.4	321	76	26	10	13.57	1.13
gPS2 (GTR + G)	4	39	-276972.1	297	105	48	5	16.24	0.29
oPS2 (best-fit)	4	42	-276249.7	250	84	32	5	16.19	0.31
gPS3 (GTR + G)	37	369	-277947.8	329	104	37	10	16.32	0.32
oPS3 (best-fit)	37	377	-276976.9	464	196	90	10	15.33	0.37
gPS4 (GTR + G)	63	629	-271013.3	1324	651	311	20	27.06	0.71
oPS4 (best-fit)	63	594	-272160.4	879	396	168	20	19.36	0.54
gPS5 (GTR + G)	1	9	-265711.0	211	40	13	10	13.56	1.16
oPS5 (best-fit)	1	10	-264861.3	197	43	13	10	13.52	1.15
gPS6 (GTR + G)	4	39	-259125.8	212	54	21	5	16.97	0.33
oPS6 (best-fit)	4	42	-258543.5	158	35	14	5	16.98	0.33
gPS7 (GTR + G)	15	149	-260710.7	212	43	20	10	17.42	0.25
oPS7 (best-fit)	15	160	-259806.2	319	86	31	10	16.13	0.37
gPS8 (GTR + G)	41	409	-253761.0	360	107	37	15	29.20	0.84
oPS8 (best-fit)	41	377	-254988.1	417	132	41	10	20.58	0.61
gPS9 (GTR + G)	1	9	-230862.5	187	36	11	10	13.66	1.12
oPS9 (best-fit)	1	10	-230146.3	368	104	42	10	13.63	1.11
gPS10 (GTR + G)	3	29	-225214.1	145	37	10	5	18.28	0.37
oPS10 (best-fit)	3	31	-224727.3	133	32	10	5	18.70	0.38
gPS11 (GTR + G)	13	129	-226815.1	165	33	9	10	19.24	0.26
oPS11 (best-fit)	13	138	-226070.4	311	84	30	10	17.38	0.49
gPS12 (GTR + G)	39	351	-219907.9	544	215	102	15	32.45	0.99
oPS12 (best-fit)	39	329	-220632.0	616	209	79	15	24.82	0.77
*atp6* (best-fit)	1	10	-14049.63	73984	73234	70234	5	13.49	1.16
*atp8* (best-fit)	1	6	-4499.38	74940	74190	71190	5	13.55	1.17
*cob* (best-fit)	1	10	-22793.23	71732	70982	67982	5	13.55	1.15
*cox1* (best-fit)	1	10	-25561.25	71273	70523	67523	5	13.49	1.16
*cox2* (best-fit)	1	10	-11819.12	74157	73407	70407	5	13.56	1.16
*cox3* (best-fit)	1	10	-16508.88	71202	70452	67452	5	13.56	1.17
*nad1* (best-fit)	1	10	-19188.52	71012	70262	67262	5	13.52	1.16
*nad2* (best-fit)	1	10	-21626.06	72082	71332	68332	5	13.53	1.18
*nad3* (best-fit)	1	10	-8158.40	74083	73333	70333	5	13.53	1.16
*nad4* (best-fit)	1	10	-26926.49	51974	51224	48224	5	13.57	1.13
*nad4l* (best-fit)	1	10	-5346.70	74887	74137	71137	5	13.50	1.16
*nad5* (best-fit)	1	10	-33532.70	61947	61197	58197	5	13.53	1.16
*nad6* (best-fit)	1	10	-13900.85	73991	73241	70241	5	13.51	1.16
*rrnL* (best-fit)	1	10	-22664.69	72493	71743	68743	5	13.51	1.16
*rrnS* (best-fit)	1	10	-10575.04	74137	73387	70387	5	13.55	1.15

Topological uncertainty increases with model complexity (number of parameters), and tree length uncertainty is positively correlated with increased uncertainty. The best-fit models of datasets are listed in the Additional file 13.

## Discussion

### Mitogenomes in butterfly systematics

Our results show that mitochondrial gene length and order are conserved among 70 sampled lepidopteran mitogenomes, and that the 37-gene aligned matrix includes over 36% parsimony-informative sites. Based on this genetic variation, our BI and ML analyses all show strong support for relationships at different hierarchical levels (Figure 4, and Additional files 6, 7 and 8). This strong congruence is not only supported with the most recent phylogenetic studies of butterflies at family and subfamily-level [32], but is also concordant with other mitogenomic phylogenies [23, 24]. Though taxon sampling is limited across butterfly families in our mitogenomic analyses, this study demonstrates that the entire mitogenome constitutes a particularly efficient marker for studying the phylogeny of butterflies as well as other groups of insects [11, 16–18, 58].

Encouragingly, our mitogenomic phylogenies provide an insight into deep-level relationships of Nymphalidae. At subfamily level, Danainae is placed as sister to the remaining nymphalids (Figure 4). However, this outcome is both inconsistent with a morphologically-based study [38] and with the most comprehensive taxa-sampling study of Nymphalidae [31], in which Libytheinae emerges as sister to the other nymphalids. It should be noticed that the new position of Libytheinae (Figure 5) substantially alters the interpretation of the deep evolutionary history of Nymphalidae. Libytheinae has distinct morphologies compared to other nymphalids (a long labial palpus and a fully developed female foreleg that was thought to be reduced in all other nymphalids) [59], and it has well-preserved late Eocene fossils, important for calibrating the dating scheme of butterflies [60]. As well as being supported in previous mitogenomic studies [23, 24], this mitogenome-suggested position for both Danainae and Libytheinae may reflect important rather than conflicting signal in the mitogenomic data and merits further attention including recalibration as necessary.

For the “satyroid” assemblage of subfamilies, mitogenomic phylogenies provide strong support within these three subfamily-level topologies across 12 PSs (Figure 4, Additional files 6, 7 and 8). These results indicate that the mitogenome might be a potential marker to re-investigate a long-standing phylogenetic question within the subfamily Satyrinae [34, 38, 61], for which deep-level relationships, across the clades of Elymniini, Zetherini, Satyrini, Dirini, Melanitini, Haeterini, and Morphini, remain controversial. Though our sampling is limited for the large subfamily Satyrinae, the tribe Satyrini was monophyletic with respect to Melanitini with strong support, while Ypthimina (*Ypthima akragas*) was sister to the clade composed of Satyrina (*Hipparchia autonoe*) and Lethina (*Neope pulaha*).

As for relationships within the subfamily Limenitidinae, the four tribes were all monophyletic and well-supported (Figure 4). The tribal relationships are clearly resolved by the BI method, though ML bootstrap shows lower support for the clade comprising Adoliadini + Limenitidini, indicating that taxon sampling might still not be sufficient. At least, we can confirm that *Bhagadatta* is a Neptini and within the Limenitidini, the genus *Athyma* is polyphyletic and in need of taxonomic revision. We also note that further work is needed according to these phylogenetic results to illuminate mimetic and evolutionary processes of Asian Limenitidini butterflies in detail.

Some conflicting relationships are found when comparing our results to phylogenetic studies of Papilionidae [62, 63]. The subfamilies of Parnassiinae and Papilioninae are sister groups, and four Papilioninae tribes are clearly recognised [62]. However, our results show *Parnassius bremeri* did not group with other Parnassiinae members; instead, this species grouped with the tribe Papilionini (Figure 4). We checked its identification via NCBI and BOLD databases (using the *cox1* gene), and this taxon clusters within *Parnassius phoebe*, showing consistency with *Parnassius*. This lack of nesting within Parnassiinae merits further examination, but we note that the tribal relationships of Parnassiinae are not strongly supported so far [63]. Moreover, our topology suggests that long-branch attraction has occurred in our mitogenomic phylogenies. In Papilionidae, the tribe Teinopalpini is regarded as the sister group of Papilionini + Troidini [62], but our result unexpectedly showed a sister relationship of Teinopalpini + Troidini as in other mitogenomic studies [23, 24]. These conflicting relationships might be caused by inadequate taxon sampling in our study, but we strongly suspect that the public papilionid mitogenomes in particular require detailed validation and/or resequencing. In Nymphalidae, the subfamily Pseudergolinae had low nodal support in our inferred topologies, yet its position was highly supported by Wahlberg et al. [31] (as also in our topology 4 of Figure 5), indicating that greater taxon sampling is important to resolve such equivocal relationships [64]. Further studies should add the subfamilies Biblidinae and Cyrestinae to clarify this point.

### Utility of mitogenomic data in Lepidoptera

Higher level phylogenetic relationships of Lepidoptera have been studied and revised based largely on increasing numbers of nuclear genes, recently using the RNAseq approach [65, 66]. However, some relationships are still conflicting due to limited taxon sampling (for Lepidoptera, particularly limited at superfamily level) or missing sequences. Similar conflicts are found when the studies focus on a subset of taxa [67, 68]. We agree that nuclear genes are useful for inferring deep-level lepidopteran relationships, but it is a technological and financial challenge to obtain a huge dataset composed of several nuclear genes from many species and a serious computing challenge to analyse them. Instead, our study has demonstrated that it is much easier to obtain a large number of mitogenomes via NGS technologies, and the need for filling with Sanger sequencing will further reduce as technologies and coverage improves. To infer molecular phylogenies in Lepidoptera, we suggest using mitogenomic sequences to infer phylogeny as a first step, following by more nuclear genes as a second step to obtain highly stable relationships based on unlinked gene histories.

Mitochondrial genes have a mutation rate an order of magnitude faster than nuclear DNA [69], and these sequences are thus susceptible to phylogenetic noise and long-branch attraction in insects [29, 70, 71]. Even inference of deep-level relationships based on one or two mitochondrial genes shows much inferior results than that based on a handful of nuclear genes. This is why mitochondrial sequences have not historically been favoured in phylogenetic reconstruction. This viewpoint is misconceived. Obviously, phylogenetic results inferred from one or two genes cannot represent the phylogenetic utility of informative SNPs across whole mitogenomes. Comparing topologies inferred from the 13, 15, and 37-gene datasets, phylogenies inferred from individual genes do not even recover the same subfamily and tribal-level relationships (Additional file 11). It is apparent that single mitochondrial genes simply do not have enough information to infer relationships at this level, even though they include slow-evolving sites whose character changes support some deeper nodes. For investigating deep-level relationships, Cameron [9] points out that gene annotation of insect mitogenomes also provides useful information to reveal the evolution of insect mitogenomes. In Lepidoptera, the gene order of *trnM*-*trnI*-*trnQ* is considered a derived character, and only one study has addressed the ancestral gene arrangement found in Hepialidae [72], while one study has found that the *atp6* and *atp8* genes are absent between the *cox2* and *cox3* genes due to gene rearrangement in the species, *Rohana parisatis* (Nymphalidae, Apaturinae) [73]. Our 70 sampled mitogenomes all reveal the derived gene order, indicating that this genome rearrangement is conserved in most Lepidoptera. Any new gene annotations observed in Lepidoptera will be diagnostic characters for phylogenetic inference.

### The effects of model selection and data partitioning in model-based analyses

The current approaches to reconstructing phylogenies emphasise the selection of proper substitution models and partitioning methods [49, 51, 74]. Although gene or codon partitioning is the appropriate method for analysing protein coding genes [74], the best-fit partitioning method is still under debate. It is evident from our sliding-window result (Figure 2) that SNPs were not uniformly distributed by gene region. Nevertheless, our results suggest that different partitioning strategies should always be adopted to evaluate the effect of partitioning on phylogenetic topology. In our case, the strategy with the most intense partitioning (PS4, PS8, and PS12) is favoured by the Bayes factors, even though all the topologies were independent of datasets with different lengths, partitioning schemes and substitution models (Figure 5). We also note that more free parameters (more complex models) would yield greater topological uncertainty (Table 2), a pattern consistent with other studies [49, 50].

Tree length is the product of substitution rate and time [51] but can be significantly affected by the assumptions of model selection [75]. In our case, the Bayes factors showed that phylogenies based on the best-fit model (oPS1-12) have better performance (Table 1), and these results confirm that although the mitochondrion evolves as a single gene because of maternal inheritance, dataset partitioning and model selecting are both important treatments to evaluate the phylogeny.

### NGS and Sanger sequencing methods for mitogenomes

For the purpose of obtaining large numbers of long sequences, the NGS method has clear economic advantages over Sanger sequencing [76]. The greatest benefit of NGS is to obtain massive quantities of sequence without primer design. Although the NGS method has error rates of 0.01-1% [41], high sequence coverage largely compensates for base errors. NGS methods are also very effective when target mtDNAs are rare and highly degenerated [77, 78]. As shown by recent advanced studies [17, 19] and our work, the 454-pyrosequencing method using less than a quarter of a 454 Titanium run (around 0.2 million reads) can recover over 85% target regions for a goal of obtaining near 30 mitogenomic sequences (Additional file 2). In our analyses, regions which cannot be pyrosequenced are often concentrated near the control region or in noncoding regions between the *cox3* and *nad5* genes (Figure 1). These gaps can easily be compensated by Sanger sequencing. Moreover, we emphasise that using the unique-tag method [79] multiplexing reads can more easily be divided to avoid chimaeric mitogenomes and to accelerate bioinformatic processing. Similarly, Timmermans et al. [17] report an efficient option of combining high sequence coverage by NGS with Sanger sequences which are used to bait the pyrosequencing-contigs.

In contrast to the NGS method, Sanger sequencing is more economic when the main purpose is to sequence one or a small set of mitogenomes due to their limited length (~16 kb). For example, our designed primers were largely based on conserved regions in 66 butterfly mitogenomes (Additional file 12). These primers could be used to sequence mitogenomes from other species of nymphalids. The data processing for Sanger-derived sequences requires less training in bioinformatics. Overall, each sequencing technology has its own advantages and is mutually complementary for maximising the efficiency of high quality sequence retrieval. We recommend first the application of NGS to recover a large proportion of the mitogenome, followed by Sanger sequencing as a complementary approach to obtain sequences for regions with low coverage, gaps or high ambiguity, or tag baits.

## Conclusions

This study aimed to obtain a large number of Lepidoptera mitogenomes simultaneously via NGS methods. The matrix of 30 newly obtained mitogenomes together with 40 others deposited in GenBank yields a well-supported phylogeny of the superfamily Papilionoidea and its subsets, suggesting that the entire mitogenome provides an excellent marker for studying the phylogeny of butterflies and other insect relationships. Our newly designed primers based on 66 nymphalids also provide a basis for sequencing additional butterfly mitogenomes by the Sanger method, while we anticipate that pyrosequencing will become prevalent for mitogenomic studies. We emphasise the need for good quality control or resequencing of existing public sequences, to avoid topological artefacts.

## Methods

### Sampling

A total of 36 butterfly mitogenomes deposited in GenBank (up to March 31, 2013) represent five of the seven recognised butterfly families and eight of the twelve subfamilies of Nymphalidae [23, 25, 30, 55, 80–103] (also see the references in Additional file 1). Among these subfamilies, five are represented by a single species. To investigate more comprehensively subfamily and tribal relationships within Nymphalidae, 30 additional nymphalids were selected. The combined datasets, representing ten of the twelve nymphalid subfamilies [31], comprise 20 species of Limenitidinae, six Heliconiinae, four Nymphalinae, four Satyrinae, three Apaturinae, three Danainae, one Libytheinae, one Pseudergolinae, one Calinaginae, and one Charaxinae (Additional file 1). For inferring detailed tribal relationships within Nymphalidae, we focused on the subfamily Limenitidinae, in which four tribes are recognised [31]. The tribe Parthenini is considered to be sister of the remaining limenitidine groups, while the relationships of the other three tribes (Neptini, Limenitini, and Adoliadini) remain controversial [31, 39]. Moreover, to evaluate the utility of mitogenomes at within-tribal level, we focused on the genus *Athyma*, of which the members show paraphyletic relationships with low nodal support [46]. Finally, we also sampled two specimens of *Athyma selenophora* from Taiwan and Hainan for examining subspecies-level identification. In addition, four moth mitogenomes were selected as outgroups on the basis of previously reported relationships [66].

### Molecular techniques

Genomic DNA was extracted from the thoracic muscle tissue or legs using the Purgene DNA Isolation kit (Gentra Systems, Minnesota, USA), following the manufacturer’s protocol. Precipitated DNAs were resuspended in 70 μL of sterile dH_2_O.

For amplifying long fragments of whole mitogenomes, TaKaRa LA Taq™ (Takara Bio Inc., Shiga, Japan) was used. Generally, two fragments were amplified, one of ~6.5 kb in length and covering the AT rich region, and the other of ~9.5 kb in length; the two fragments overlap at the *cox2* and *rrnL* gene regions. Primers were mostly adopted from Wu et al. [30]. PCR products were checked using 1-2% agarose gel with TAE buffer. Each product was purified using the UltraClean™ DNA Purification kit (MoBio Inc., Solana, CA, USA). The concentration of purified genomic DNA was measured using the Nanodrop ND-1000 (NanoDrop Technologies, Wilmington, DE, USA). The two long PCR products for each sample were mixed in equimolar concentrations and sheared into fragments of size 200-1000 bp. Before pyrosequencing, each sample was ligated with Roche adaptors and a unique species identifying tag. A total of 30 tagged-samples were mixed together and sequenced using the Roche 454 GS Junior System (Roche/454 Life Sciences, Branford, CT) at the Genomics BioSci & Tech Co. (Taipei, Taiwan).

A total of two dataset runs (one full and two half runs) were obtained in our study. The reads extracted from the raw data (sff-file) were binned in accordance with unique tag sequences using the Mothur 1.27 software [104]. The adaptors and unique tag regions were trimmed by Mothur, following the 454 SOP (http://www.mothur.org/wiki/454_SOP) to get fasta-format files of each sample. Sequences with a length of less than 50 bp and quality of less than Q20 were excluded. The dataset for each sample was assembled into contigs via the software MIRA 3 [105], using the default setting for the 454 platform. Contigs for each sample were checked and edited using Sequencher 4.8 (GeneCode, Boston, USA). The definition of possible gene regions, gene annotation, and PCG codon position were determined against published references of *Euploea mulciber*, *Argynnis hyperbius*, *Athyma sulpitia*, *Kallima inachus*, and *Hipparchia autonoe* (Additional file 1) by using Sequencher and alignment by eye which was straightforward at codon level. These results were also double-checked via the MITOS pipeline [106]. Gaps and low coverage regions (<10X) were re-sequenced and checked using Sanger sequencing on an ABI 3730 (Applied Biosystems, Foster City, CA, USA). Newly designed primers for Sanger sequencing are listed in Additional file 12. Although most sequences could be obtained and verified by pyrosequencing and Sanger sequencing, a few regions with polymers or multiple tandem repeats failed to sequence correctly. These missing or ambiguous sites were further designated as IUPAC codes (Additional file 3).

For phylogenetic analyses, the 37 mitochondrial genes were separately aligned by MUSCLE in MEGA5 [107]. PCGs were aligned according to amino sequence similarity, whereas RNA genes were directly aligned according to sequence similarity using default settings. All genes were concatenated using Microsoft Excel, and the datasets were exported as fasta-format files. General sequence information was analysed using both DnaSP v5 [108] and MEGA 5 software. All sequenced mitogenomes were submitted to GenBank (accession numbers KF590524-KF590553, also listed in Additional file 1), whereas pyrosequencing rawdata and individual barcode information were both submitted to the Sequence Read Archive under the study number of SRP041730.

### Data partitioning and model selection

In order to evaluate the effect of data partitioning and incorporation of RNAs on phylogeny, three datasets referred as the 37-gene dataset (13 PCGs plus two rRNAs and 22 tRNAs), the 15-gene dataset (13 PCGs plus two rRNAs), and the 13-gene dataset (13 PCGs only) were constructed. The three datasets were further partitioned by 12 strategies considering gene region and codon position (Additional file 9). For the 37-gene dataset, the partitioning strategies (PSs) were set as (1) no partition (the combined 37 genes), (2) four partitions with three for the codon position of the 13 PCGs and one for two rRNAs and 22 tRNAs combined, (3) 37 partitions, for each gene, and (4) 63 partitions, partitioning by both gene and codon position of the PCGs. For the 15-gene dataset, the PSs were set as (5) no partitions, combining the 13 PCGs and the two rRNAs, (6) four partitions, including three for the codon position of the PCGs and one for two rRNAs, (7) 15 gene partitions, and (8) 41 partitions, partitioning by both gene and codon position of the PCGs. The PSs of the 13-gene dataset was set as (9) no partition (the combined 13 PCGs), (10) three codon-based partitions, (11) 13 PCGs each as a single gene partition, and (12) 39 partitions based each gene and each codon position. The optimal substitution model of each partition (oPS) was determined by jModelTest 2 [109], using the corrected Akaike information criterion (AICc) (Additional file 13).

### Phylogeny

Three phylogenetic methods, Maximum Parsimony (MP), Bayesian inference (BI) and Maximum Likelihood (ML) were used to compare topologies on our datasets. MP was carried out using the TNT 1.1 [110], BI was conducted using the MrBayes 3.2.1 [111], and ML was performed in the RAxML Pthreads-based SSE3 version 7.4.2 [112, 113], with 16 precursors on a Linux system. For the BI analysis, two model settings (GTR + G model and best-fit models) were carried out on the 12 PSs (Additional file 13). First, partitioning datasets were constructed using the best-fit models. Some models (TVM, TIM1, TIM2, TIM3, TPM3, and TrN) that could not be directly used in MrBayes were replaced by the nearest over-parameterised model following a previous study [114]. Second, the model GTR + G was taken following the previous study [31], because the gamma shape (parameter G) is highly correlated with invariable sites (parameter I) [115]. We set all the partitions to the GTR + G model to compare the topologies with the best-fit model setting. The analyses of each dataset were performed with eight chains (seven heated and one cold) and run for five million generations. Every 100 generations were sampled as a consensus tree. The log-likelihood scores were plotted against generation time to determine whether stationarity was reached. Stationarity of Bayesian phylogenies was further assessed using the “sump” option to get the effective sample size (ESS) of parameters. If the ESS value was below 100, the number of generations was increased to 10-20 million. If stationarity was achieved, the first 25% of sampled trees were discarded and the remaining trees were used to represent the posterior probability (Table 2).

ML and MP methods were compared with the results of the BI method. For ML analyses, datasets obtained from each PS were processed with the model of GTRGAMMA, and all model parameters were estimated and joined to the branch length optimisation for the best ML tree. Node stability was evaluated using 1000 bootstrap replicates with 10 additional ML searches of each replicate to improve bootstrapping performance [112]. For MP analyses, three different length datasets (37-gene, 15-gene, and 13-gene) were run. Initially, the MP topologies were searched with 1000 random addition replicates and a MaxTrees of 10000. TNT searches were executed using Tree Ratchet, Tree Drifting, and Tree Fusing methods [116]. Nodal support was evaluated using 1000 bootstrap replicates. All three datasets were analysed using the same MP settings.

### The performance of one mitochondrial gene in phylogenetic reconstruction

To investigate the performance of each mitochondrial gene in reconstructing the phylogeny, the 15 major genes (13 PCGs and two rRNAs, respectively) were analysed. Each gene was analysed by BI: eight chains, the best-fit model (Additional file 13) and run for five million generations, sampled every 100. When stationarity was reached, the first 25% sampled trees were discarded and the remaining trees were used to calculate the posterior probability.

### Bayes factors

To investigate which partitioning model is preferred, we calculated Bayes factors to compare pairs of likelihood models. The value, B_10_, is calculated as the ratio of the model likelihoods f (X | M_1_)/f (X | M_0_), where the symbol X represents a generating data set, M_1_ and M_0_ are two compared models, and f (X | M) stands for model likelihood. We calculated the Bayes factors via the marginal likelihood, which was estimated as the harmonic mean (HM) of the likelihood scores using MrBayes “sump” option. The comparison for two PSs was calculated as 2ln (B_10_) = 2[ln (HM_1_) – ln(HM_0_)], where HM_1_ and HM_0_ are two harmonic means of each posterior probabilities. Positive values of 2ln (B_10_) indicate a preference for the later strategy over the former. Interpretations of significance were applied following Kass and Raftery [117]. For Bayes factor between 0 and 2, no model is preferred; for values between 2 and 6, model 1 is favoured over model 0; for values between 6 and 10, model 1 is strongly favoured; for the value over 10, model 1 is very strongly preferred. We took values over 10 as significant [49].

## Electronic supplementary material

Additional file 1: Table S1: General details of sampled taxa. *uncompleted mitogenome. (XLSX 16 KB)

Additional file 2: Table S2: General information of pyrosequencing reads in 30 sequenced mitogenomes. (XLSX 14 KB)

Additional file 3: Table S3: A list for all ambiguous sites when sequencing 30 mitogenomes. (XLSX 13 KB)

Additional file 4: Table S4: DNA composition of nucleotide A and T among different datasets. Gaps and missing data were excluded from the analysis. (XLSX 19 KB)

Additional file 5: Table S5: Nucleotide A + T composition and polymorphic sites of different regions. ^1*^excluding sites with gaps and missing data for calculating nucleotide compositions, variable sites. ^2*^excluding taxa of *Papilio xuthus*, *Luehdorfia chinensis*, and *Sumalia daraxa* due to partial coverage of the *nad1* gene. ^3*^excluding taxa of *Papilio xuthus*, and *Luehdorfia chinensis* due to partial coverage of *nad2* gene. (XLSX 13 KB)

Additional file 6: Figures S1-S12: Bayesian trees based on PS1-12 and the GTR + G model. Values at nodes correspond to posterior probabilities. (PDF 2 MB)

Additional file 7: Figures S13-S24: Bayesian trees based on PS1-12 and the best-fit model. Values at nodes correspond to posterior probabilities. (PDF 2 MB)

Additional file 8: Figures S25-S36: The ML phylogeny based on PS1-12 and the GTR + G model. Numbers above branches denote bootstrap support. (PDF 2 MB)

Additional file 9: Figure S37: An overview of twelve partitioning strategies in the study. (PDF 325 KB)

Additional file 10: Figure S38-S39: Maximum-parsimony topology based on 15 and 37-gene datasets, respectively. The seven and 19 most parsimonious trees were summarised by 50% majority-rule in Figure S38, and Figure S39, respectively. Bootstrap values over 50% are shown above the branches. (PDF 334 KB)

Additional file 11: Figure S40: The Bayesian phylogeny for each gene. Grey: outgroups; red: Papilionidae; cyan: Hesperiidae; yellow: Pieridae; magenta: Lycaenidae; blue: Nymphalidae. (PDF 167 KB)

Additional file 12: Table S6: Additional primers used in the study. (XLSX 12 KB)

Additional file 13: Table S7: Substitution model of each partition dataset implemented in jModelTest 2. The best-fit model is judged by the Akaike Information Criterion using a corrected version for small samples (AICc). Some best-fit models which could not be set to MrBayes were instead fit using the nearest over-parameterised model listed in the used model. (XLSX 14 KB)

## Abbreviations

AICc: Corrected Akaike information criterion
*atp6*: ATP synthase subunit 6
*atp8*: ATP synthase subunit 8
B_10_: The value of Bayes factor between two models
BI: Bayesian inference
BIC: Bayesian information criterion
*cob*: Cytochrome b
*cox1*: Cytochrome oxidase subunit I
*cox2*: Cytochrome oxidase subunit II
*cox3*: Cytochrome oxidase subunit III
CR: Control region
GTR + G model: Generalised time-reversible model with gamma distribution
HM: Harmonic mean
Mbp: Million base pairs
ML: Maximum likelihood
MP: Maximum parsimony
*nad1*: NADH dehydrogenase subunit 1
*nad2*: NADH dehydrogenase subunit 2
*nad3*: NADH dehydrogenase subunit 3
*nad4*: NADH dehydrogenase subunit 4
*nad4l*: NADH dehydrogenase subunit 4 l
*nad5*: NADH dehydrogenase subunit 5
*nad6*: NADH dehydrogenase subunit 6
NGS: Next-generation sequencing
P: Polymorphic sites
PCGs: Protein-coding genes
PI: Parsimony-informative sites
PS: Partitioning strategy
rRNA: Ribosomal RNA
*rrnL*: Large subunit of mitochondrial ribosomal DNA
*rrnS*: Small subunit of mitochondrial ribosomal DNA
SNPs: Single-nucleotide polymorphisms
SD: Standard deviation
tRNA: Transfer RNA
*trnA*: tRNA-Alanine (Ala)
*trnC*: tRNA-Cysteine (Cys)
*trnD*: tRNA-Aspartic acid (Asp)
*trnE*: tRNA-Glutamic (Glu)
*tnrF*: tRNA-Phenylalanine (Phe)
*trnG*: tRNA-Glycine (Gly)
*trnH*: tRNA-Histidine (His)
*trnI*: tRNA-Isoleucine (Ile)
*trnK*: tRNA-Lysine (Lys)
*trnL1*: tRNA-Leucine (Leu1) between *nad1* and *rrnL* genes
*trnL2*: tRNA-Leucine (Leu2) between *cox1* and *cox2* genes
*trnM*: tRNA-Methionine (Met)
*trnN*: tRNA-Asparagine (Asn)
*trnP*: tRNA-Proline (Pro)
*trnQ*: tRNA-Glutamine (Gln)
*trnR*: tRNA-Arginine (Arg)
*trnS1*: tRNA-Serine (Ser1) between *trnN* and *trnE* genes
*trnS2*: tRNA-Serine (Ser2) between *cob* and *nad1* genes
*trnT*: tRNA-Threonine (Thr)
*trnV*: tRNA-Valine (Val)
*trnW*: tRNA-Tryptophan (Trp)
*trnY*: tRNA-Tyrosine (Tyr).

## References

[1] Gray MW, Burger G, Lang BF (1999). Mitochondrial evolution. Science.

[2] Gray MW (2012). Mitochondrial evolution. Cold Spring Harb Perspect Biol.

[3] Boore JL (1999). Animal mitochondrial genomes. Nucleic Acids Res.

[4] Taylor RW, Turnbull DM (2005). Mitochondrial DNA mutations in human disease. Nat Rev Genet.

[5] Hebert PD, Penton EH, Burns JM, Janzen DH, Hallwachs W (2004). Ten species in one: DNA barcoding reveals cryptic species in the neotropical skipper butterfly *Astraptes fulgerator*. Proc Natl Acad Sci U S A.

[6] Avise JC (2000). Phylogeography: The History and Formation of Species.

[7] Avise JC (2009). Phylogeography: retrospect and prospect. J Biogeogr.

[8] Wiley EO, Lieberman BS (2011). Phylogenetics: Theory and Practice of Phylogenetic Systematics.

[9] Cameron SL (2014). Insect mitochondrial genomics: Implications for evolution and phylogeny. Annu Rev Entomol.

[10] Miya M, Takeshima H, Endo H, Ishiguro NB, Inoue JG, Mukai T, Satoh TP, Yamaguchi M, Kawaguchi A, Mabuchi K (2003). Major patterns of higher teleostean phylogenies: a new perspective based on 100 complete mitochondrial DNA sequences. Mol Phylogenet Evol.

[11] Cameron SL, Lambkin CL, Barker SC, Whiting MF (2007). A mitochondrial genome phylogeny of Diptera: whole genome sequence data accurately resolve relationships over broad timescales with high precision. Syst Entomol.

[12] Vilstrup JT, Ho SY, Foote AD, Morin PA, Kreb D, Krutzen M, Parra GJ, Robertson KM, de Stephanis R, Verborgh P, Willerslev E, Orlando L, Gilbert MT (2011). Mitogenomic phylogenetic analyses of the Delphinidae with an emphasis on the Globicephalinae. BMC Evol Biol.

[13] Morin PA, Archer FI, Foote AD, Vilstrup J, Allen EE, Wade P, Durban J, Parsons K, Pitman R, Li L, Bouffard P, Abel Nielsen SC, Rasmussen M, Willerslev E, Gilbert MT, Harkins T (2010). Complete mitochondrial genome phylogeographic analysis of killer whales (*Orcinus orca*) indicates multiple species. Genome Res.

[14] Duchene S, Frey A, Alfaro-Núñez A, Dutton PH, Thomas P, Gilbert M, Morin PA (2012). Marine turtle mitogenome phylogenetics and evolution. Mol Phylogenet Evol.

[15] Finstermeier K, Zinner D, Brameier M, Meyer M, Kreuz E, Hofreiter M, Roos C (2013). A mitogenomic phylogeny of living primates. PLoS One.

[16] Song N, Liang AP, Bu CP (2012). A molecular phylogeny of Hemiptera inferred from mitochondrial genome sequences. PLoS One.

[17] Timmermans MJ, Dodsworth S, Culverwell CL, Bocak L, Ahrens D, Littlewood DT, Pons J, Vogler AP (2010). Why barcode? High-throughput multiplex sequencing of mitochondrial genomes for molecular systematics. Nucleic Acids Res.

[18] Haran J, Timmermans MJ, Vogler AP (2013). Mitogenome sequences stabilize the phylogenetics of weevils (Curculionoidea) and establish the monophyly of larval ectophagy. Mol Phylogenet Evol.

[19] Chan Y-C, Roos C, Inoue-Murayama M, Inoue E, Shih C-C, Pei KJ-C, Vigilant L (2010). Mitochondrial genome sequences effectively reveal the phylogeny of *Hylobates* gibbons. PLoS One.

[20] Hu X-L, Cao G-L, Xue R-Y, Zheng X-J, Zhang X, Duan H-R, Gong C-L (2010). The complete mitogenome and phylogenetic analysis of *Bombyx mandarina* strain Qingzhou. Mol Biol Rep.

[21] Li D, Guo Y, Shao H, Tellier LC, Wang J, Xiang Z, Xia Q (2010). Genetic diversity, molecular phylogeny and selection evidence of the silkworm mitochondria implicated by complete resequencing of 41 genomes. BMC Evol Biol.

[22] Yukuhiro K, Sezutsu H, Itoh M, Shimizu K, Banno Y (2002). Significant levels of sequence divergence and gene rearrangements have occurred between the mitochondrial genomes of the wild mulberry silkmoth, *Bombyx mandarina*, and its close relative, the domesticated silkmoth, *Bombyx mori*. Mol Biol Evol.

[23] Yang X, Xue D, Han H (2013). The complete mitochondrial genome of *Biston panterinaria* (Lepidoptera: Geometridae), with phylogenetic utility of mitochondrial genome in the Lepidoptera. Gene.

[24] Lu H-F, Su T-J, Luo A-R, Zhu C-D, Wu C-S (2013). Characterization of the complete mitochondrion genome of diurnal moth *Amata emma* (Butler) (Lepidoptera: Erebidae) and its phylogenetic implications. PLoS One.

[25] Yang L, Wei Z-J, Hong G-Y, Jiang S-T, Wen L-P (2009). The complete nucleotide sequence of the mitochondrial genome of *Phthonandria atrilineata* (Lepidoptera: Geometridae). Mol Biol Rep.

[26] van Nieukerken E, Kaila L, Kitching I, Kristensen N, Lees D, Minet J, Mitter C, Mutanen M, Regier J, Simonsen T, Wahlberg N, Yen SH, Zahiri R, Adamski D, Baixeras J, Bartsch D, Bengtsson BA (2011). Order Lepidoptera Linnaeus, 1758. Zootaxa.

[27] Cameron SL, Whiting MF (2008). The complete mitochondrial genome of the tobacco hornworm, *Manduca sexta*, (Insecta: Lepidoptera: Sphingidae), and an examination of mitochondrial gene variability within butterflies and moths. Gene.

[28] Zheng Y, Peng R, Kuro-o M, Zeng X (2011). Exploring patterns and extent of bias in estimating divergence time from mitochondrial DNA sequence data in a particular lineage: a case study of salamanders (Order Caudata). Mol Biol Evol.

[29] Talavera G, Vila R (2011). What is the phylogenetic signal limit from mitogenomes? The reconciliation between mitochondrial and nuclear data in the Insecta class phylogeny. BMC Evol Biol.

[30] Wu L-W, Lees DC, Yen S-H, Hsu Y-F (2010). The complete mitochondrial genome of the near-threatened swallowtail, *Agehana maraho* (Lepidoptera: Papilionidae): evaluating sequence variability and suitable markers for conservation genetic studies. Entomol News.

[31] Wahlberg N, Leneveu J, Kodandaramaiah U, Pena C, Nylin S, Freitas AV, Brower AV (2009). Nymphalid butterflies diversify following near demise at the Cretaceous/Tertiary boundary. Proc R Soc B.

[32] Heikkilä M, Kaila L, Mutanen M, Peña C, Wahlberg N (2012). Cretaceous origin and repeated tertiary diversification of the redefined butterflies. Proc R Soc B.

[33] Wheat CW, Wahlberg N (2013). Critiquing blind dating: the dangers of over-confident date estimates in comparative genomics. Trends Ecol Evol.

[34] Peña C, Wahlberg N, Weingartner E, Kodandaramaiah U, Nylin S, Freitas AV, Brower AV (2006). Higher level phylogeny of Satyrinae butterflies (Lepidoptera: Nymphalidae) based on DNA sequence data. Mol Phylogenet Evol.

[35] Ehrlich PR, Hanski I (2004). On the wings of checkerspots: a model system for population biology.

[36] Sheppard PM, Turner J, Brown K, Benson W, Singer M (1985). Genetics and the evolution of Muellerian mimicry in *Heliconius* butterflies. Philos Trans R Soc Lond B Biol Sci.

[37] Pollard E, Yates TJ (1993). Monitoring Butterflies for Ecology and Conservation: The British Butterfly Monitoring Scheme.

[38] Freitas AVL, Brown K (2004). Phylogeny of the Nymphalidae (Lepidoptera). Syst Biol.

[39] Willmott KR (2003). Cladistic analysis of the Neotropical butterfly genus *Adelpha* (Lepidoptera: Nymphalidae), with comments on the subtribal classification of Limenitidini. Syst Entomol.

[40] Mullen SP (2006). Wing pattern evolution and the origins of mimicry among North American admiral butterflies (Nymphalidae: *Limenitis*). Mol Phylogenet Evol.

[41] Savage WK, Mullen SP (2009). A single origin of Batesian mimicry among hybridizing populations of admiral butterflies (*Limenitis arthemis*) rejects an evolutionary reversion to the ancestral phenotype. Proc Biol Sci.

[42] Prudic KL, Khera S, Sólyom A, Timmermann BN (2007). Isolation, identification, and quantification of potential defensive compounds in the Viceroy butterfly and its larval host–plant, Carolina willow. J Chem Ecol.

[43] Mullen SP, Savage WK, Wahlberg N, Willmott KR (2011). Rapid diversification and not clade age explains high diversity in neotropical *Adelpha* butterflies. Proc R Soc B.

[44] Zhang M, Zhong Y, Cao T, Geng Y, Zhang Y, Jin K, Ren Z, Zhang R, Guo Y, Ma E (2008). Phylogenetic relationship and morphological evolution in the subfamily Limenitidinae (Lepidoptera: Nymphalidae). Proc Natl Acad Sci U S A.

[45] Wu D, Hao J, Zhu G, Chen N, Su C, Pan H, Zhang X (2007). Phylogenetic relationships of butterflies in the subfamily Limenitinae based on mitochondrial Cytochrome b gene sequences. Zool Res.

[46] Zhang M, Cao T-W, Zhong Y, Guo Y-P, Ma E-B (2011). Phylogeny of Limenitidinae butterflies (Lepidoptera: Nymphalidae) inferred from mitochondrial Cytochrome Oxidase I gene sequences. Agric Sci China.

[47] Vilgalys R (2003). Taxonomic misidentification in public DNA databases. New Phytol.

[48] Schnoes AM, Brown SD, Dodevski I, Babbitt PC (2009). Annotation error in public databases: misannotation of molecular function in enzyme superfamilies. PLoS Comput Biol.

[49] Brown JM, Lemmon AR (2007). The importance of data partitioning and the utility of Bayes factors in Bayesian phylogenetics. Syst Biol.

[50] Nylander JAA, Ronquist F, Huelsenbeck JP, Nieves-Aldrey J (2004). Bayesian phylogenetic analysis of combined data. Syst Biol.

[51] Marshall DC, Simon C, Buckley TR (2006). Accurate branch length estimation in partitioned Bayesian analyses requires accommodation of among-partition rate variation and attention to branch length priors. Syst Biol.

[52] Galtier N, Nabholz B, Glémin S, Hurst GDD (2009). Mitochondrial DNA as a marker of molecular diversity: a reappraisal. Mol Ecol.

[53] Wei S, Shi M, Sharkey M, van Achterberg C, Chen X (2010). Comparative mitogenomics of Braconidae (Insecta: Hymenoptera) and the phylogenetic utility of mitochondrial genomes with special reference to holometabolous insects. BMC Genomics.

[54] Taylor MF, McKechnie SW, Pierce N, Kreitman M (1993). The lepidopteran mitochondrial control region: structure and evolution. Mol Biol Evol.

[55] Salvato P, Simonato M, Battisti A, Negrisolo E (2008). The complete mitochondrial genome of the bag-shelter moth *Ochrogaster lunifer* (Lepidoptera, Notodontidae). BMC Genomics.

[56] Bridges CA (1988). Catalogue of Family-group and Genus-group names (Lepidoptera: Rhopalocera).

[57] Silva-Brandão KL, Wahlberg N, Francini RB, Azeredo-Espin AML, Brown KS, Paluch M, Lees DC, Freitas AV (2008). Phylogenetic relationships of butterflies of the tribe Acraeini (Lepidoptera, Nymphalidae, Heliconiinae) and the evolution of host plant use. Mol Phylogenet Evol.

[58] Li H, Liu H, Song F, Shi A, Zhou X, Cai W (2012). Comparative mitogenomic analysis of damsel bugs representing three tribes in the family Nabidae (Insecta: Hemiptera). PLoS One.

[59] Kawahara AY (2009). Phylogeny of snout butterflies (Lepidoptera: Nymphalidae: Libytheinae): combining evidence from the morphology of extant, fossil, and recently extinct taxa. Cladistics.

[60] Sohn J-C, Labandeira C, Davis D, Mitter C (2012). An annotated catalog of fossil and subfossil Lepidoptera (Insecta: Holometabola) of the world. Zootaxa.

[61] Miller LD (1968). The higher classification, phylogeny and zoogeography of the Satyridae (Lepidoptera). Mem Am Entomol Soc.

[62] Simonsen TJ, Zakharov EV, Djernaes M, Cotton AM, Vane-Wright R, Sperling FAH (2010). Phylogenetics and divergence times of Papilioninae (Lepidoptera) with special reference to the enigmatic genera *Teinopalpus* and *Meandrusa*. Cladistics.

[63] Nazari V, Zakharov EV, Sperling FA (2007). Phylogeny, historical biogeography, and taxonomic ranking of Parnassiinae (Lepidoptera, Papilionidae) based on morphology and seven genes. Mol Phylogenet Evol.

[64] Graybeal A (1998). Is it better to add taxa or characters to a difficult phylogenetic problem?. Syst Biol.

[65] Bazinet AL, Cummings MP, Mitter KT, Mitter CW (2013). Can RNA-Seq resolve the rapid radiation of advanced moths and butterflies (Hexapoda: Lepidoptera: Apoditrysia)? An exploratory study. PLoS One.

[66] Regier JC, Mitter C, Zwick A, Bazinet AL, Cummings MP, Kawahara AY, Sohn J-C, Zwickl DJ, Cho S, Davis DR (2013). A large-scale, higher-level, molecular phylogenetic study of the insect order Lepidoptera (moths and butterflies). PLoS One.

[67] Cho S, Zwick A, Regier JC, Mitter C, Cummings MP, Yao J, Du Z, Zhao H, Kawahara AY, Weller S (2011). Can deliberately incomplete gene sample augmentation improve a phylogeny estimate for the advanced moths and butterflies (Hexapoda: Lepidoptera)?. Syst Biol.

[68] Zahiri R, Kitching IJ, Lafontaine JD, Mutanen M, Kaila L, Holloway JD, Wahlberg N (2011). A new molecular phylogeny offers hope for a stable family level classification of the Noctuoidea (Lepidoptera). Zool Scr.

[69] Brown WM, George M, Wilson AC (1979). Rapid evolution of animal mitochondrial DNA. Proc Natl Acad Sci U S A.

[70] Cameron SL, Miller KB, D'Haese CA, Whiting MF, Barker SC (2004). Mitochondrial genome data alone are not enough to unambiguously resolve the relationships of Entognatha, Insecta and Crustacea sensu lato (Arthropoda). Cladistics.

[71] Bergsten J (2005). A review of long-branch attraction. Cladistics.

[72] Cao Y-Q, Ma C, Chen J-Y, Yang D-R (2012). The complete mitochondrial genomes of two ghost moths, *Thitarodes renzhiensis* and *Thitarodes yunnanensis*: the ancestral gene arrangement in Lepidoptera. BMC Genomics.

[73] Ohshima I, Tanikawa-Dodo Y, Saigusa T, Nishiyama T, Kitani M, Hasebe M, Mohri H (2010). Phylogeny, biogeography, and host-plant association in the subfamily Apaturinae (Insecta: Lepidoptera: Nymphalidae) inferred from eight nuclear and seven mitochondrial genes. Mol Phylogenet Evol.

[74] Brandley MC, Schmitz A, Reeder TW (2005). Partitioned Bayesian analyses, partition choice, and the phylogenetic relationships of scincid lizards. Syst Biol.

[75] Yang Z (1996). Among-site rate variation and its impact on phylogenetic analyses. Trends Ecol Evol.

[76] Metzker ML (2010). Sequencing technologies - the next generation. Nat Rev Genet.

[77] Krause J, Fu Q, Good JM, Viola B, Shunkov MV, Derevianko AP, Paabo S (2010). The complete mitochondrial DNA genome of an unknown hominin from southern Siberia. Nature.

[78] Knapp M, Hofreiter M (2010). Next generation sequencing of ancient DNA: requirements, strategies and perspectives. Genes.

[79] Craig DW, Pearson JV, Szelinger S, Sekar A, Redman M, Corneveaux JJ, Pawlowski TL, Laub T, Nunn G, Stephan DA (2008). Identification of genetic variants using barcoded multiplexed sequencing. Nat Meth.

[80] Kim MJ, Kang AR, Jeong HC, Kim K-G, Kim I (2011). Reconstructing intraordinal relationships in Lepidoptera using mitochondrial genome data with the description of two newly sequenced lycaenids, *Spindasis takanonis* and *Protantigius superans* (Lepidoptera: Lycaenidae). Mol Phylogenet Evol.

[81] Yin J, Wang A-M, Hong G-Y, Cao Y-Z, Wei Z-J (2011). Complete mitochondrial genome of *Chilo suppressalis* (Walker) (Lepidoptera: Crambidae). Mitochondrial DNA.

[82] Ji L, Hao J, Wang Y, Huang D, Zhao J, Zhu C (2012). The complete mitochondrial genome of the dragon swallowtail, *Sericinus montela* Gray (Lepidoptera: Papilionidae) and its phylogenetic implication. Acta Entomol Sin.

[83] Liu G, Jiang G-F, Pang H-C, Hong F (2013). The mitochondrial genome of the Chinese special butterfly *Luehdorfia chinensis* Leech (Lepidoptera: Papilionidae). Mitochondrial DNA.

[84] Qin F, Jiang G-F, Zhou S-Y (2012). Complete mitochondrial genome of the *Teinopalpus aureus guangxiensis* (Lepidoptera: Papilionidae) and related phylogenetic analyses. Mitochondrial DNA.

[85] Kim MI, Baek JY, Kim MJ, Jeong HC, Kim KG, Bae CH, Han YS, Jin BR, Kim I (2009). Complete nucleotide sequence and organization of the mitogenome of the red-spotted apollo butterfly, *Parnassius bremeri* (Lepidoptera: Papilionidae) and comparison with other lepidopteran insects. Mol Cells.

[86] Feng X, Liu D-F, Wang N-X, Zhu C-D, Jiang G-F (2010). The mitochondrial genome of the butterfly *Papilio xuthus* (Lepidoptera: Papilionidae) and related phylogenetic analyses. Mol Biol Rep.

[87] Hao J, Sun Q, Zhao H, Sun X, Gai Y, Yang Q (2012). The complete mitochondrial genome of *Ctenoptilum vasava* (Lepidoptera: Hesperiidae: Pyrginae) and its phylogenetic implication. Comp Funct Genom.

[88] Mao Z, Hao J, Zhu G, Hu J, Si M, Zhu C (2010). Sequencing and analysis of the complete mitochondrial genome of *Pieris rapae* Linnaeus (Lepidoptera: Pieridae). Acta Entomol Sin.

[89] Park JS, Cho Y, Kim MJ, Nam S-H, Kim I (2012). Description of complete mitochondrial genome of the black-veined white, *Aporia crataegi* (Lepidoptera: Papilionoidea), and comparison to papilionoid species. J Asia Pac Entomol.

[90] Shi Q, Xia J, Sun X, Hao J, Yang Q (2012). Complete mitogenome of the Painted Jezebel, *Delias hyparete* Linnaeus (Lepidoptera: Pieridae) and its comparison with other butterfly species. Zool Res.

[91] Kim I, Lee EM, Seol KY, Yun EY, Lee YB, Hwang JS, Jin BR (2006). The mitochondrial genome of the Korean hairstreak, *Coreana raphaelis* (Lepidoptera: Lycaenidae). Insect Mol Biol.

[92] Hao J, Sun M, Shi Q, Sun X, Shao L, Yang Q (2013). Complete mitogenomes of *Euploea mulciber* (Nymphalidae: Danainae) and *Libythea celtis* (Nymphalidae: Libytheinae) and their phylogenetic implications. ISRN Genomics.

[93] Chen M, Tian L-L, Shi Q-H, Cao T-W, Hao J-S (2012). Complete mitogenome of the Lesser Purple Emperor *Apatura ilia* (Lepidoptera: Nymphalidae: Apaturinae) and comparison with other nymphalid butterflies. Zool Res.

[94] Zhang M, Nie X, Cao T, Wang J, Li T, Zhang X, Guo Y, Ma E, Zhong Y (2012). The complete mitochondrial genome of the butterfly *Apatura metis* (Lepidoptera: Nymphalidae). Mol Biol Rep.

[95] Qin X-M, Guan Q-X, Zeng D-L, Qin F, Li H-M (2012). Complete mitochondrial genome of *Kallima inachus* (Lepidoptera: Nymphalidae: Nymphalinae): Comparison of *K. inachus* and *Argynnis hyperbius*. Mitochondrial DNA.

[96] Dasmahapatra KK, Walters JR, Briscoe AD, Davey JW, Whibley A, Nadeau NJ, Zimin AV, Hughes DS, Ferguson LC, Martin SH (2012). Butterfly genome reveals promiscuous exchange of mimicry adaptations among species. Nature.

[97] Hu J, Zhang D, Hao J, Huang D, Cameron S, Zhu C (2010). The complete mitochondrial genome of the yellow coaster, *Acraea issoria* (Lepidoptera: Nymphalidae: Heliconiinae: Acraeini): sequence, gene organization and a unique tRNA translocation event. Mol Biol Rep.

[98] Wang XC, Sun XY, Sun QQ, Zhang DX, Hu J, Yang Q, Hao JS (2011). Complete mitochondrial genome of the laced fritillary *Argyreus hyperbius* (Lepidoptera: Nymphalidae). Dongwuxue Yanjiu.

[99] Kim MJ, Jeong HC, Kim SR, Kim I (2011). Complete mitochondrial genome of the nerippe fritillary butterfly, *Argynnis nerippe* (Lepidoptera: Nymphalidae). Mitochondrial DNA.

[100] Xia J, Hu J, Zhu G-P, Zhu C-D, Hao J-S (2011). Sequencing and analysis of the complete mitochondrial genome of *Calinaga davidis* Oberthür (Lepidoptera: Nymphalidae). Acta Entomol Sin.

[101] Kim MJ, Wan X, Kim K, Hwang JS, Kim I (2010). Complete nucleotide sequence and organization of the mitogenome of endangered *Eumenis autonoe* (Lepidoptera: Nymphalidae). Afr J Biotechnol.

[102] Tian L-L, Sun X-Y, Chen M, Gai Y-H, Hao J-S, Yang Q (2012). Complete mitochondrial genome of the five-dot sergeant *Parathyma sulpitia* (Nymphalidae: Limenitidinae) and its phylogenetic implications. Zool Res.

[103] Hong G, Jiang S, Yu M, Yang Y, Li F, Xue F, Wei Z (2009). The complete nucleotide sequence of the mitochondrial genome of the cabbage butterfly, *Artogeia melete* (Lepidoptera: Pieridae). Acta Biochim Biophys Sin.

[104] Schloss PD, Westcott SL, Ryabin T, Hall JR, Hartmann M, Hollister EB, Lesniewski RA, Oakley BB, Parks DH, Robinson CJ (2009). Introducing mothur: open-source, platform-independent, community-supported software for describing and comparing microbial communities. Appl Environ Microbiol.

[105] Chevreux B, Wetter T, Suhai S (1999). Genome sequence assembly using trace signals and additional sequence information. Computer Science and Biology: Proceedings of the German Conference on Bioinformatics (GCB).

[106] Bernt M, Donath A, Jühling F, Externbrink F, Florentz C, Fritzsch G, Pütz J, Middendorf M, Stadler PF (2013). MITOS: improved *de novo* metazoan mitochondrial genome annotation. Mol Phylogenet Evol.

[107] Tamura K, Peterson D, Peterson N, Stecher G, Nei M, Kumar S (2011). MEGA5: molecular evolutionary genetics analysis using maximum likelihood, evolutionary distance, and maximum parsimony methods. Mol Biol Evol.

[108] Librado P, Rozas J (2009). DnaSP v5: a software for comprehensive analysis of DNA polymorphism data. Bioinformatics.

[109] Darriba D, Taboada GL, Doallo R, Posada D (2012). jModelTest 2: more models, new heuristics and parallel computing. Nat Meth.

[110] Goloboff PA, Farris JS, Nixon KC (2008). TNT, a free program for phylogenetic analysis. Cladistics.

[111] Ronquist F, Teslenko M, van der Mark P, Ayres DL, Darling A, Höhna S, Larget B, Liu L, Suchard MA, Huelsenbeck JP (2012). MrBayes 3.2: efficient Bayesian phylogenetic inference and model choice across a large model space. Syst Biol.

[112] Stamatakis A (2006). RAxML-VI-HPC: maximum likelihood-based phylogenetic analyses with thousands of taxa and mixed models. Bioinformatics.

[113] Ott M, Zola J, Stamatakis A, Aluru S (2007). Large-scale maximum likelihood-based phylogenetic analysis on the IBM BlueGene/L. Proceedings of the 2007 ACM/IEEE conference on Supercomputing: 2007.

[114] McGuire JA, Witt CC, Altshuler DL, Remsen JV (2007). Phylogenetic systematics and biogeography of hummingbirds: Bayesian and maximum likelihood analyses of partitioned data and selection of an appropriate partitioning strategy. Syst Biol.

[115] Ren F, Tanaka H, Yang Z (2005). An empirical examination of the utility of codon-substitution models in phylogeny reconstruction. Syst Biol.

[116] Goloboff PA (1999). Analyzing large data sets in reasonable times: solutions for composite optima. Cladistics.

[117] Kass RE, Raftery AE (1995). Bayes factors. J Am Stat Assoc.

