# Supplementary figures and images for: Mitogenomic sequences effectively recover relationships within brush-footed butterflies (Lepidoptera: Nymphalidae)
Source: BMC Genomics. 2014 Jun 12;15:468. doi: 10.1186/1471-2164-15-468 (PMC4070565; doi:10.1186/1471-2164-15-468)

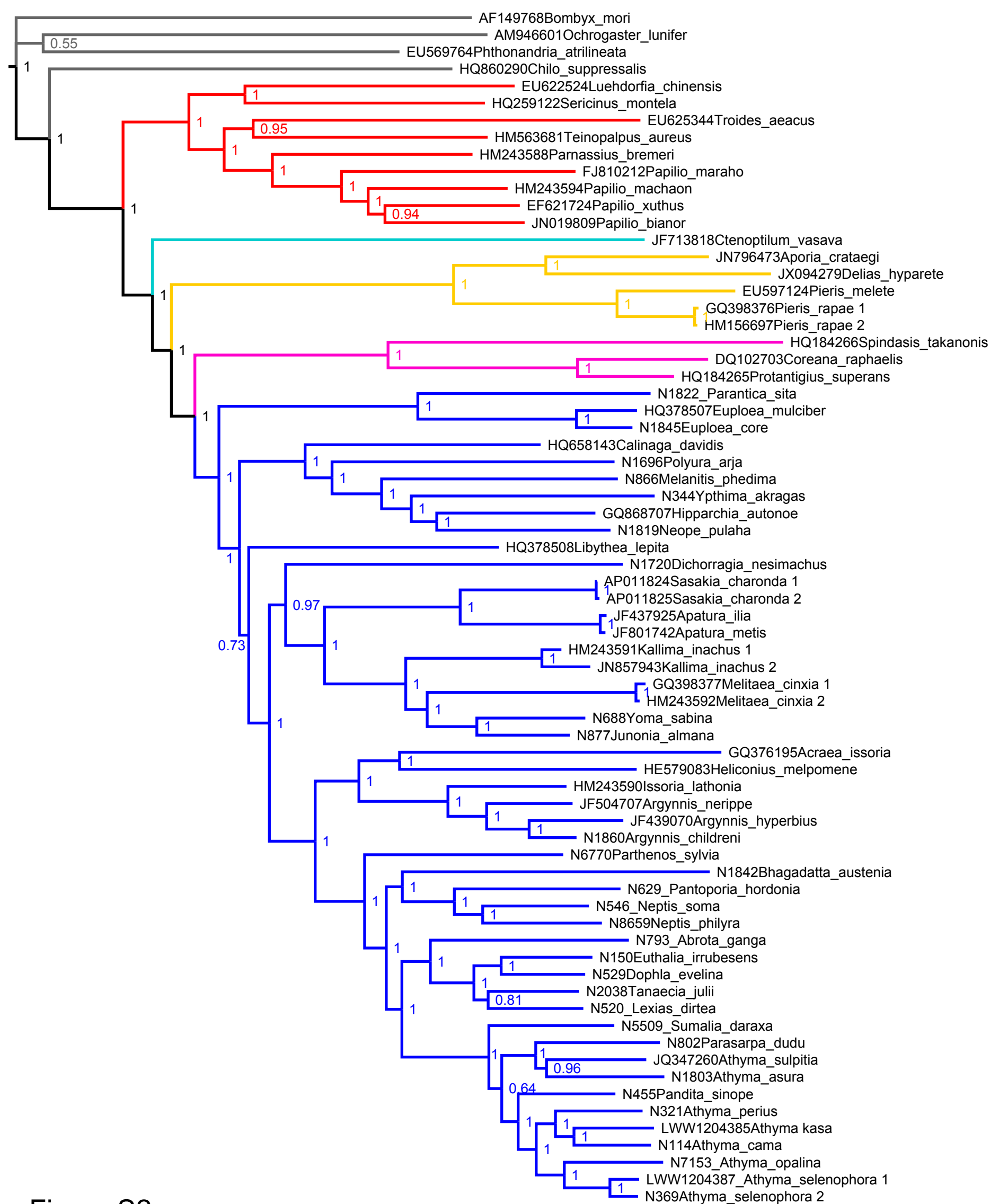

Figure S2

0.1

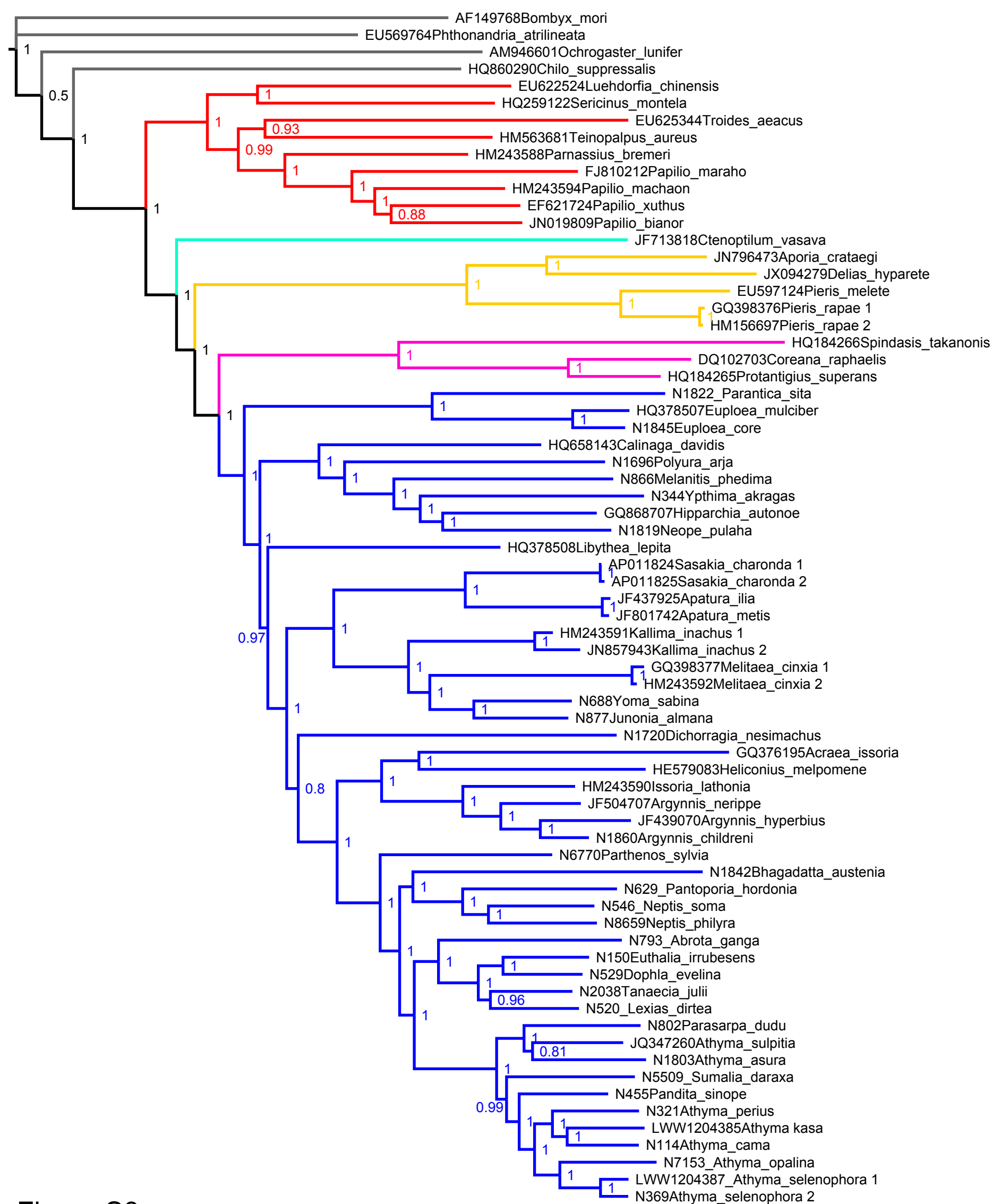

Figure S3

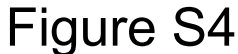

0.1

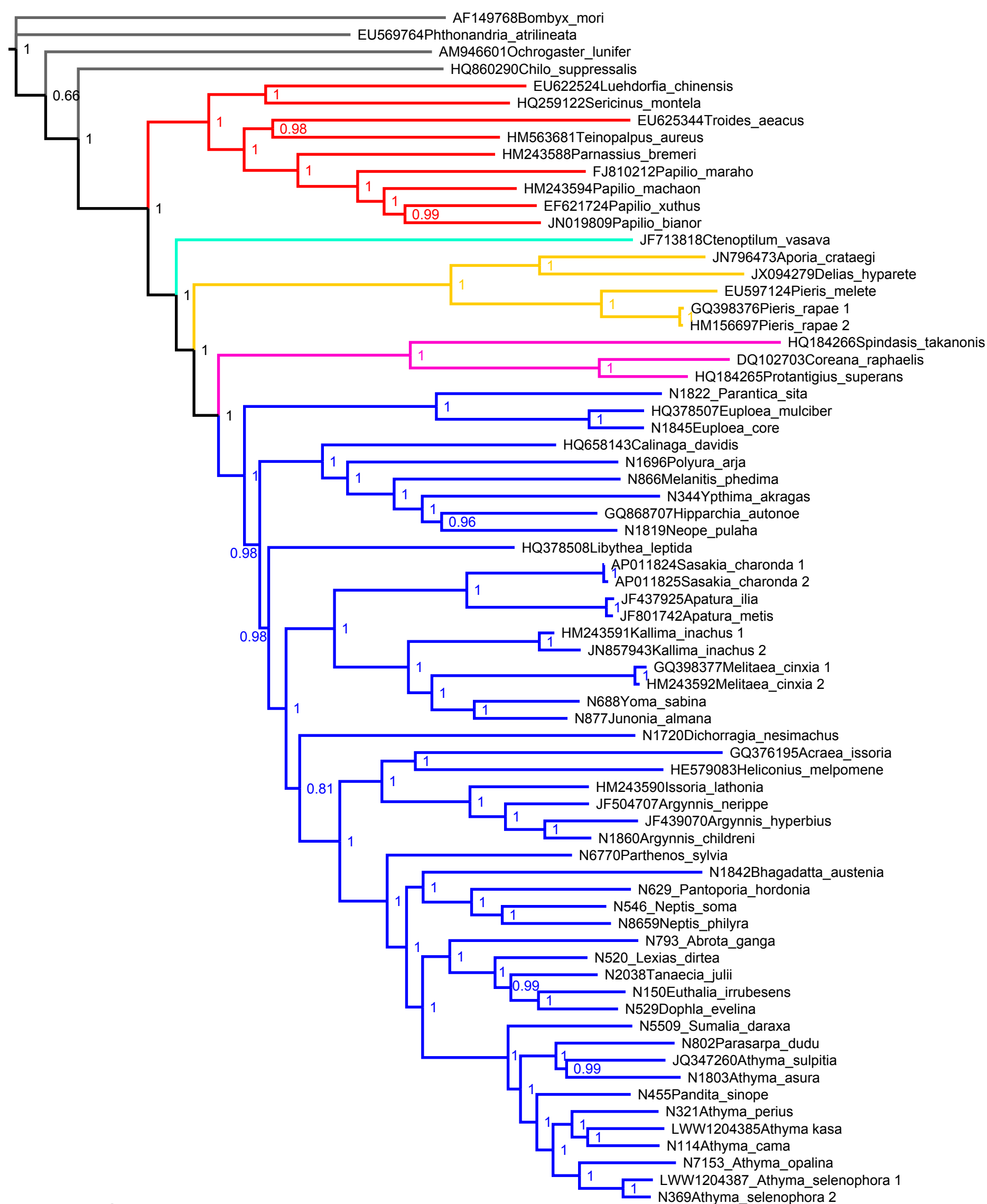

Figure S5

0.1

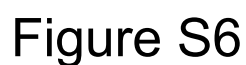

\_\_\_\_\_

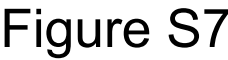

0.1

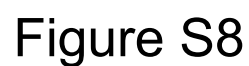

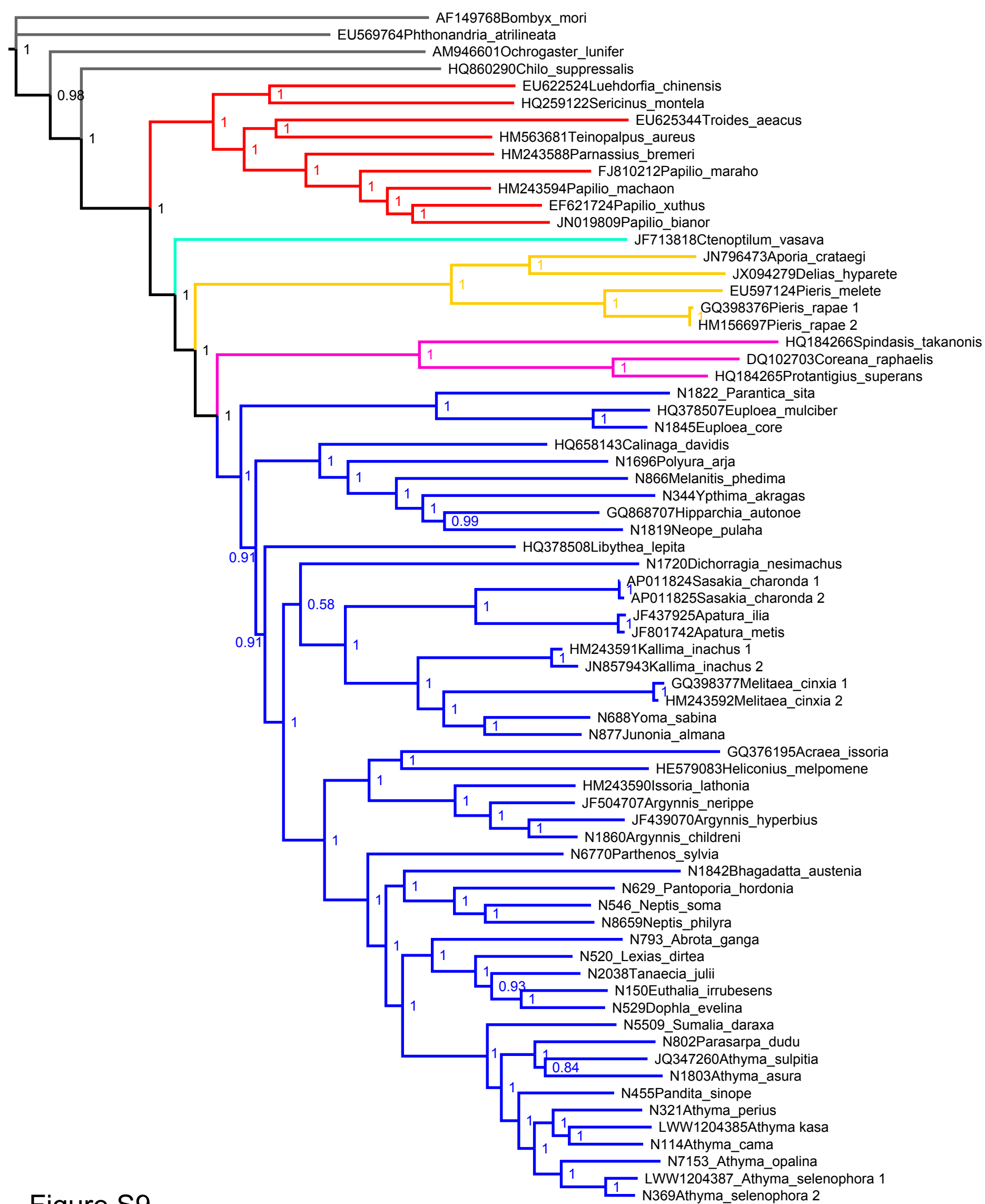

Figure S9





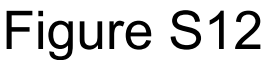

0.1

Supplement: Supplementary file 6 — Additional file 6: Figures S1-S12: Bayesian trees based on PS1-12 and the GTR + G model. Values at nodes correspond to posterior probabilities. (PDF 2 MB) [file 12864_2013_6134_MOESM6_ESM.pdf]

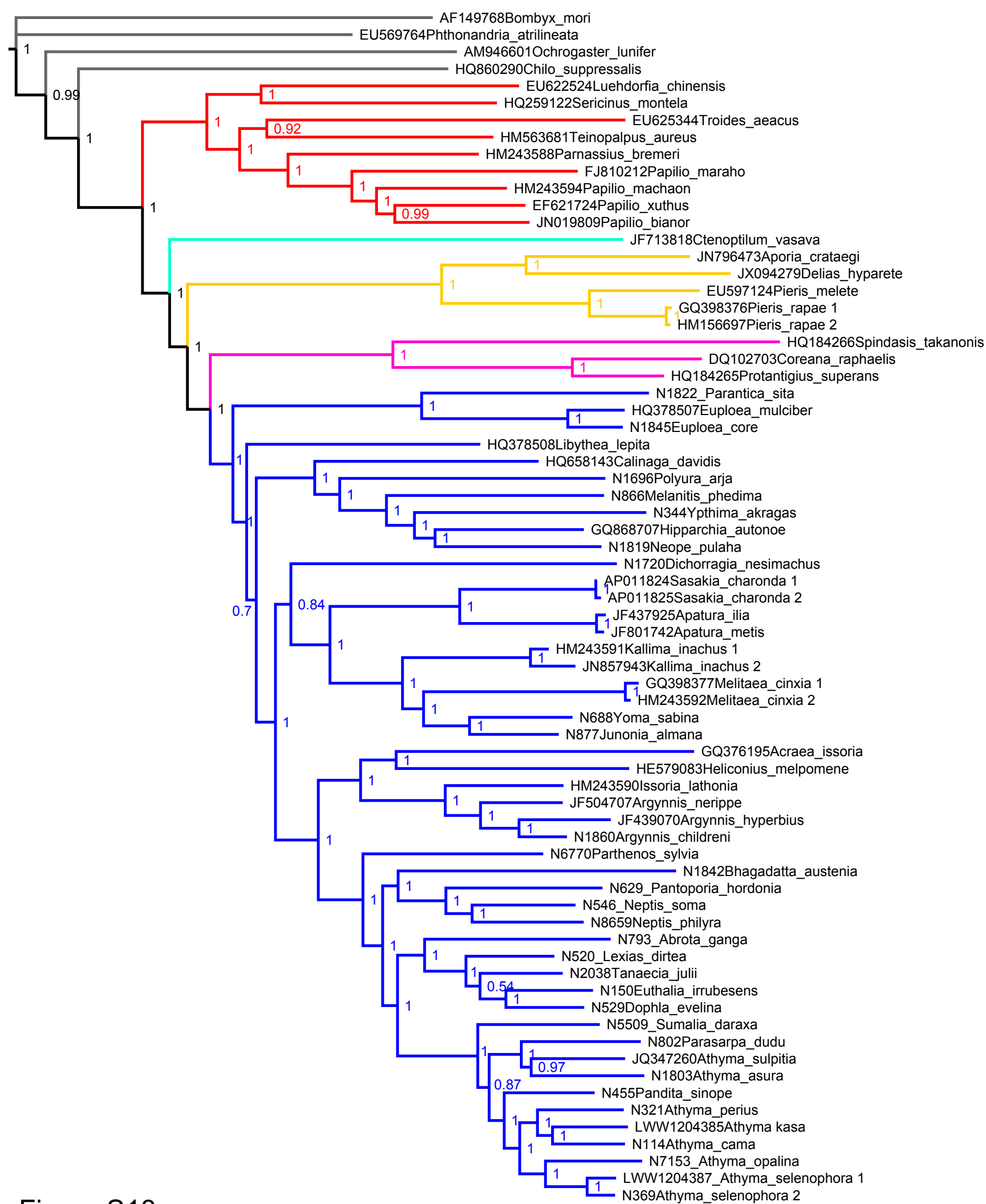

Figure S13

0.08

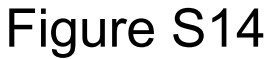

0.1

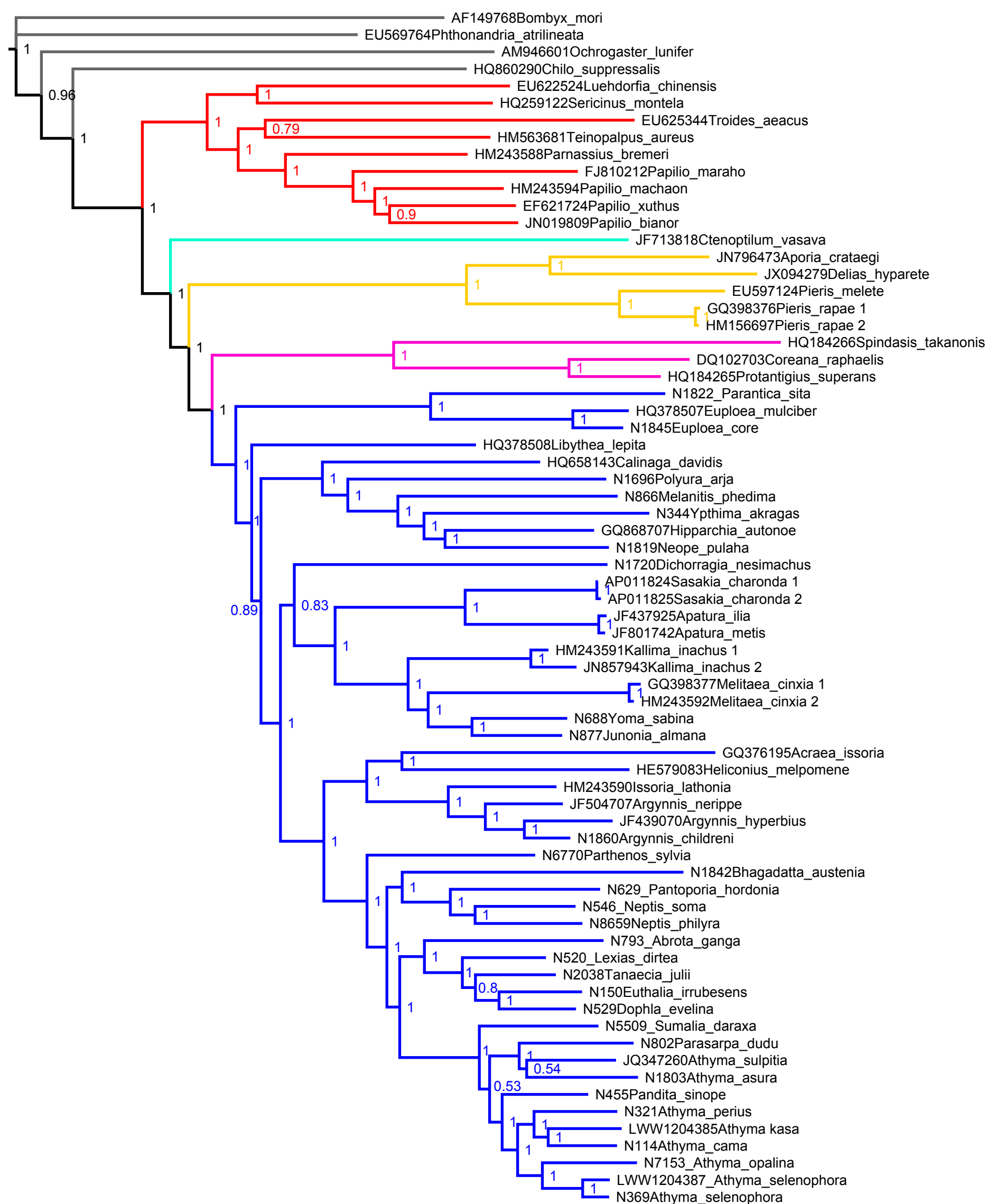

Figure S15

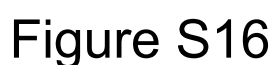

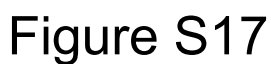

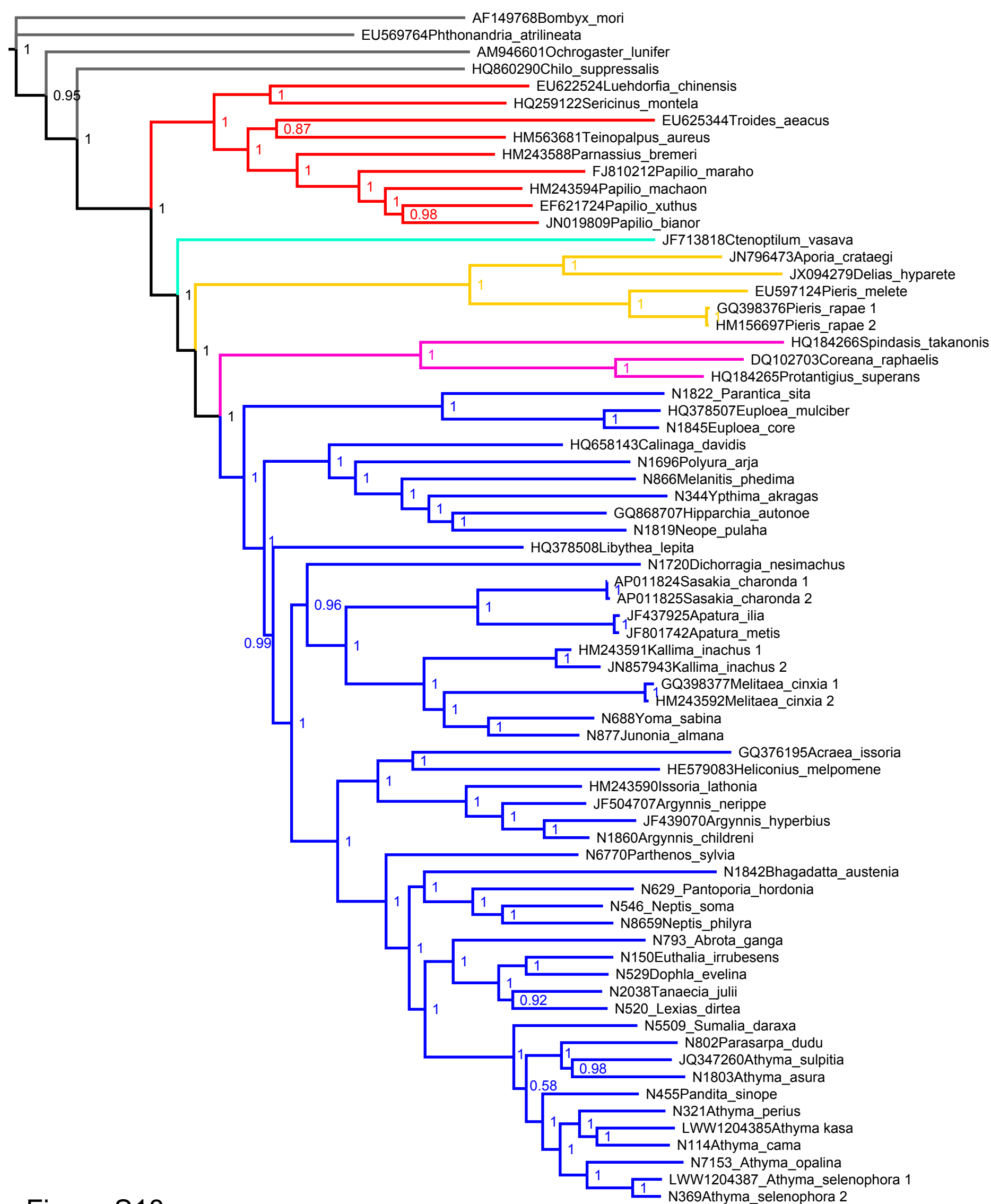

Figure S18

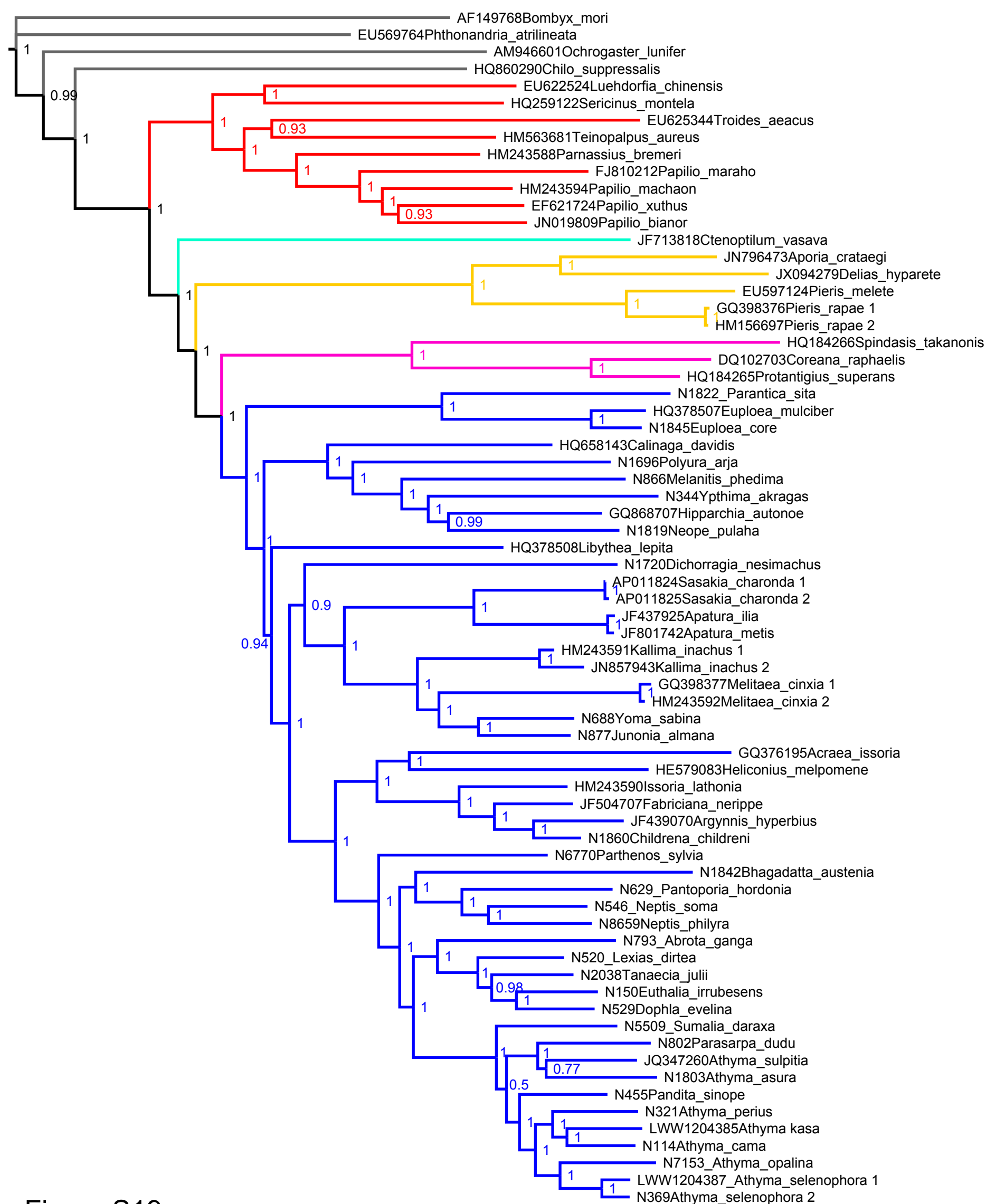

Figure S19

0.1

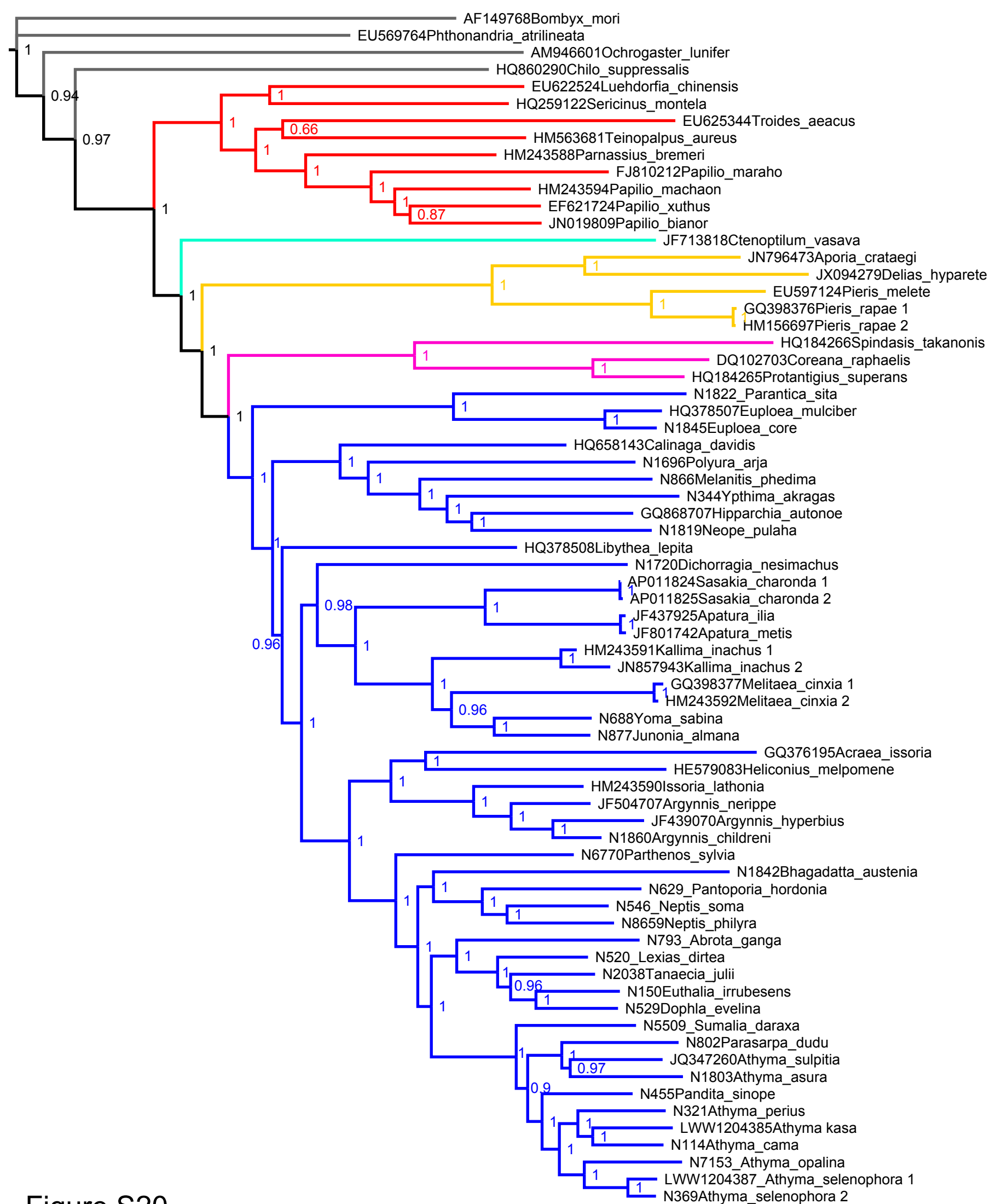

Figure S20

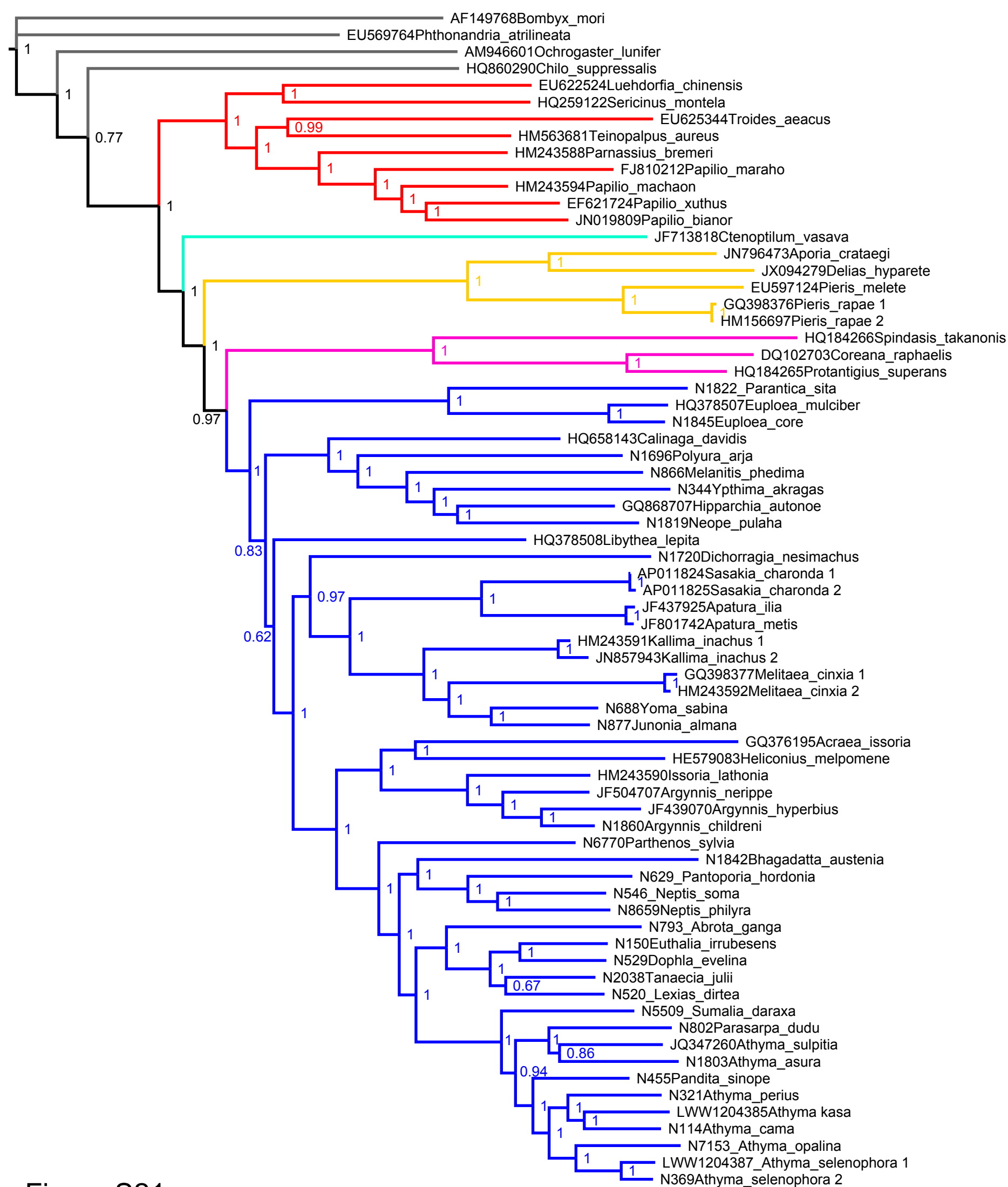

Figure S21

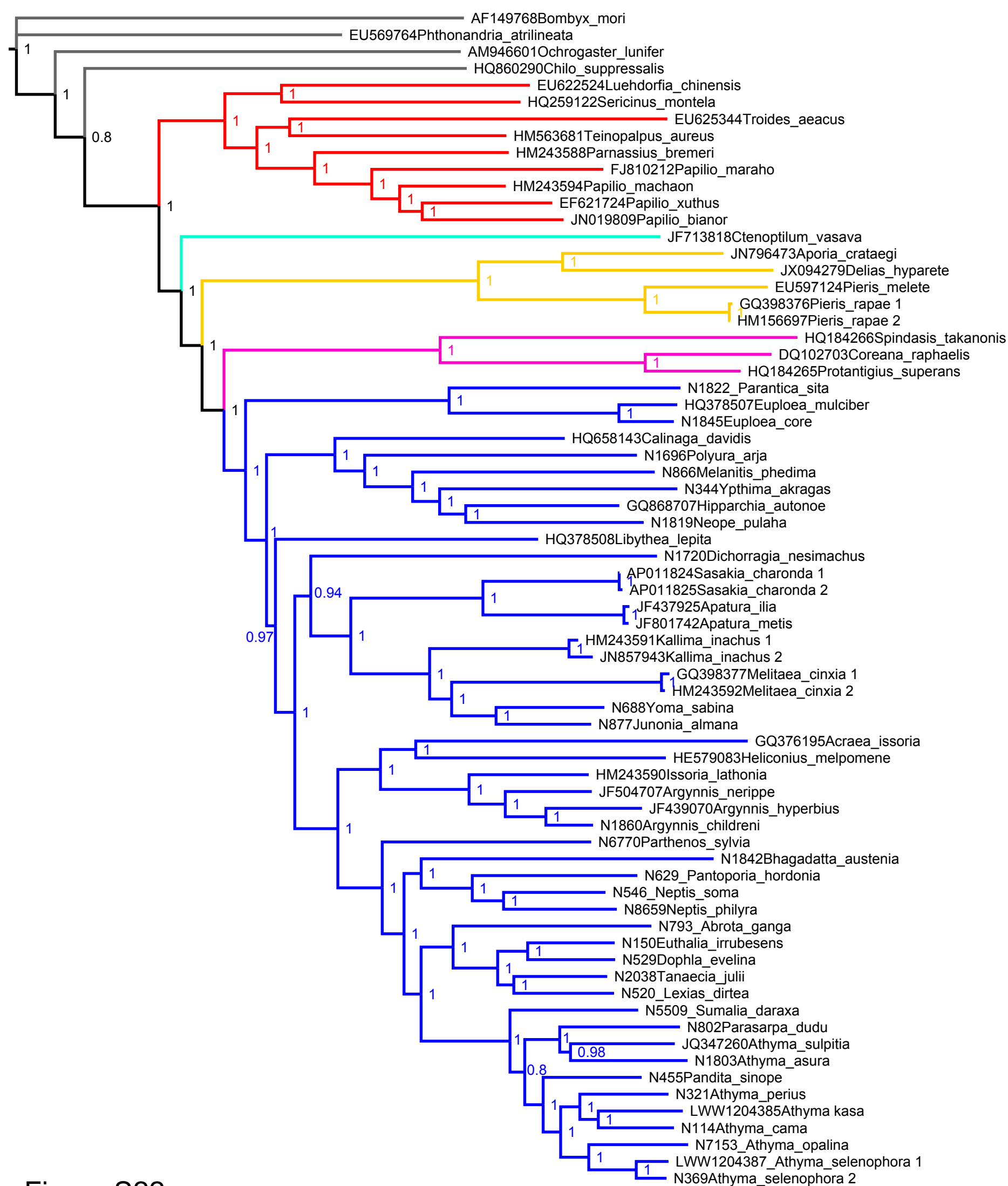

Figure S22

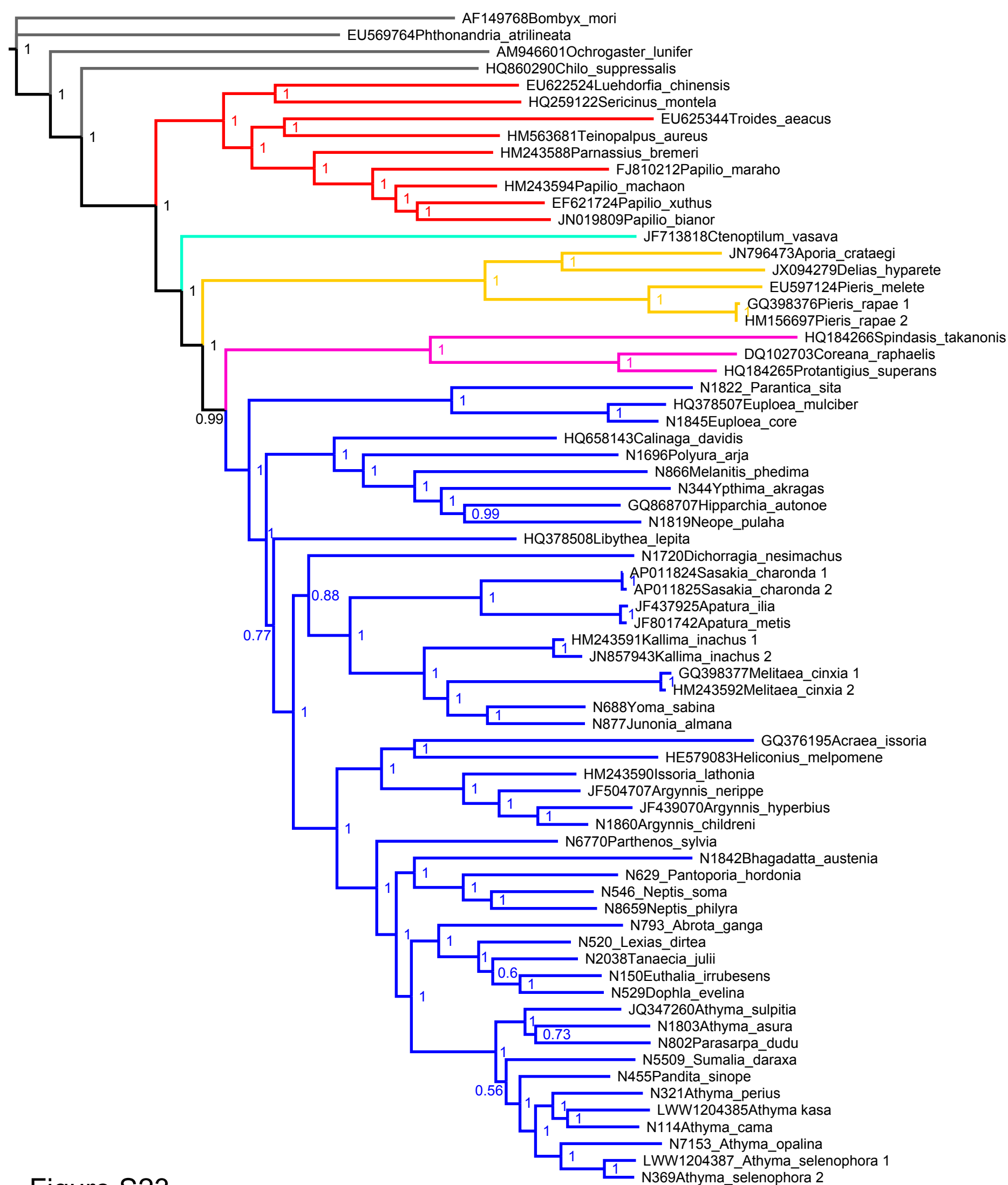

Figure S23

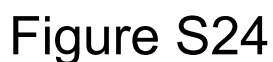

Supplement: Supplementary file 7 — Additional file 7: Figures S13-S24: Bayesian trees based on PS1-12 and the best-fit model. Values at nodes correspond to posterior probabilities. (PDF 2 MB) [file 12864_2013_6134_MOESM7_ESM.pdf]

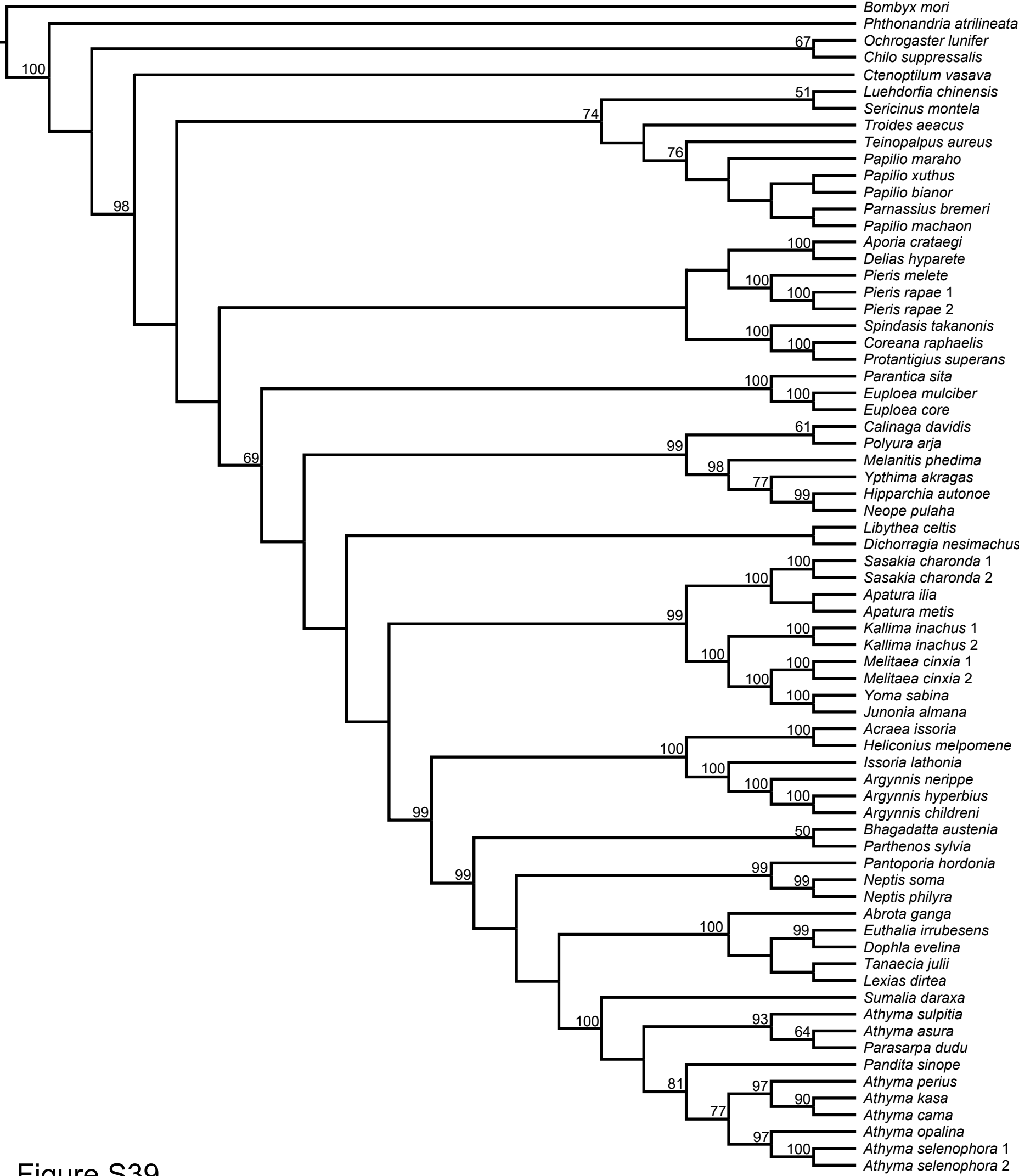

Figure S39

Supplement: Supplementary file 10 — Additional file 10: Figure S38-S39: Maximum-parsimony topology based on 15 and 37-gene datasets, respectively. The seven and 19 most parsimonious trees were summarised by 50% majority-rule in Figure S38, and Figure S39, respectively. Bootstrap values over 50% are shown above the branches. (PDF 334 KB) [file 12864_2013_6134_MOESM10_ESM.pdf]

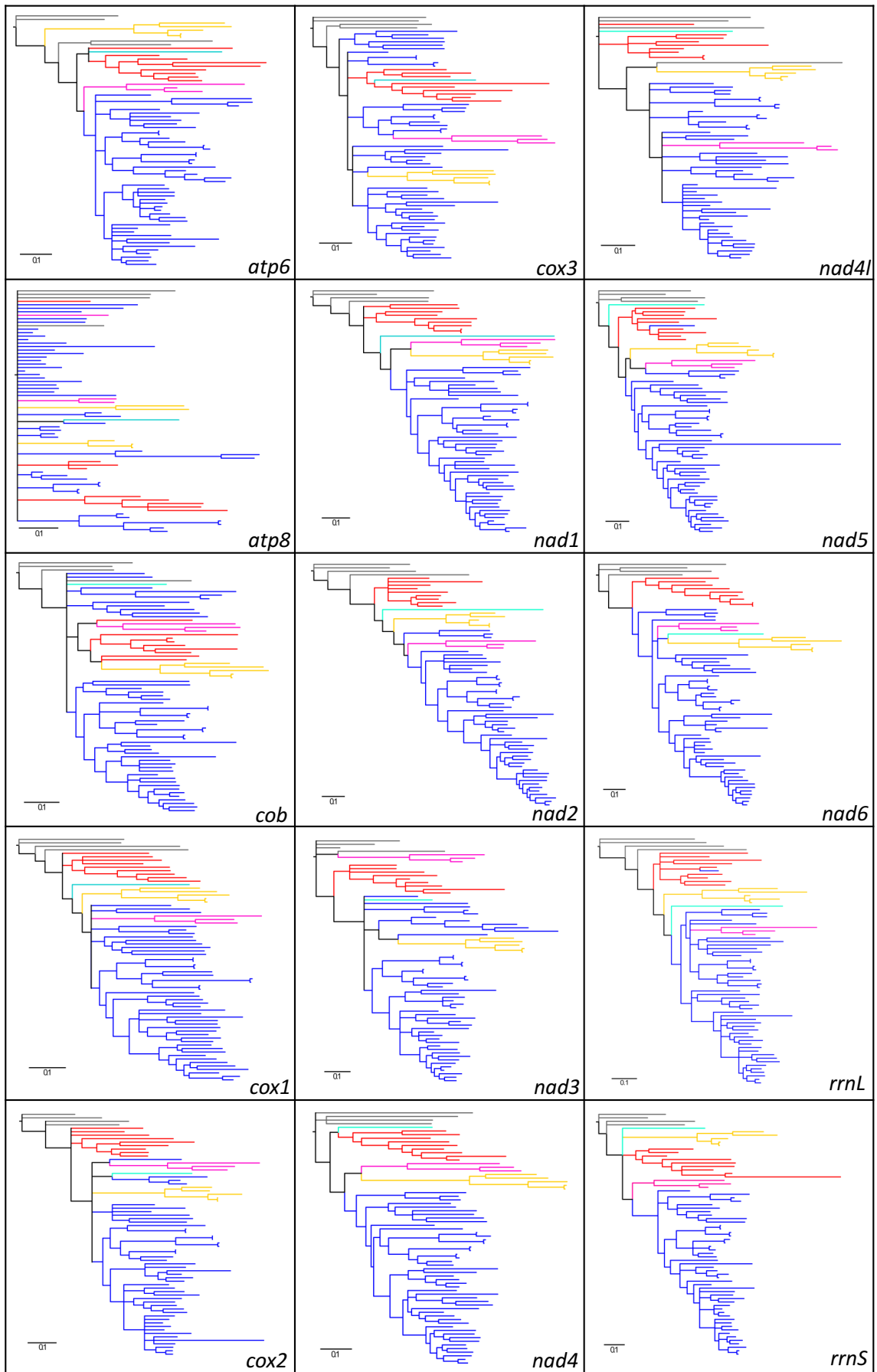

Supplement: Supplementary file 11 — Additional file 11: Figure S40: The Bayesian phylogeny for each gene. Grey: outgroups; red: Papilionidae; cyan: Hesperiidae; yellow: Pieridae; magenta: Lycaenidae; blue: Nymphalidae. (PDF 167 KB) [file 12864_2013_6134_MOESM11_ESM.pdf]
