# Supplementary material for: Mitogenomic sequences effectively recover relationships within brush-footed butterflies (Lepidoptera: Nymphalidae)
Source: BMC Genomics. 2014 Jun 12;15:468. doi: 10.1186/1471-2164-15-468 (PMC4070565; doi:10.1186/1471-2164-15-468)
Supplement: Supplementary file 8 — Additional file 8: Figures S25-S36: The ML phylogeny based on PS1-12 and the GTR + G model. Numbers above branches denote bootstrap support. (PDF 2 MB) [file 12864_2013_6134_MOESM8_ESM.pdf]

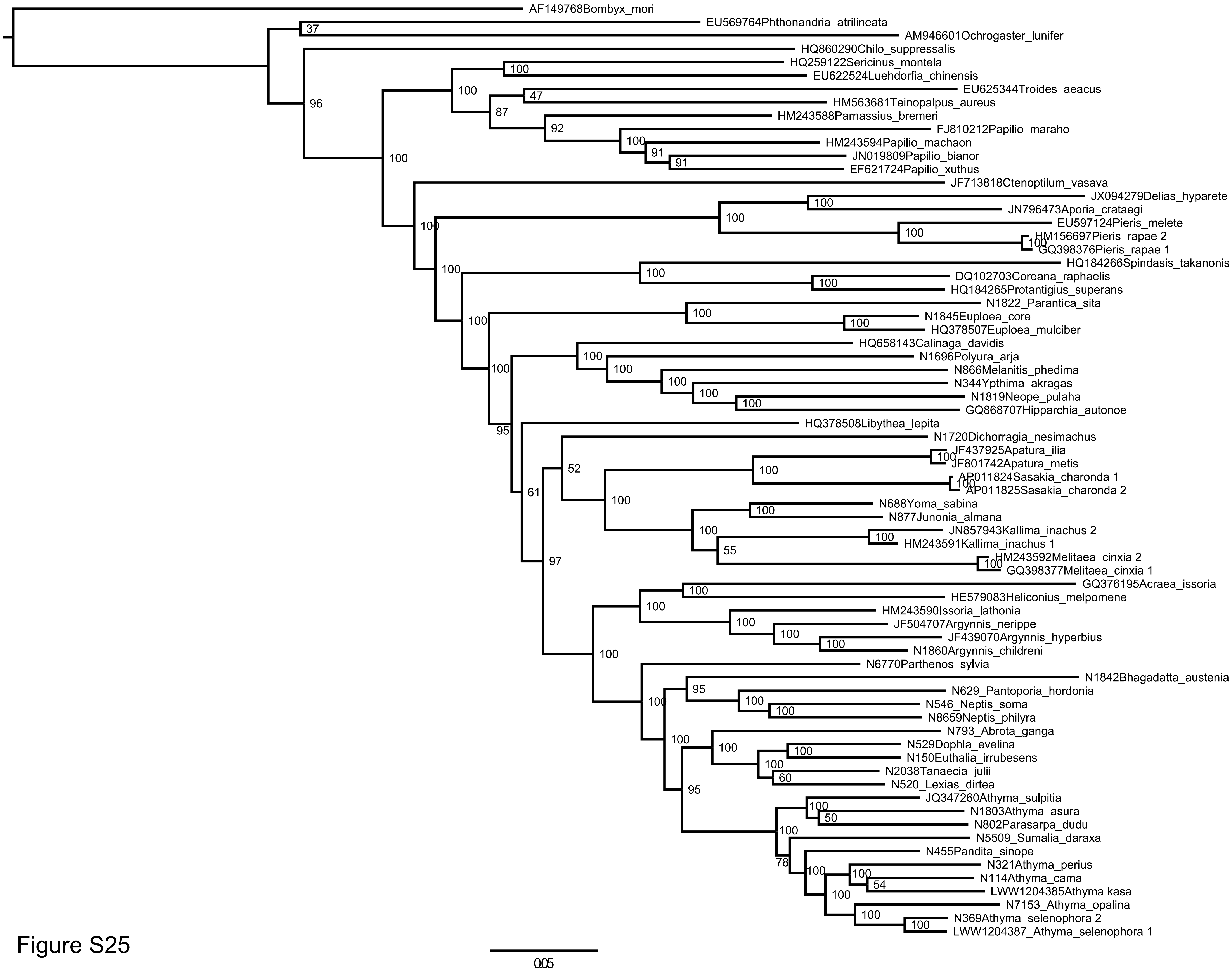
AF149768Bombyx_mori
EU569764Phthonandria_atrilineata
AM946601Ochrogaster_lunifer
HQ860290Chilo_suppressalis
HQ259122Sericinus_montela
EU622524Luehdorfia_chinensis
EU625344Troides_aeacus
HM563681Teinopalpus_aureus
HM243588Parnassius_bremeri
FJ810212Papilio_maraho
HM243594Papilio_machaon
JN019809Papilio_bianor
EF621724Papilio_xuthus
JF713818Ctenoptilum_vasava
JX094279Delias_hyparete
JN796473Aporia_crataegi
EU597124Pieris_melete
HM156697Pieris_rapae 2
GQ398376Pieris_rapae 1
HQ184266Spindasis_takanonis
DQ102703Coreana_raphaelis
HQ184265Protantigius_superans
N1822_Parantica_sita
N1845Euploea_core
HQ378507Euploea_mulciber
HQ658143Calinaga_davidis
N1696Polyura_arja
N866Melanitis_phedima
N344Ypthima_akragas
N1819Neope_pulaha
GQ868707Hipparchia_autonoe
HQ378508Libythea_lepita
N1720Dichorragia_nesimachus
JF437925Apatura_ilia
JF801742Apatura_metis
AP011824Sasakia_charonda 1
AP011825Sasakia_charonda 2
N688Yoma_sabina
N877Junonia_almana
JN857943Kallima_inachus 2
HM243591Kallima_inachus 1
HM243592Melitaea_cinxia 2
GQ398377Melitaea_cinxia 1
GQ376195Acraea_issoria
HE579083Heliconius_melpomene
HM243590Issoria_lathonia
JF504707Argynnis_nerippe
JF439070Argynnis_hyperbius
N1860Argynnis_childreni
N6770Parthenos_sylvia
N1842Bhagadatta_austenia
N629_Pantoporia_hordonia
N546_Neptis_soma
N8659Neptis_philyra
N793_Abrota_ganga
N529Dophla_evelina
N150Euthalia_irrubesens
N2038Tanaecia_julii
N520_Lexias_dirtea
JQ347260Athyma_sulpitia
N1803Athyma_asura
N802Parasarpa_dudu
N5509_Sumalia_daraxa
N455Pandita_sinope
N321Athyma_perius
N114Athyma_cama
LWW1204385Athyma kasa
N7153_Athyma_opalina
N369Athyma_selenophora 2
LWW1204387_Athyma_selenophora 1
0.05

Figure S25

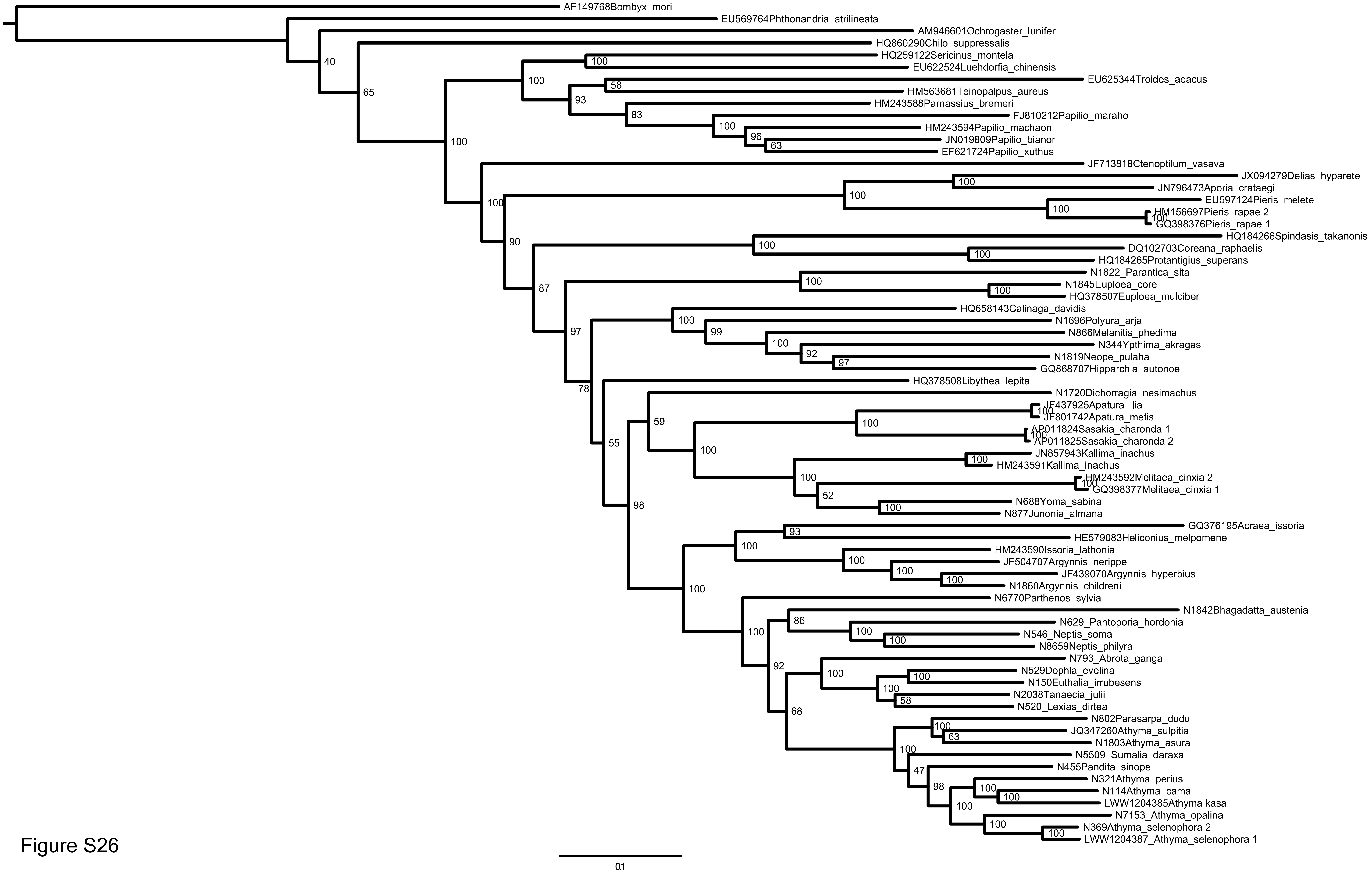
AF149768Bombyx_mori
EU569764Phthonandria_atrilineata
AM946601Ochrogaster_lunifer
HQ860290Chilo_suppressalis
HQ259122Sericinus_montela
EU622524Luehdorfia_chinensis
EU625344Troides_aeacus
HM563681Teinopalpus_aureus
HM243588Parnassius_bremeri
FJ810212Papilio_maraho
HM243594Papilio_machaon
JN019809Papilio_bianor
EF621724Papilio_xuthus
JF713818Ctenoptilum_vasava
JX094279Delias_hyparete
JN796473Aporia_crataegi
EU597124Pieris_melete
HM156697Pieris_rapae 2
GQ398376Pieris_rapae 1
HQ184266Spindasis_takanonis
DQ102703Coreana_raphaelis
HQ184265Protantigius_superans
N1822_Parantica_sita
N1845Euploea_core
HQ378507Euploea_mulciber
HQ658143Calinaga_davidis
N1696Polyura_arja
N866Melanitis_phedima
N344Ypthima_akragas
N1819Neope_pulaha
GQ868707Hipparchia_autonoe
HQ378508Libythea_lepita
N1720Dichorragia_nesimachus
JF437925Apatura_ilia
JF801742Apatura_metis
AP011824Sasakia_charonda 1
AP011825Sasakia_charonda 2
JN857943Kallima_inachus
HM243591Kallima_inachus
HM243592Melitaea_cinxia 2
GQ398377Melitaea_cinxia 1
N688Yoma_sabina
N877Junonia_almana
GQ376195Acraea_issoria
HE579083Heliconius_melpomene
HM243590Issoria_lathonia
JF504707Argynnis_nerippe
JF439070Argynnis_hyperbius
N1860Argynnis_childreni
N6770Parthenos_sylvia
N1842Bhagadatta_austenia
N629_Pantoporia_hordonia
N546_Neptis_soma
N8659Neptis_philyra
N793_Abrota_ganga
N529Dophla_evelina
N150Euthalia_irrubesens
N2038Tanaecia_julii
N520_Lexias_dirtea
N802Parasarpa_dudu
JQ347260Athyma_sulpitia
N1803Athyma_asura
N5509_Sumalia_daraxa
N455Pandita_sinope
N321Athyma_perius
N114Athyma_cama
LWW1204385Athyma kasa
N7153_Athyma_opalina
N369Athyma_selenophora 2
LWW1204387_Athyma_selenophora 1
01

Figure S26

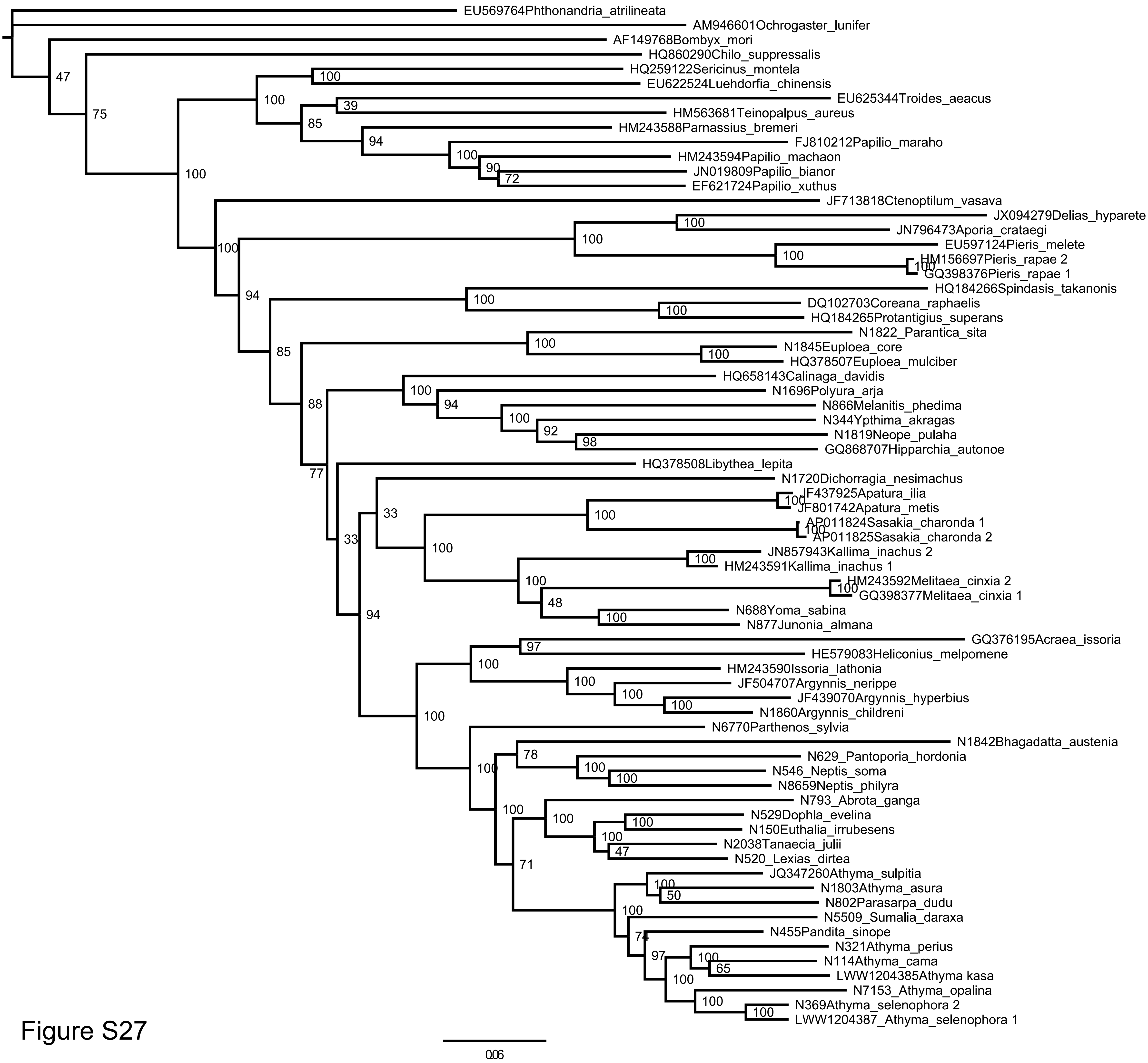
EU569764Phthonandria_atrilineata
AM946601Ochrogaster_lunifer
AF149768Bombyx_mori
HQ860290Chilo_suppressalis
HQ259122Sericinus_montela
EU622524Luehdorfia_chinensis
EU625344Troides_aeacus
HM563681Teinopalpus_aureus
HM243588Parnassius_bremeri
FJ810212Papilio_maraho
HM243594Papilio_machaon
JN019809Papilio_bianor
EF621724Papilio_xuthus
JF713818Ctenoptilum_vasava
JX094279Delias_hyparete
JN796473Aporia_crataegi
EU597124Pieris_melete
HM156697Pieris_rapae 2
GQ398376Pieris_rapae 1
HQ184266Spindasis_takanonis
DQ102703Coreana_raphaelis
HQ184265Protantigius_superans
N1822_Parantica_sita
N1845Euploea_core
HQ378507Euploea_mulciber
HQ658143Calinaga_davidis
N1696Polyura_arja
N866Melanitis_phedima
N344Ypthima_akragas
N1819Neope_pulaha
GQ868707Hipparchia_autonoe
HQ378508Libythea_lepita
N1720Dichorragia_nesimachus
JF437925Apatura_ilia
JF801742Apatura_metis
AP011824Sasakia_charonda 1
AP011825Sasakia_charonda 2
JN857943Kallima_inachus 2
HM243591Kallima_inachus 1
HM243592Melitaea_cinxia 2
GQ398377Melitaea_cinxia 1
N688Yoma_sabina
N877Junonia_almana
GQ376195Acraea_issoria
HE579083Heliconius_melpomene
HM243590Issoria_lathonia
JF504707Argynnis_nerippe
JF439070Argynnis_hyperbius
N1860Argynnis_childreni
N6770Parthenos_sylvia
N1842Bhagadatta_austenia
N629_Pantoporia_hordonia
N546_Neptis_soma
N8659Neptis_philyra
N793_Abrota_ganga
N529Dophla_evelina
N150Euthalia_irrubesens
N2038Tanaecia_julii
N520_Lexias_dirtea
JQ347260Athyma_sulpitia
N1803Athyma_asura
N802Parasarpa_dudu
N5509_Sumalia_daraxa
N455Pandita_sinope
N321Athyma_perius
N114Athyma_cama
LWW1204385Athyma kasa
N7153_Athyma_opalina
N369Athyma_selenophora 2
LWW1204387_Athyma_selenophora 1
0.06

Figure S27

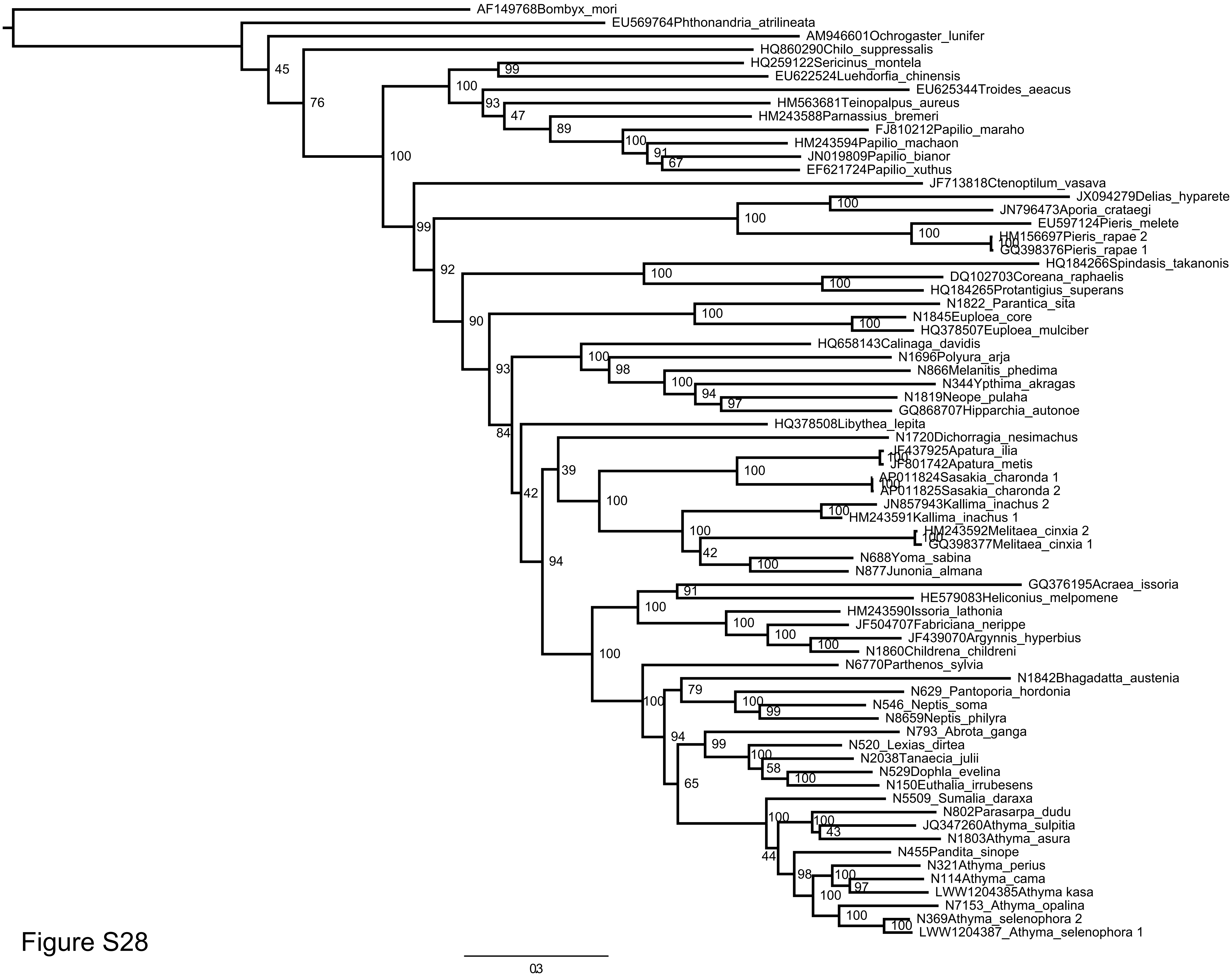
AF149768Bombyx_mori
EU569764Phthonandria_atrilineata
AM946601Ochrogaster_lunifer
HQ860290Chilo_suppressalis
HQ259122Sericinus_montela
EU622524Luehdorfia_chinensis
EU625344Troides_aeacus
HM563681Teinopalpus_aureus
HM243588Parnassius_bremeri
FJ810212Papilio_maraho
HM243594Papilio_machaon
JN019809Papilio_bianor
EF621724Papilio_xuthus
JF713818Ctenoptilum_vasava
JX094279Delias_hyparete
JN796473Aporia_crataegi
EU597124Pieris_melete
HM156697Pieris_rapae 2
GQ398376Pieris_rapae 1
HQ184266Spindasis_takanonis
DQ102703Coreana_raphaelis
HQ184265Protantigius_superans
N1822_Parantica_sita
N1845Euploea_core
HQ378507Euploea_mulciber
HQ658143Calinaga_davidis
N1696Polyura_arja
N866Melanitis_phedima
N344Ypthima_akragas
N1819Neope_pulaha
GQ868707Hipparchia_autonoe
HQ378508Libythea_lepita
N1720Dichorragia_nesimachus
JF437925Apatura_ilia
JF801742Apatura_metis
AP011824Sasakia_charonda 1
AP011825Sasakia_charonda 2
JN857943Kallima_inachus 2
HM243591Kallima_inachus 1
HM243592Melitaea_cinxia 2
GQ398377Melitaea_cinxia 1
N688Yoma_sabina
N877Junonia_almana
GQ376195Acraea_issoria
HE579083Heliconius_melpomene
HM243590Issoria_lathonia
JF504707Fabriciana_nerippe
JF439070Argynnis_hyperbius
N1860Childrena_childreni
N6770Parthenos_sylvia
N1842Bhagadatta_austenia
N629_Pantoporia_hordonia
N546_Neptis_soma
N8659Neptis_philyra
N793_Abrota_ganga
N520_Lexias_dirtea
N2038Tanaecia_julii
N529Dophla_evelina
N150Euthalia_irrubesens
N5509_Sumalia_daraxa
N802Parasarpa_dudu
JQ347260Athyma_sulpitia
N1803Athyma_asura
N455Pandita_sinope
N321Athyma_perius
N114Athyma_cama
LWW1204385Athyma kasa
N7153_Athyma_opalina
N369Athyma_selenophora 2
LWW1204387_Athyma_selenophora 1
0.3

Figure S28

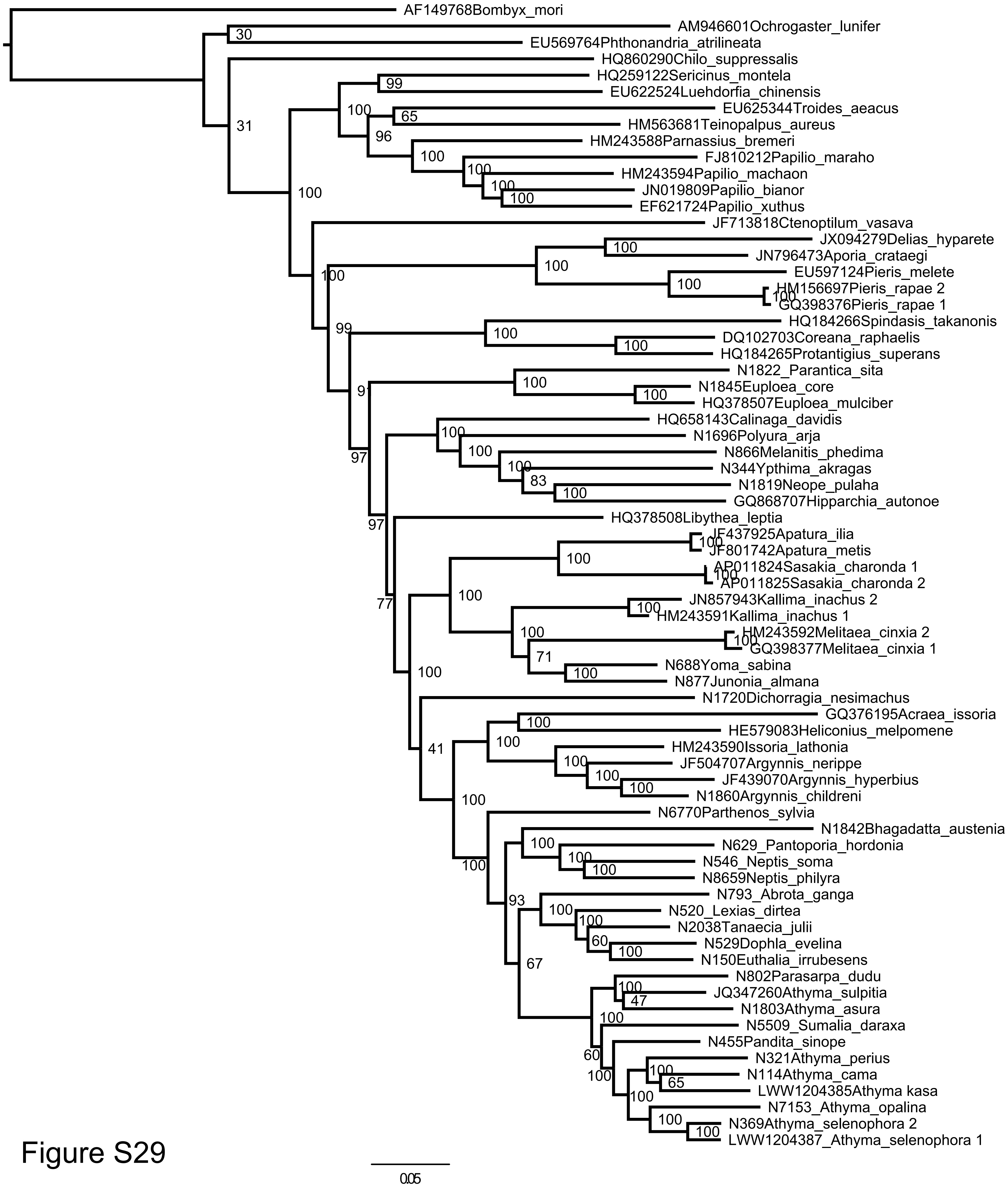
AF149768Bombyx_mori
AM946601Ochrogaster_lunifer
EU569764Phthonandria_atrilineata
HQ860290Chilo_suppressalis
HQ259122Sericinus_montela
EU622524Luehdorfia_chinensis
EU625344Troides_aeacus
HM563681Teinopalpus_aureus
HM243588Parnassius_bremeri
FJ810212Papilio_maraho
HM243594Papilio_machaon
JN019809Papilio_bianor
EF621724Papilio_xuthus
JF713818Ctenoptilum_vasava
JX094279Delias_hyparete
JN796473Aporia_crataegi
EU597124Pieris_melete
HM156697Pieris_rapae 2
GQ398376Pieris_rapae 1
HQ184266Spindasis_takanonis
DQ102703Coreana_raphaelis
HQ184265Protantigius_superans
N1822_Parantica_sita
N1845Euploea_core
HQ378507Euploea_mulciber
HQ658143Calinaga_davidis
N1696Polyura_arja
N866Melanitis_phedima
N344Ypthima_akragas
N1819Neope_pulaha
GQ868707Hipparchia_autonoe
HQ378508Libythea_leptia
JF437925Apatura_ilia
JF801742Apatura_metis
AP011824Sasakia_charonda 1
AP011825Sasakia_charonda 2
JN857943Kallima_inachus 2
HM243591Kallima_inachus 1
HM243592Melitaea_cinxia 2
GQ398377Melitaea_cinxia 1
N688Yoma_sabina
N877Junonia_almana
N1720Dichorragia_nesimachus
GQ376195Acraea_issoria
HE579083Heliconius_melpomene
HM243590Issoria_lathonia
JF504707Argynnis_nerippe
JF439070Argynnis_hyperbius
N1860Argynnis_childreni
N6770Parthenos_sylvia
N1842Bhagadatta_austenia
N629_Pantoporia_hordonia
N546_Neptis_soma
N8659Neptis_philyra
N793_Abrota_ganga
N520_Lexias_dirtea
N2038Tanaecia_julii
N529Dophla_evelina
N150Euthalia_irrubesens
N802Parasarpa_dudu
JQ347260Athyma_sulpitia
N1803Athyma_asura
N5509_Sumalia_daraxa
N455Pandita_sinope
N321Athyma_perius
N114Athyma_cama
LWW1204385Athyma kasa
N7153_Athyma_opalina
N369Athyma_selenophora 2
LWW1204387_Athyma_selenophora 1
0.05

Figure S29

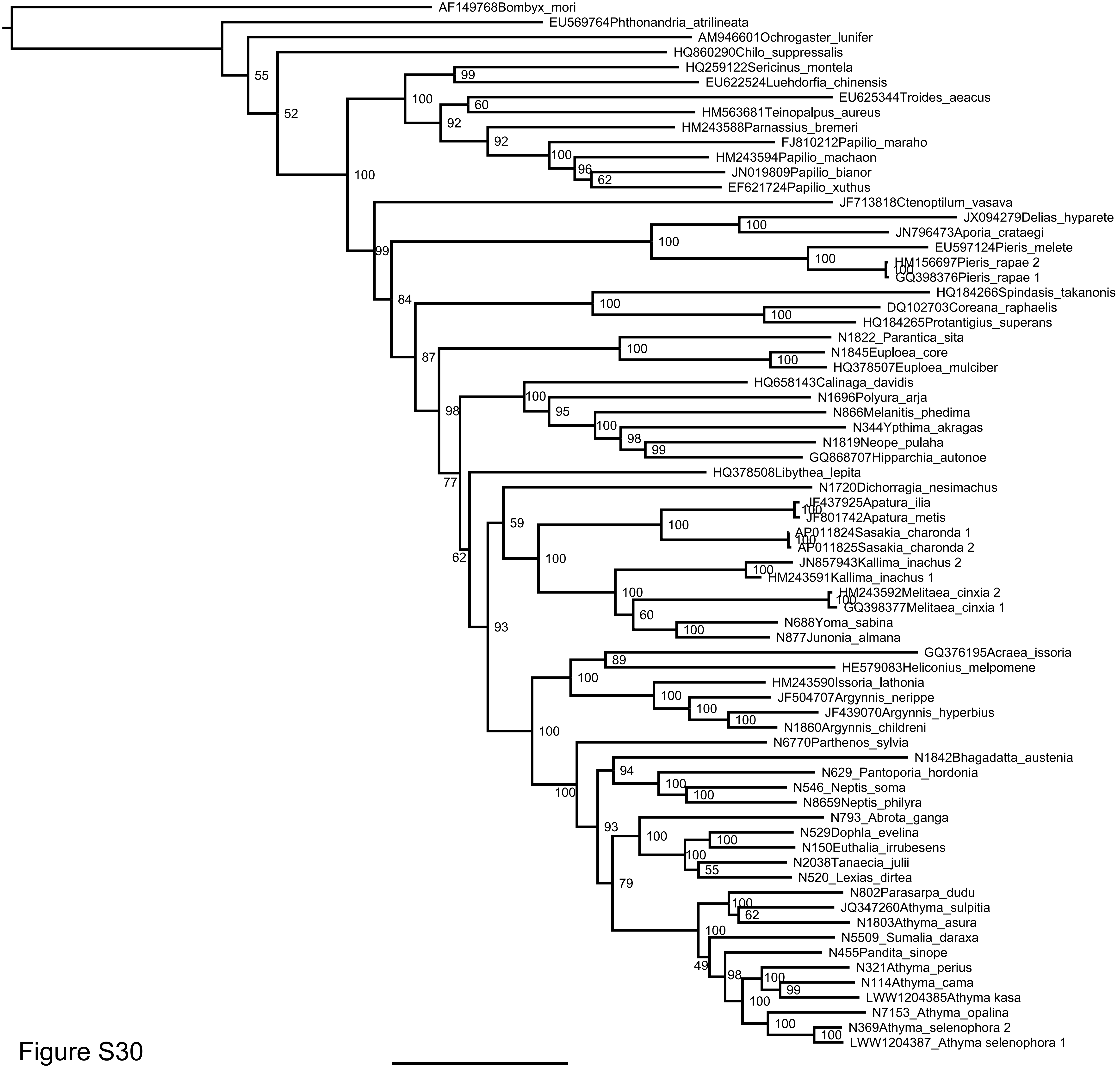

AF149768Bombyx_mori
EU569764Phthonandria_atrilineata
AM946601Ochrogaster_lunifer
HQ860290Chilo_suppressalis
HQ259122Sericinus_montela
EU622524Luehdorfia_chinensis
EU625344Troides_aeacus
HM563681Teinopalpus_aureus
HM243588Parnassius_bremeri
FJ810212Papilio_maraho
HM243594Papilio_machaon
JN019809Papilio_bianor
EF621724Papilio_xuthus
JF713818Ctenoptilum_vasava
JX094279Delias_hyparete
JN796473Aporia_crataegi
EU597124Pieris_melete
HM156697Pieris_rapae 2
GQ398376Pieris_rapae 1
HQ184266Spindasis_takanonis
DQ102703Coreana_raphaelis
HQ184265Protantigius_superans
N1822_Parantica_sita
N1845Euploea_core
HQ378507Euploea_mulciber
HQ658143Calinaga_davidis
N1696Polyura_arja
N866Melanitis_phedima
N344Ypthima_akragas
N1819Neope_pulaha
GQ868707Hipparchia_autonoe
HQ378508Libythea_lepita
N1720Dichorragia_nesimachus
JF437925Apatura_ilia
JF801742Apatura_metis
AP011824Sasakia_charonda 1
AP011825Sasakia_charonda 2
JN857943Kallima_inachus 2
HM243591Kallima_inachus 1
HM243592Melitaea_cinxia 2
GQ398377Melitaea_cinxia 1
N688Yoma_sabina
N877Junonia_almana
GQ376195Acraea_issoria
HE579083Heliconius_melpomene
HM243590Issoria_lathonia
JF504707Argynnis_nerippe
JF439070Argynnis_hyperbius
N1860Argynnis_childreni
N6770Parthenos_sylvia
N1842Bhagadatta_austenia
N629_Pantoporia_hordonia
N546_Neptis_soma
N8659Neptis_philyra
N793_Abrota_ganga
N529Dophla_evelina
N150Euthalia_irrubesens
N2038Tanaecia_julii
N520_Lexias_dirtea
N802Parasarpa_dudu
JQ347260Athyma_sulpitia
N1803Athyma_asura
N5509_Sumalia_daraxa
N455Pandita_sinope
N321Athyma_perius
N114Athyma_cama
LWW1204385Athyma kasa
N7153_Athyma_opalina
N369Athyma_selenophora 2
LWW1204387_Athyma selenophora 1
02

Figure S30

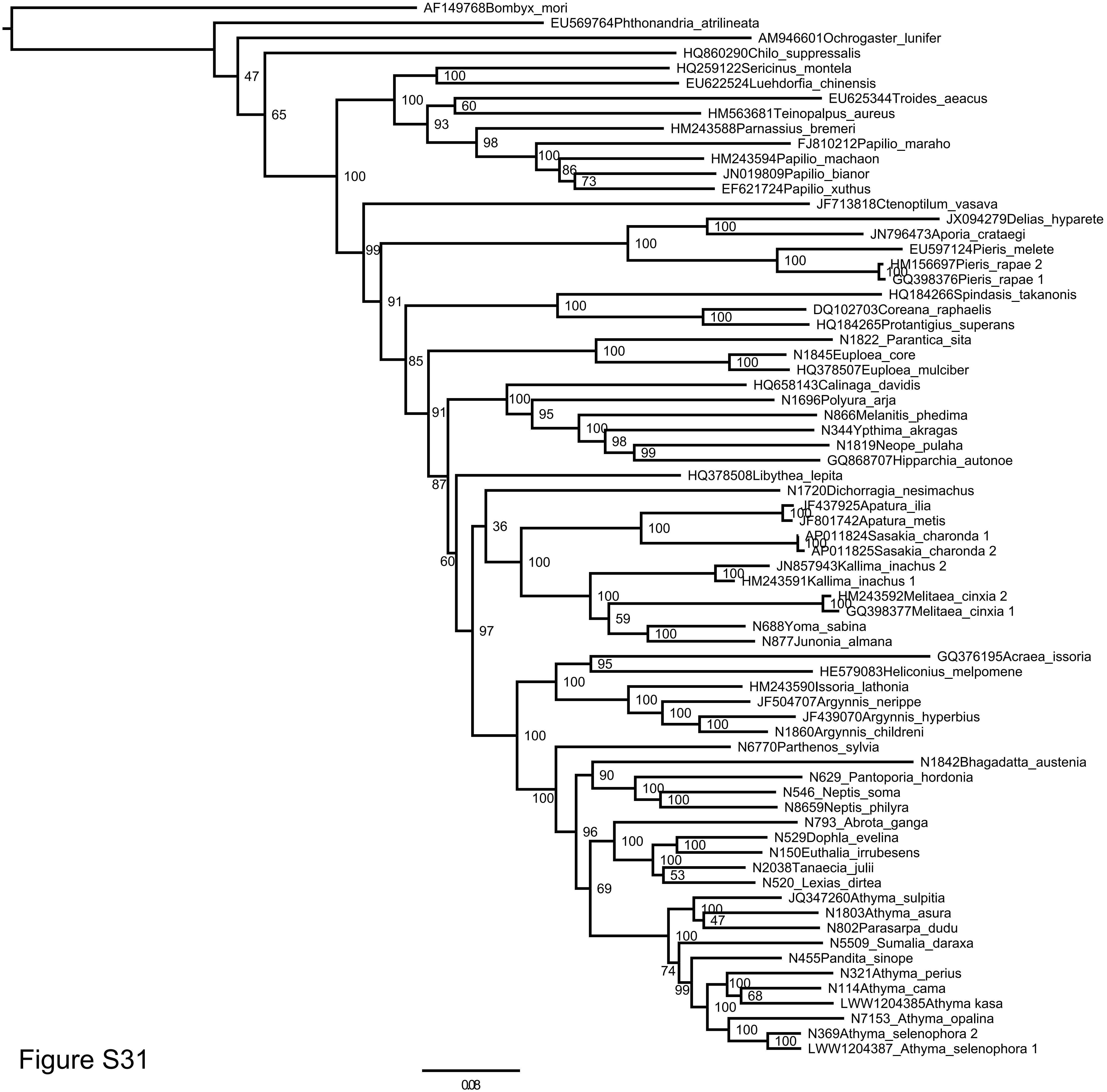
AF149768Bombyx_mori
EU569764Phthonandria_atrilineata
AM946601Ochrogaster_lunifer
HQ860290Chilo_suppressalis
HQ259122Sericinus_montela
EU622524Luehdorfia_chinensis
EU625344Troides_aeacus
HM563681Teinopalpus_aureus
HM243588Parnassius_bremeri
FJ810212Papilio_maraho
HM243594Papilio_machaon
JN019809Papilio_bianor
EF621724Papilio_xuthus
JF713818Ctenoptilum_vasava
JX094279Delias_hyparete
JN796473Aporia_crataegi
EU597124Pieris_melete
HM156697Pieris_rapae 2
GQ398376Pieris_rapae 1
HQ184266Spindasis_takanonis
DQ102703Coreana_raphaelis
HQ184265Protantigius_superans
N1822_Parantica_sita
N1845Euploea_core
HQ378507Euploea_mulciber
HQ658143Calinaga_davidis
N1696Polyura_arja
N866Melanitis_phedima
N344Ypthima_akragas
N1819Neope_pulaha
GQ868707Hipparchia_autonoe
HQ378508Libythea_lepita
N1720Dichorragia_nesimachus
JF437925Apatura_ilia
JF801742Apatura_metis
AP011824Sasakia_charonda 1
AP011825Sasakia_charonda 2
JN857943Kallima_inachus 2
HM243591Kallima_inachus 1
HM243592Melitaea_cinxia 2
GQ398377Melitaea_cinxia 1
N688Yoma_sabina
N877Junonia_almana
GQ376195Acraea_issoria
HE579083Heliconius_melpomene
HM243590Issoria_lathonia
JF504707Argynnis_nerippe
JF439070Argynnis_hyperbius
N1860Argynnis_childreni
N6770Parthenos_sylvia
N1842Bhagadatta_austenia
N629_Pantoporia_hordonia
N546_Neptis_soma
N8659Neptis_philyra
N793_Abrota_ganga
N529Dophla_evelina
N150Euthalia_irrubesens
N2038Tanaecia_julii
N520_Lexias_dirtea
JQ347260Athyma_sulpitia
N1803Athyma_asura
N802Parasarpa_dudu
N5509_Sumalia_daraxa
N455Pandita_sinope
N321Athyma_perius
N114Athyma_cama
LWW1204385Athyma kasa
N7153_Athyma_opalina
N369Athyma_selenophora 2
LWW1204387_Athyma_selenophora 1
0.08

Figure S31

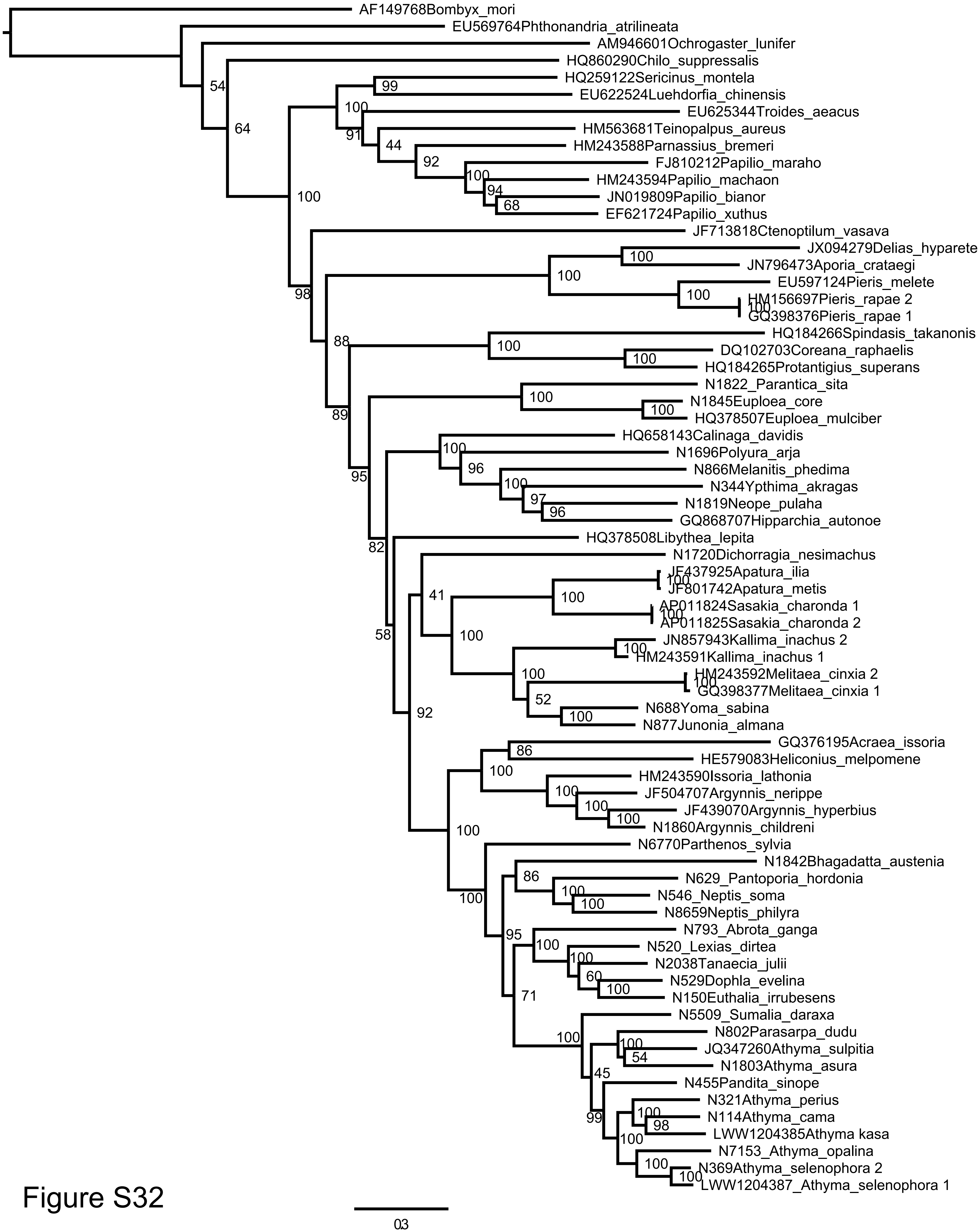
AF149768Bombyx_mori
EU569764Phthonandria_atrilineata
AM946601Ochrogaster_lunifer
HQ860290Chilo_suppressalis
HQ259122Sericinus_montela
EU622524Luehdorfia_chinensis
EU625344Troides_aeacus
HM563681Teinopalpus_aureus
HM243588Parnassius_bremeri
FJ810212Papilio_maraho
HM243594Papilio_machaon
JN019809Papilio_bianor
EF621724Papilio_xuthus
JF713818Ctenoptilum_vasava
JX094279Delias_hyparete
JN796473Aporia_crataegi
EU597124Pieris_melete
HM156697Pieris_rapae 2
GQ398376Pieris_rapae 1
HQ184266Spindasis_takanonis
DQ102703Coreana_raphaelis
HQ184265Protantigius_superans
N1822_Parantica_sita
N1845Euploea_core
HQ378507Euploea_mulciber
HQ658143Calinaga_davidis
N1696Polyura_arja
N866Melanitis_phedima
N344Ypthima_akragas
N1819Neope_pulaha
GQ868707Hipparchia_autonoe
HQ378508Libythea_lepita
N1720Dichorragia_nesimachus
JF437925Apatura_ilia
JF801742Apatura_metis
AP011824Sasakia_charonda 1
AP011825Sasakia_charonda 2
JN857943Kallima_inachus 2
HM243591Kallima_inachus 1
HM243592Melitaea_cinxia 2
GQ398377Melitaea_cinxia 1
N688Yoma_sabina
N877Junonia_almana
GQ376195Acraea_issoria
HE579083Heliconius_melpomene
HM243590Issoria_lathonia
JF504707Argynnis_nerippe
JF439070Argynnis_hyperbius
N1860Argynnis_childreni
N6770Parthenos_sylvia
N1842Bhagadatta_austenia
N629_Pantoporia_hordonia
N546_Neptis_soma
N8659Neptis_philyra
N793_Abrota_ganga
N520_Lexias_dirtea
N2038Tanaecia_julii
N529Dophla_evelina
N150Euthalia_irrubesens
N5509_Sumalia_daraxa
N802Parasarpa_dudu
JQ347260Athyma_sulpitia
N1803Athyma_asura
N455Pandita_sinope
N321Athyma_perius
N114Athyma_cama
LWW1204385Athyma kasa
N7153_Athyma_opalina
N369Athyma_selenophora 2
LWW1204387_Athyma_selenophora 1
03

Figure S32

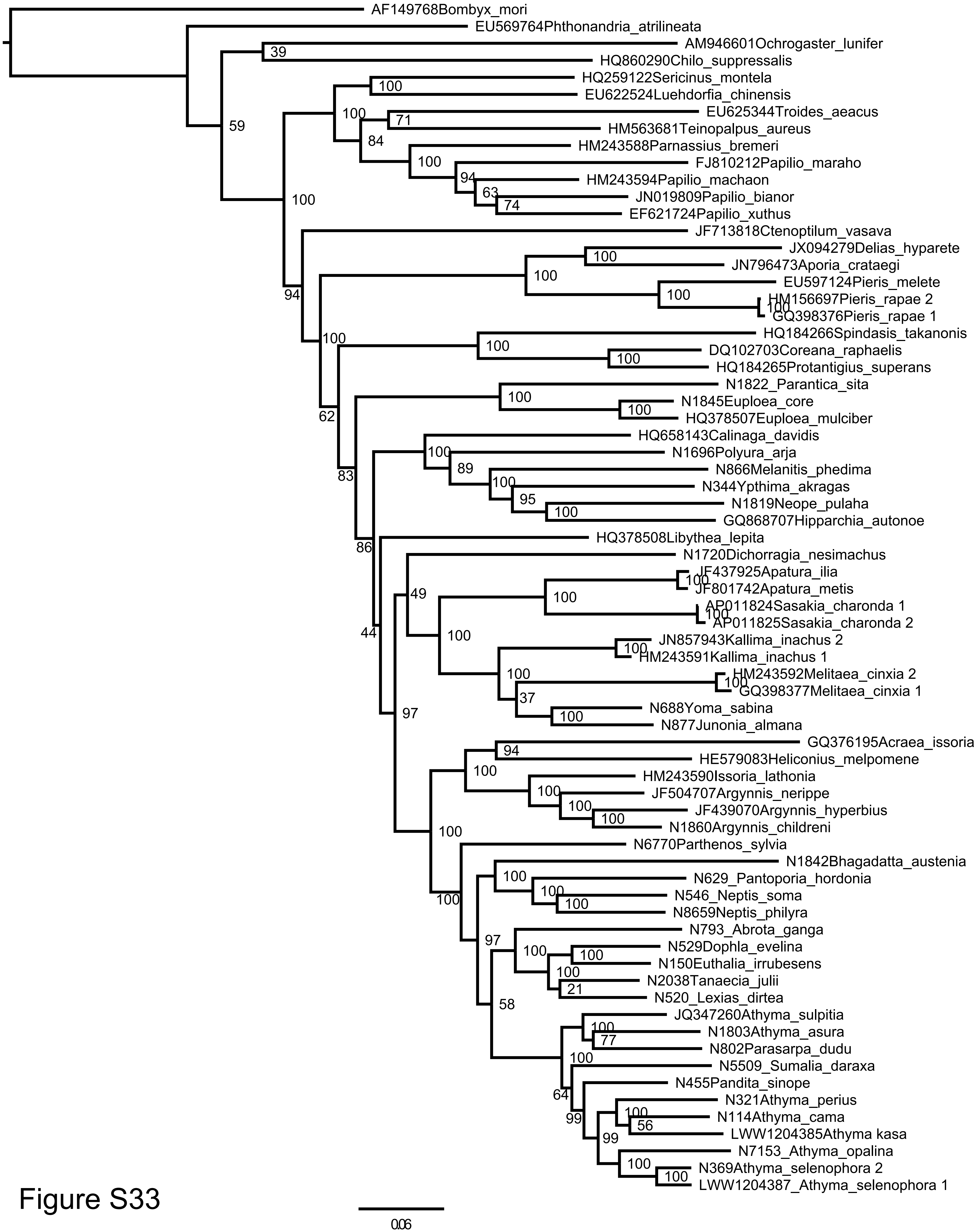
AF149768Bombyx_mori
EU569764Phthonandria_atrilineata
AM946601Ochrogaster_lunifer
HQ860290Chilo_suppressalis
HQ259122Sericinus_montela
EU622524Luehdorfia_chinensis
EU625344Troides_aeacus
HM563681Teinopalpus_aureus
HM243588Parnassius_bremeri
FJ810212Papilio_maraho
HM243594Papilio_machaon
JN019809Papilio_bianor
EF621724Papilio_xuthus
JF713818Ctenoptilum_vasava
JX094279Delias_hyparete
JN796473Aporia_crataegi
EU597124Pieris_melete
HM156697Pieris_rapae 2
GQ398376Pieris_rapae 1
HQ184266Spindasis_takanonis
DQ102703Coreana_raphaelis
HQ184265Protantigius_superans
N1822_Parantica_sita
N1845Euploea_core
HQ378507Euploea_mulciber
HQ658143Calinaga_davidis
N1696Polyura_arja
N866Melanitis_phedima
N344Ypthima_akragas
N1819Neope_pulaha
GQ868707Hipparchia_autonoe
HQ378508Libythea_lepita
N1720Dichorragia_nesimachus
JF437925Apatura_ilia
JF801742Apatura_metis
AP011824Sasakia_charonda 1
AP011825Sasakia_charonda 2
JN857943Kallima_inachus 2
HM243591Kallima_inachus 1
HM243592Melitaea_cinxia 2
GQ398377Melitaea_cinxia 1
N688Yoma_sabina
N877Junonia_almana
GQ376195Acraea_issoria
HE579083Heliconius_melpomene
HM243590Issoria_lathonia
JF504707Argynnis_nerippe
JF439070Argynnis_hyperbius
N1860Argynnis_childreni
N6770Parthenos_sylvia
N1842Bhagadatta_austenia
N629_Pantoporia_hordonia
N546_Neptis_soma
N8659Neptis_philyra
N793_Abrota_ganga
N529Dophla_evelina
N150Euthalia_irrubesens
N2038Tanaecia_julii
N520_Lexias_dirtea
JQ347260Athyma_sulpitia
N1803Athyma_asura
N802Parasarpa_dudu
N5509_Sumalia_daraxa
N455Pandita_sinope
N321Athyma_perius
N114Athyma_cama
LWW1204385Athyma kasa
N7153_Athyma_opalina
N369Athyma_selenophora 2
LWW1204387_Athyma_selenophora 1
0.06

Figure S33

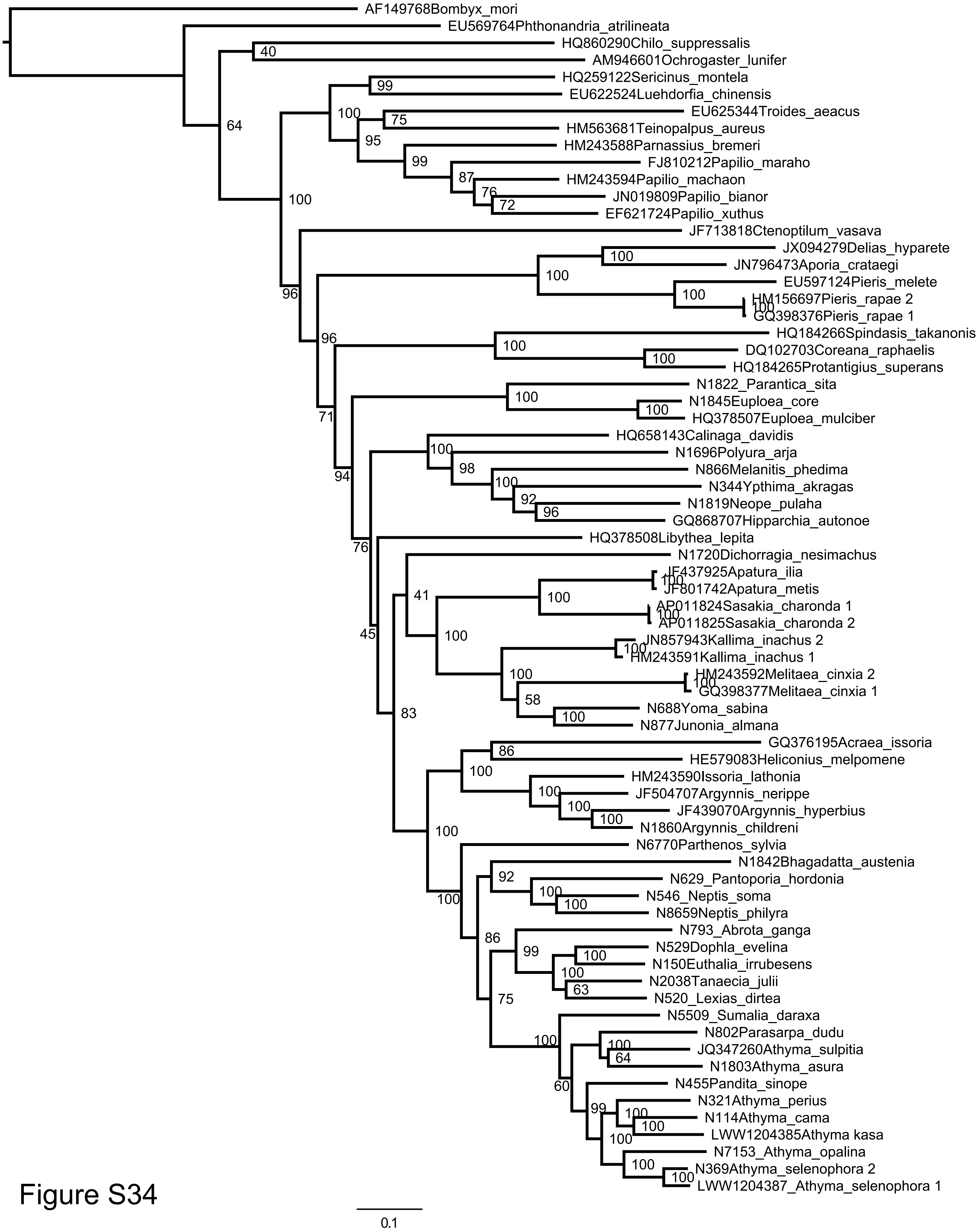
AF149768Bombyx_mori
EU569764Phthonandria_atrilineata
HQ860290Chilo_suppressalis
AM946601Ochrogaster_lunifer
HQ259122Sericinus_montela
EU622524Luehdorfia_chinensis
EU625344Troides_aeacus
HM563681Teinopalpus_aureus
HM243588Parnassius_bremeri
FJ810212Papilio_maraho
HM243594Papilio_machaon
JN019809Papilio_bianor
EF621724Papilio_xuthus
JF713818Ctenoptilum_vasava
JX094279Delias_hyparete
JN796473Aporia_crataegi
EU597124Pieris_melete
HM156697Pieris_rapae 2
GQ398376Pieris_rapae 1
HQ184266Spindasis_takanonis
DQ102703Coreana_raphaelis
HQ184265Protantigius_superans
N1822_Parantica_sita
N1845Euploea_core
HQ378507Euploea_mulciber
HQ658143Calinaga_davidis
N1696Polyura_arja
N866Melanitis_phedima
N344Ypthima_akragas
N1819Neope_pulaha
GQ868707Hipparchia_autonoe
HQ378508Libythea_lepita
N1720Dichorragia_nesimachus
JF437925Apatura_ilia
JF801742Apatura_metis
AP011824Sasakia_charonda 1
AP011825Sasakia_charonda 2
JN857943Kallima_inachus 2
HM243591Kallima_inachus 1
HM243592Melitaea_cinxia 2
GQ398377Melitaea_cinxia 1
N688Yoma_sabina
N877Junonia_almana
GQ376195Acraea_issoria
HE579083Heliconius_melpomene
HM243590Issoria_lathonia
JF504707Argynnis_nerippe
JF439070Argynnis_hyperbius
N1860Argynnis_childreni
N6770Parthenos_sylvia
N1842Bhagadatta_austenia
N629_Pantoporia_hordonia
N546_Neptis_soma
N8659Neptis_philyra
N793_Abrota_ganga
N529Dophla_evelina
N150Euthalia_irrubesens
N2038Tanaecia_julii
N520_Lexias_dirtea
N5509_Sumalia_daraxa
N802Parasarpa_dudu
JQ347260Athyma_sulpitia
N1803Athyma_asura
N455Pandita_sinope
N321Athyma_perius
N114Athyma_cama
LWW1204385Athyma kasa
N7153_Athyma_opalina
N369Athyma_selenophora 2
LWW1204387_Athyma_selenophora 1
0.1

Figure S34

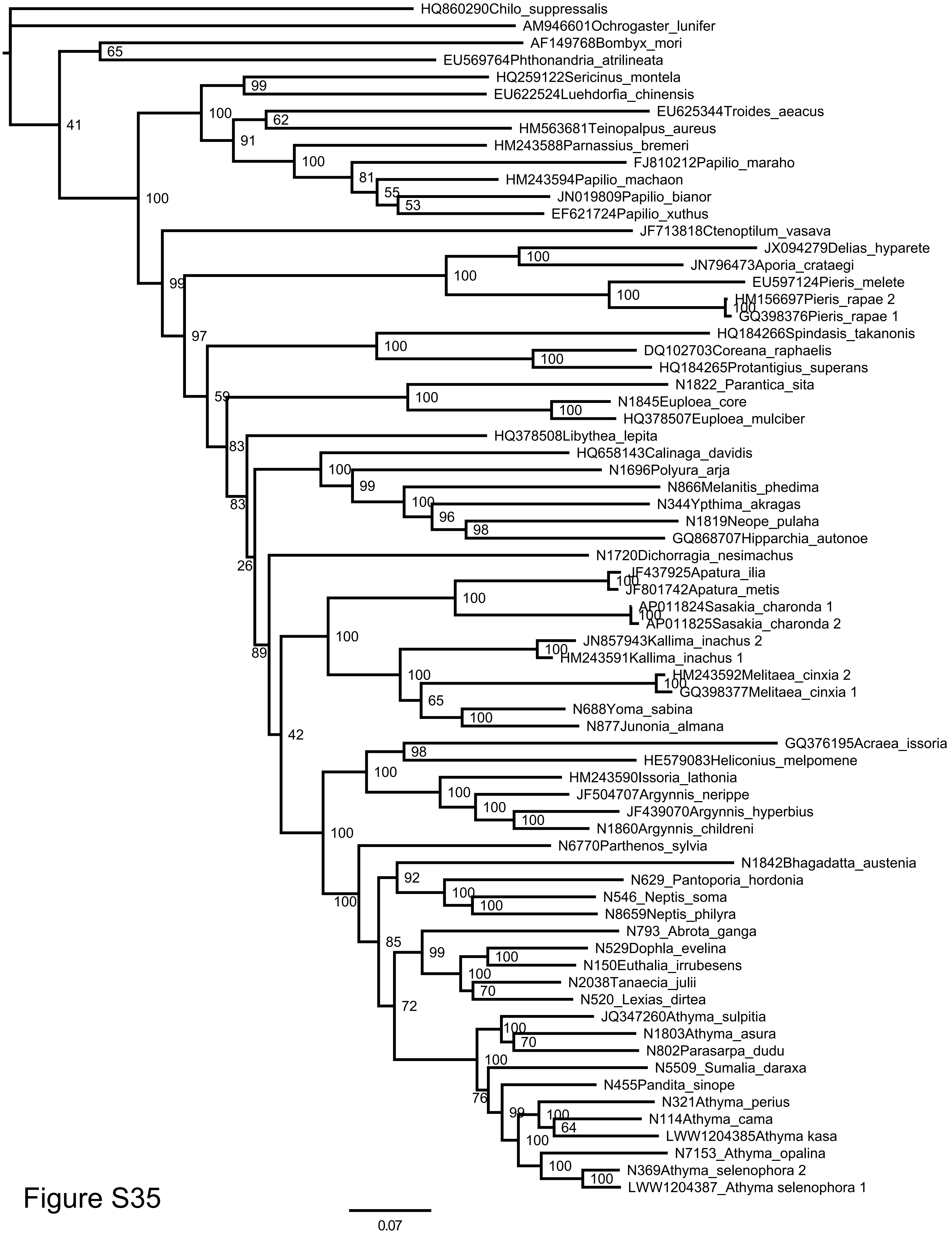
HQ860290Chilo_suppressalis
AM946601Ochrogaster_lunifer
AF149768Bombyx_mori
EU569764Phthonandria_atrilineata
HQ259122Sericinus_montela
EU622524Luehdorfia_chinensis
EU625344Troides_aeacus
HM563681Teinopalpus_aureus
HM243588Parnassius_bremeri
FJ810212Papilio_maraho
HM243594Papilio_machaon
JN019809Papilio_bianor
EF621724Papilio_xuthus
JF713818Ctenoptilum_vasava
JX094279Delias_hyparete
JN796473Aporia_crataegi
EU597124Pieris_melete
HM156697Pieris_rapae 2
GQ398376Pieris_rapae 1
HQ184266Spindasis_takanonis
DQ102703Coreana_raphaelis
HQ184265Protantigius_superans
N1822_Parantica_sita
N1845Euploea_core
HQ378507Euploea_mulciber
HQ378508Libythea_lepita
HQ658143Calinaga_davidis
N1696Polyura_arja
N866Melanitis_phedima
N344Ypthima_akragas
N1819Neope_pulaha
GQ868707Hipparchia_autonoe
N1720Dichorragia_nesimachus
JF437925Apatura_ilia
JF801742Apatura_metis
AP011824Sasakia_charonda 1
AP011825Sasakia_charonda 2
JN857943Kallima_inachus 2
HM243591Kallima_inachus 1
HM243592Melitaea_cinxia 2
GQ398377Melitaea_cinxia 1
N688Yoma_sabina
N877Junonia_almana
GQ376195Acraea_issoria
HE579083Heliconius_melpomene
HM243590Issoria_lathonia
JF504707Argynnis_nerippe
JF439070Argynnis_hyperbius
N1860Argynnis_childreni
N6770Parthenos_sylvia
N1842Bhagadatta_austenia
N629_Pantoporia_hordonia
N546_Neptis_soma
N8659Neptis_philyra
N793_Abrota_ganga
N529Dophla_evelina
N150Euthalia_irrubesens
N2038Tanaecia_julii
N520_Lexias_dirtea
JQ347260Athyma_sulpitia
N1803Athyma_asura
N802Parasarpa_dudu
N5509_Sumalia_daraxa
N455Pandita_sinope
N321Athyma_perius
N114Athyma_cama
LWW1204385Athyma kasa
N7153_Athyma_opalina
N369Athyma_selenophora 2
LWW1204387_Athyma selenophora 1
0.07

Figure S35

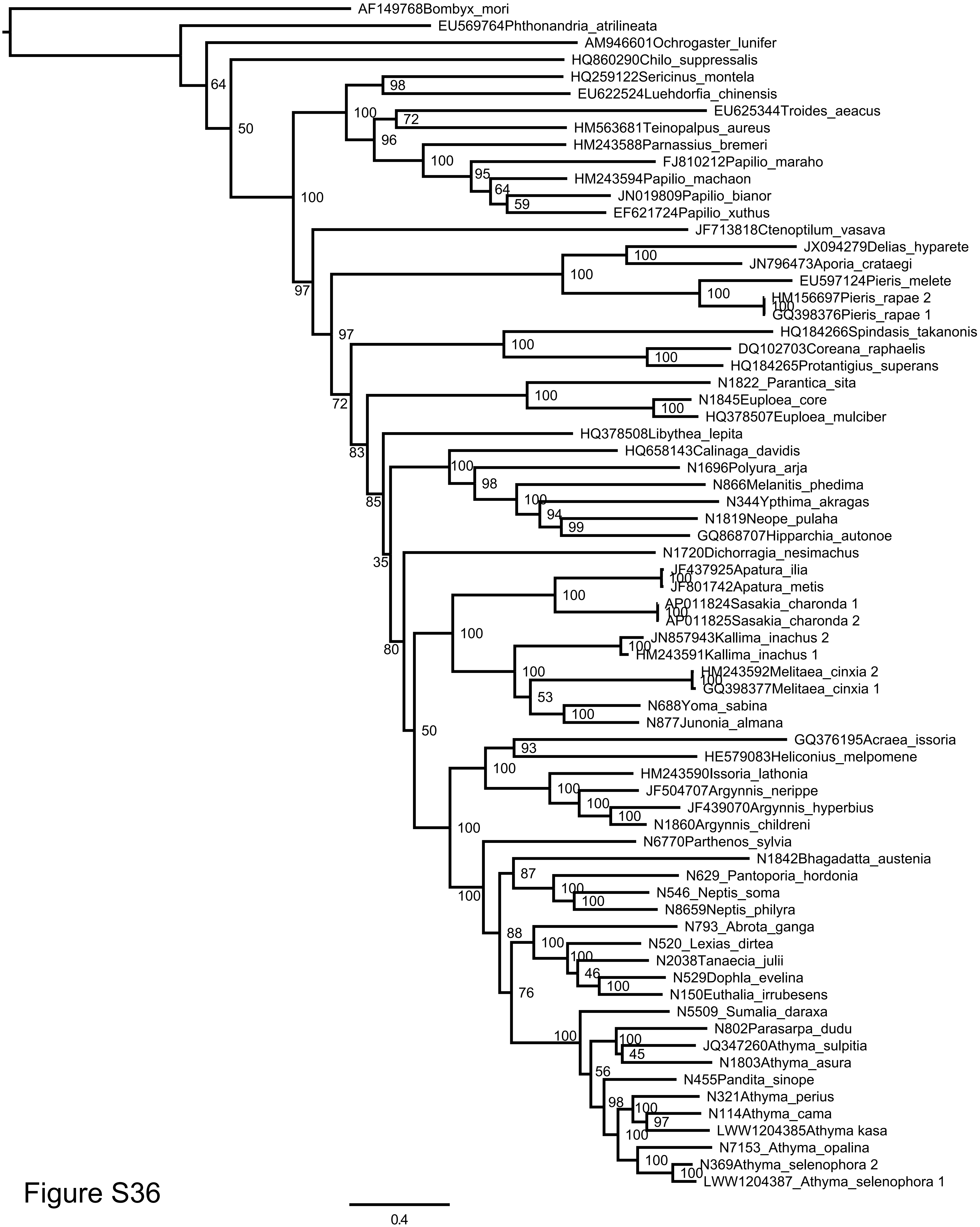
AF149768Bombyx_mori
EU569764Phthonandria_atrilineata
AM946601Ochrogaster_lunifer
HQ860290Chilo_suppressalis
HQ259122Sericinus_montela
EU622524Luehdorfia_chinensis
EU625344Troides_aeacus
HM563681Teinopalpus_aureus
HM243588Parnassius_bremeri
FJ810212Papilio_maraho
HM243594Papilio_machaon
JN019809Papilio_bianor
EF621724Papilio_xuthus
JF713818Ctenoptilum_vasava
JX094279Delias_hyparete
JN796473Aporia_crataegi
EU597124Pieris_melete
HM156697Pieris_rapae 2
GQ398376Pieris_rapae 1
HQ184266Spindasis_takanonis
DQ102703Coreana_raphaelis
HQ184265Protantigius_superans
N1822_Parantica_sita
N1845Euploea_core
HQ378507Euploea_mulciber
HQ378508Libythea_lepita
HQ658143Calinaga_davidis
N1696Polyura_arja
N866Melanitis_phedima
N344Ypthima_akragas
N1819Neope_pulaha
GQ868707Hipparchia_autonoe
N1720Dichorragia_nesimachus
JF437925Apatura_ilia
JF801742Apatura_metis
AP011824Sasakia_charonda 1
AP011825Sasakia_charonda 2
JN857943Kallima_inachus 2
HM243591Kallima_inachus 1
HM243592Melitaea_cinxia 2
GQ398377Melitaea_cinxia 1
N688Yoma_sabina
N877Junonia_almana
GQ376195Acraea_issoria
HE579083Heliconius_melpomene
HM243590Issoria_lathonia
JF504707Argynnis_nerippe
JF439070Argynnis_hyperbius
N1860Argynnis_childreni
N6770Parthenos_sylvia
N1842Bhagadatta_austenia
N629_Pantoporia_hordonia
N546_Neptis_soma
N8659Neptis_philyra
N793_Abrota_ganga
N520_Lexias_dirtea
N2038Tanaecia_julii
N529Dophla_evelina
N150Euthalia_irrubesens
N5509_Sumalia_daraxa
N802Parasarpa_dudu
JQ347260Athyma_sulpitia
N1803Athyma_asura
N455Pandita_sinope
N321Athyma_perius
N114Athyma_cama
LWW1204385Athyma kasa
N7153_Athyma_opalina
N369Athyma_selenophora 2
LWW1204387_Athyma_selenophora 1
0.4

Figure S36
